# Graft-Specific Neuromuscular Responses and EMG Activities During Running Before and After Running-Induced Fatigue in Individuals with ACL Reconstruction

**DOI:** 10.3390/s26154904

**Published:** 2026-08-03

**Authors:** Heidar Sajedi, Mahsa Habibi, AmirAli Jafarnezhadgero, Ebrahim Piri, Kate E Webster, Valdeci Carlos Dionisio

**Affiliations:** 1 Department of Movement and Training Sciences, Sport Science Faculty, Düzce University, 81000 Duzce, Turkey; 2 Department of Sports Biomechanics, Faculty of Educational Sciences and Psychology, University of Mohaghegh Ardabili, Ardabil P.O. Box 179, Iran; 3 School of Allied Health, Human Services and Sport, La Trobe University, Bundoora, Melbourne 3550, VIC, Australia; 4 Faculty of Physical Education and Physiotherapy, Federal University of Uberlandia, Uberlandia 38408-100, MG, Brazil

**Keywords:** anterior cruciate ligament, reconstruction, graft type, fatigue, electromyography, running

## Abstract

**Highlights:**

**What are the main findings?**

- Fatigue reduced semitendinosus and gluteus medius activation during early stance regardless of group. The only significant group × fatigue interaction was observed for vastus medialis, showing a fatigue-related reduction in the DB-HT group.
- During late stance, significant group effects were found for tibialis anterior, gastrocnemius, rectus femoris, biceps femoris, semitendinosus, and gluteus medius.

**What are the implications of the main findings?**

- Graft type was associated with distinct lower-limb EMG activation patterns during running after ACLR. Fatigue revealed a DB-HT-specific reduction in vastus medialis activation, while the allograft and SB-HT groups showed different compensatory activation profiles.
- These findings support graft-specific, fatigue-based rehabilitation strategies aimed at improving functional stability and potentially reducing longer-term joint loading risk.

**Abstract:**

Background: Fatigue during intense activity challenges the neuromuscular system and can reveal deficits not evident during lower-demand functional tasks. In individuals with anterior cruciate ligament reconstruction (ACLR), differences in graft source may be associated with graft-specific neuromuscular adaptations. Although fatigue-related neuromuscular deficits after ACLR have been reported, few studies have examined whether these responses differ according to graft type during running. This study investigated the effects of running-induced fatigue on lower-limb muscle activity during the stance phase in ACLR patients with different graft types, compared with healthy controls. Methods: Fifty-three active males were allocated into four groups: single-bundle hamstring tendon (SB-HT), double-bundle hamstring tendon (DB-HT), allograft, and healthy controls. Participants completed a treadmill-based fatigue protocol that was terminated when they reached a Borg score > 17, which served as the primary criterion for confirming a high level of perceived fatigue. Heart rate ≥ 85% of estimated maximum heart rate was recorded as a complementary physiological indicator of high-intensity exertion. Electromyography (EMG) amplitudes of eight lower-limb muscles were recorded during the early and late stance phases of running, before and after fatigue. A two-way mixed-model ANOVA evaluated the effects of graft type and fatigue condition. Results: Fatigue reduced semitendinosus (*p* = 0.042, ηp^2^ = 0.091) and gluteus medius (*p* < 0.001, ηp^2^ = 0.442) activation during early stance regardless of group. Late-stance group effects were significant for the tibialis anterior, gastrocnemius, rectus femoris, biceps femoris, semitendinosus, and gluteus medius (*p* ≤ 0.05). Across stance phases, the SB-HT group showed greater hamstring and gluteus medius activation than healthy controls (*p* = 0.003–0.041), while the allograft group showed greater tibialis anterior, gastrocnemius, and rectus femoris activation during late stance (*p* = 0.005–0.043). The only significant group × fatigue interaction was observed for vastus medialis (*p* = 0.002, ηp^2^ = 0.279), reflecting a fatigue-related reduction in the DB-HT group. Conclusions: Graft type was associated with distinct lower-limb EMG activation patterns during running after ACLR. Fatigue revealed a DB-HT-specific reduction in vastus medialis activation, while the allograft and SB-HT groups showed different compensatory activation profiles. These findings suggest that graft-specific neuromuscular adaptations during fatigue should be considered when developing rehabilitation protocols to improve functional stability and future joint health.

## 1. Introduction

The knee is a primary load-bearing joint prone to injury during activities like running and jumping. Anterior cruciate ligament reconstruction (ACLR) is often performed to restore stability and facilitate return-to-sport (RTS) [1,2]. However, recovery is often incomplete. Only about 65% of athletes return to their pre-injury level [3,4,5]. Persistent neuromuscular deficits may remain despite satisfactory clinical outcomes.

Previous research shows that individuals following ACLR exhibit altered lower-limb biomechanics, including reduced knee flexion, increased abduction, and compensatory hip and ankle strategies during tasks. These biomechanical changes are observable outcomes of underlying neuromuscular control processes. Electromyography (EMG) offers a direct assessment of neural drive and muscle recruitment, providing critical insights into central nervous system coordination after injury and reconstruction. In healthy individuals, fatigue impacts quadriceps and hamstring (HT) activation and can compromise joint stability [6,7]. In reconstructed knees, the effect may be amplified, as the sensorimotor system compensates for altered ligaments, proprioception, and motor control. Therefore, fatigue acts as a “functional stress test,” exposing hidden neuromuscular impairments that routine assessments might miss.

An additional factor that may influence neuromuscular control after ACLR is the type of graft used during reconstruction. Common techniques include single-bundle HT tendon (SB-HT), double-bundle HT tendon (DB-HT), and allograft reconstructions, each with distinct biomechanical and biological characteristics [8]. While HT autografts are widely used due to lower donor-site morbidity, differences in graft structure, surgical complexity, and biological integration may lead to graft-specific adaptations in neuromuscular control. HT autografts commonly involve harvesting the semitendinosus and gracilis tendons, which may influence medial HT function after ACLR. Double-bundle reconstructions aim to restore both anteromedial and posterolateral bundle function, which may alter rotational stability demands. Allografts avoid donor site morbidity but differ in biological incorporation and sensory recovery. These differences may influence how the neuromuscular system stabilizes the knee during loading. A previous study showed that the HT autograft group had reduced knee varus rotation compared to both the patellar tendon autograft group and the healthy control group during walking [9]. Sinding et al. reported that one year after ACLR, muscle strength is affected by autograft type, with the semitendinosus–gracilis autograft leading to impairments in knee extensor and flexor muscle strength, whereas the quadriceps tendon autograft results in more pronounced impairments in knee extensor strength only [10]. Functional capacity and patient-reported outcomes were unaffected by autograft type, although functional capacity was impaired relative to healthy controls [10]. Deficits in HT strength were reported in the semitendinosus–double strand gracilis graft groups when compared to the quadruple strand semitendinosus graft groups in isometric strength testing at knee flexion angles ≥70°, and in the standing knee flexion angle [11]. Another study demonstrated significant within-group improvements in contraction time and maximal displacement in the rectus femoris, vastus medialis, vastus lateralis, and biceps femoris muscles across all graft types from 3 to 9 months [12]. The quadriceps tendon group showed the most pronounced contraction time reduction in rectus femoris and vastus medialis [12]. In contrast, HT tendon grafts demonstrated limited recovery of biceps femoris tendon contraction time between 6 and 9 months compared with bone–patellar tendon–bone and quadriceps tendon grafts, indicating a stagnation phase [12]. Bone–patellar tendon–bone exhibited persistent bilateral deficits in both quadriceps and biceps femoris at 9 months [12]. Patients with HT autografts may experience increased anterior tibial translation and decreased flexion strength compared with those treated with bone–patellar tendon–bone or quadriceps tendon grafts [13]. Currently, the literature shows no difference in RTS rates between bone–patellar tendon–bone and HT grafts [13,14,15]. While patient-reported outcomes are not influenced by graft choice, evidence suggests lower postoperative graft rupture rates in patients treated with bone–patellar tendon–bone and quadriceps tendon autografts than in those treated with HT tendon grafts or allografts [13]. Postoperative donor-site morbidity is highest with bone–patellar tendon–bone grafts, comparable between HT and quadriceps tendon grafts, and absent in allografts [13]. However, limited evidence exists regarding how different graft types affect muscle activation strategies, particularly under fatigued conditions that closely mimic sport-specific demands.

Running, a common and accessible physical activity, provides an ecologically valid context to investigate these mechanisms. It imposes repetitive mechanical loads and requires precise neuromuscular coordination, especially during the stance phase, where joint stability is critical. Understanding how fatigue influences EMG activity during running in individuals with different ACL graft types is essential for identifying graft-dependent neuromuscular strategies and potential vulnerabilities. Therefore, this study aimed to investigate the effects of a running-induced fatigue protocol on lower-limb EMG amplitude during the stance phase in individuals with ACLR, comparing SB-HT, DB-HT, and allograft reconstructions with healthy controls. We hypothesized that fatigue would produce graft-specific changes in EMG amplitude during stance, particularly in the quadriceps, HT, and gastrocnemius muscles, with the greatest alterations expected during the weight-acceptance and late-stance phases, reflecting distinct strategies for maintaining knee stability under physical stress.

## 2. Materials and Methods

### 2.1. Participants

This cross-sectional laboratory study was conducted in accordance with the Declaration of Helsinki and approved by the Ethics Committee of the University of Mohaghegh Ardabili, Iran (IR.UMA.REC.1404.078). The study examined how a running-induced fatigue protocol affects EMG activity during running in individuals with ACLR, considering different graft types. Fifty-three active males aged 18 to 30 volunteered. The ACLR group included 38 individuals who had primary unilateral ACLR, divided into three groups based on graft type: SB-HT, DB-HT, and allograft reconstruction. A healthy control group of 15 males, matched for age and activity level and without a history of lower-limb injury or surgery, was also included for comparison. A priori power analysis was conducted using G*Power (Version 3.1.9.2) to ensure an adequately powered sample. Based on an F-test for the within–between interaction in a repeated-measures ANOVA, with an effect size f = 0.33, a statistical power of 0.80, and an alpha level of 0.05, the required total sample size was estimated to be 32 participants. However, 53 participants were ultimately included in the study. This effect size was derived from a previous study on lower-extremity neuromuscular activity during running in ACL-reconstructed individuals, which calculated an effect size f = 0.33 for semitendinosus muscle activity [16]. To be included in the ACLR groups, volunteers (recruited from clinics and sports clubs) were required to have a primary unilateral ACLR with SB-HT, DB-HT, or allograft (with allografts being either tibialis anterior or tibialis posterior tendons, confirmed by surgical records), a postoperative period between 8 and 14 months, participation in a standardized postoperative rehabilitation program supervised by a physical therapist, and no concomitant ligament injury or meniscal involvement requiring meniscectomy. Volunteers with any history of lower-limb fracture, neurological disorder, or cardiovascular contraindications to maximal exercise were excluded from the study. All participants in the ACLR groups completed a standardized postoperative rehabilitation program under the supervision of a physical therapist at the same clinic [17]. The program was criterion-based and progressed through early range-of-motion (ROM) and swelling-control exercises, strengthening and neuromuscular training, proprioceptive and balance exercises, and finally running and RTS preparation. Rehabilitation was typically performed in 3 sessions per week [6], with progression based on clinical recovery, including pain-free full ROM, absence of joint effusion, restoration of quadriceps and HT strength, and adequate functional performance. Before inclusion in the present study, all ACLR participants had returned to running and had been cleared for sport participation by the treating orthopedic surgeon and rehabilitation team.

Moreover, all participants had been cleared to resume running and sport participation by their treating orthopedic surgeon and rehabilitation team, based on clinical recovery and functional criteria, including absence of pain and joint effusion, full ROM [18], adequate quadriceps and HT strength (limb symmetry index ≥ 90% on isokinetic testing) [19], single-leg hop tests (limb symmetry index ≥ 90%), Landing Error Scoring System (LESS < 5), and satisfactory scores on patient-reported outcome measures including the International Knee Documentation Committee (IKDC) subjective knee form and the ACL-Return to Sport after Injury (ACL-RSI) scale [20]. For participants included in the study, lower-limb dominance was determined using a two-step method. In the ACLR groups, the reconstructed limb was selected for EMG analysis; in the healthy control group, the dominant limb was analyzed. First, the preferred kicking limb was recorded and then confirmed with a single-leg hop test for distance [21]. The distribution of dominant limbs across groups is shown in Table 1. Participants also completed the Tegner Activity Scale to assess activity levels, which range from 0 (disability) to 10 (elite sports) [22]. No significant differences were found between groups in demographic, anthropometric, activity-related, or foot-posture variables (*p* > 0.05), indicating comparable baseline characteristics.

### 2.2. Surgical Procedure Characteristics

All ACLR procedures were performed arthroscopically by a single experienced orthopedic surgeon using a standardized surgical technique, ensuring consistency in operative management across participants. The SB-HT group received an autologous quadruple-looped semitendinosus tendon graft; the DB-HT group received an autologous semitendinosus and gracilis tendon graft configured as a double bundle; and the allograft group underwent reconstruction using donor tibialis anterior or tibialis posterior tendons, as confirmed by surgical records. Postoperative radiographs confirmed correct tunnel placement, with the femoral tunnel consistently positioned within the native ACL femoral footprint to support the restoration of rotational knee stability. For HT grafts, femoral fixation was achieved using an Endobutton or suspensory fixation device (Smith & Nephew, Andover, MA, USA), and tibial fixation was performed using a bioabsorbable interference screw. Graft diameters ranged between 7.5 and 9.0 mm. Regarding associated injuries, 12 patients underwent concomitant partial meniscectomy and 8 underwent meniscal repair, while patients with high-grade chondral lesions (Outerbridge grade III or IV) were excluded. The reconstructed knees included 28 right and 10 left knees. The choice of graft type was determined based on the surgeon’s clinical judgment, the patient’s pre-injury activity level, and individual anatomical considerations [23].

### 2.3. Fatigue Protocol

The fatigue protocol consisted of running on a motorized treadmill (Horizon Fitness Omega GT, Cottage Grove, WI, USA) set at 0% grade, with participants wearing standardized shoes. Heart rate was continuously monitored using a Polar RS100 device (Polar Electro Oy, Woodbury, NY, USA). The session started at 6 km/h, increasing by 1 km/h every two minutes. After each stage, participants rated their perceived exertion on the 6–20 Borg scale [24]. Once a Borg score of 13 or higher was reached, treadmill speed was maintained to allow for steady-state running, with heart rate and perceived exertion recorded every 30 s. Maximum heart rate was estimated using the formula 220 minus age [25]. While acknowledging the inherent individual-level variability of this formula, it was used here in combination with the Borg Rating of Perceived Exertion (RPE) to provide a multidimensional confirmation of high-intensity fatigue. The fatigue protocol was terminated once participants reached a Borg score > 17, which served as the primary criterion for confirming a high level of perceived fatigue. A heart rate ≥ 85% of estimated maximum heart rate was recorded as a complementary physiological indicator of high-intensity exertion. All participants reached the Borg criterion, whereas two participants did not reach the heart rate threshold. These participants were retained in the study because they met the primary fatigue criterion. Immediately after completion of the fatigue protocol, running assessments were conducted. This running-based fatigue assessment was chosen because it is feasible for individuals with ACLR who have completed rehabilitation [26,27].

Group-specific fatigue protocol outcomes, including total protocol duration, final treadmill speed, absolute and relative final heart rate, final Borg score, localized fatigue, perceived knee instability/discomfort, and the number of participants meeting each termination criterion, are presented in Table 2.

### 2.4. EMG Data Collection During Running

All biomechanical tests were conducted in a climate-controlled lab maintained at 23–24 °C and 45–50% humidity. An EMG system (EMG Pre-Amplifier, Biometrics Ltd., Nine Mile Point Ind., Newport, UK) with eight pairs of bipolar Ag/AgCl surface electrodes (25 mm apart; input impedance of 100 MΩ; common-mode rejection >110 dB) recorded activity from the reconstructed limb in participants with ACLR and from the dominant limb in healthy controls, including the tibialis anterior (TA), gastrocnemius medialis (GC), biceps femoris (BF), semitendinosus (ST), vastus lateralis (VL), vastus medialis (VM), rectus femoris (RF), and gluteus medius (GM) muscles [28]. The skin was gently abraded before electrode placement [29], and the electrodes were attached to muscle bellies with die-cut, medical-grade, double-sided adhesive tape (T350, Biometrics Ltd., Newport, UK). The raw EMG signals were digitized at 1000 Hz and streamed via Bluetooth to a computer for analysis. According to European surface EMG guidelines (SENIAM), skin over the muscles was shaved and cleaned with 70% ethanol (C_2_H_5_OH) before electrode placement.

During EMG analysis, the running stance phase was divided into two segments: the early stance phase (0–50%) and the late stance phase (50–100%) [29,30,31,32]. Surface EMG signals were collected from the TA, GC, BF, ST, VL, VM, RF, and GM muscles at a sampling frequency of 1000 Hz and normalized to maximum voluntary isometric contraction (MVIC). EMG signals were band-pass filtered using a fourth-order zero-lag Butterworth filter (10–500 Hz), followed by full-wave rectification. A 150 ms moving RMS window was used to reduce signal variability while preserving the temporal characteristics of muscle activation, consistent with previous EMG processing recommendations [33,34]. Initial contact and toe-off events were identified using a Bertec force plate (Columbus, OH, USA). Initial contact was identified when the vertical ground reaction force exceeded 20 N. Data from three running trials were averaged for statistical analysis. Intra-session reliability was assessed using the intraclass correlation coefficient (ICC), yielding > 0.92. Analyses were performed on the dominant limb in the healthy group and on the reconstructed limb in the other groups. The stance phase was detected using vertical ground reaction force data (threshold = 10 N). Ground reaction forces were recorded by a force platform (Bertec, USA). Ground reaction force and EMG data were synchronized. A handheld dynamometer was used to assess maximum voluntary isometric contraction (MVIC) for each muscle to normalize EMG data during running (Supplementary File S1). Before data collection, participants completed a familiarization session consisting of practice contractions for each muscle. All normalization procedures followed the guidelines of Besomi et al. (2020), with participants encouraged to exert maximum effort [35]. Three MVIC trials were recorded for each muscle, with each contraction held for 5 s and a 60 s rest interval between trials to minimize fatigue. The EMG signal from each trial was processed, and the mean RMS value calculated over the central 3 s window (excluding the initial and final 1 s to avoid force ramp-up and relaxation effects) was used to determine the representative MVIC. The highest RMS value obtained across the three trials was selected as the reference MVIC, and all gait EMG amplitudes were expressed as a percentage of this value (%MVIC) [35].

Participants ran along an 18 m runway at a controlled speed of 3.0 ± 0.1 m/s, chosen based on previous biomechanics studies with physically active individuals and post-ACLR populations. Running speed was continuously monitored, and auditory cues from a metronome helped participants keep the desired pace. A single experienced investigator performed all signal processing and variable extraction to minimize inter-rater variability. Before the trials, participants completed three practice runs for procedural consistency. Then, three valid trials were recorded, with a one-minute rest between each to reduce fatigue. Data processing (e.g., band pass filter 10–500 Hz) adhered to established protocols [36].

### 2.5. Statistical Analysis

Normality was tested with the Shapiro–Wilk test. Sphericity was checked using Mauchly’s test, with Greenhouse–Geisser corrections applied as needed. A two-way mixed-model repeated-measures ANOVA was performed, with fatigue condition (non-fatigued vs. fatigued) as the within-subject factor and graft type (SB-HT, DB-HT, allograft, healthy) as the between-subject factor. Intra-session reliability between trials was assessed using the ICC, and the corresponding values for each muscle during the early and late stance phases before and after fatigue are presented in Supplementary File S1. A priori power analysis was performed using G*Power (version 3.1.9.2) for the within–between interaction in a repeated-measures ANOVA. The analysis assumed an effect size of f = 0.33, four groups, two measurements, an α level of 0.05, a desired statistical power (1 − β) of 0.80, a correlation of 0.50 between repeated measurements, and a nonsphericity correction (ε) of 1. Based on these assumptions, the minimum required total sample size was estimated to be 32 participants to detect the prespecified interaction effect. Post hoc comparisons with Bonferroni adjustments were used when relevant. Therefore, the Bonferroni-adjusted significance threshold was α = 0.05/16 = 0.0031. The *p*-values reported in the Results are Bonferroni-adjusted *p*-values rather than raw *p*-values. Effect sizes were reported as partial eta squared (η^2^p) and interpreted using standard thresholds [37]. Statistical significance was set at *p* < 0.05.

## 3. Results

Group-specific outcomes of the running-induced fatigue protocol are presented in Table 2. Final heart rate, Borg score, and protocol duration were comparable across groups. Importantly, all participants achieved the primary fatigue criterion (Borg > 17), indicating that a similar level of running-induced fatigue was achieved before post-fatigue EMG assessment. The results revealed a significant main effect of fatigue for the ST (*p* = 0.042, ηp^2^ = 0.091, df = 1, F = 4.393) and GM (*p* < 0.001, ηp^2^ = 0.442, df = 1, F = 34.885) muscles during the early stance phase (Table 3). Pairwise comparisons of the estimated marginal means indicated that electromyography (EMG) amplitude significantly decreased following the fatigue protocol for both the ST (from 18.47%MVIC before fatigue to 14.80%MVIC after fatigue; mean difference = 3.67%MVIC, 95% CI 0.14 to 7.21, *p* = 0.042) and GM (from 19.23%MVIC before fatigue to 8.63%MVIC after fatigue; mean difference = 10.60%MVIC, 95% CI 6.98 to 14.22, *p* < 0.001) muscles. Significant main effects of group were also observed for the BF (*p* = 0.004, ηp^2^ = 0.258, df = 3, F = 5.092), ST (*p* = 0.002, ηp^2^ = 0.285, df = 3, F = 5.859), and GM (*p* = 0.013, ηp^2^ = 0.214, df = 3, F = 3.991) muscles during early stance. Pairwise comparisons showed that the SB-HT group exhibited a greater EMG amplitude in the BF muscle than the healthy group (*p* = 0.003, d = 1.05). Similarly, for the ST muscle, the allograft (*p* = 0.011, d = 1.05), SB-HT (*p* = 0.004, d = 1.02), and DB-HT (*p* = 0.011, d = 0.97) groups demonstrated higher EMG amplitude than the healthy group. For the GM muscle, the SB-HT group showed greater amplitude than the healthy group (*p* = 0.012, d = 0.90). No significant group-by-fatigue interactions were observed for any muscle during early stance (all *p* > 0.05; Table 3).

During the late stance phase (Table 4), the fatigue protocol did not produce significant main effects on EMG amplitude in any of the muscles examined (all *p* > 0.05). However, significant main effects of group were found for the TA (*p* = 0.003, ηp^2^ = 0.265, df = 3, F = 5.283), GC (*p* < 0.001, ηp^2^ = 0.294, df = 3, F = 1.490), RF (*p* = 0.021, ηp^2^ = 0.197, df = 3, F = 3.593), BF (*p* < 0.001, ηp^2^ = 0.333, df = 3, F = 5.262), ST (*p* < 0.001, ηp^2^ = 0.346, df = 3, F = 7.756), and GM (*p* = 0.029, ηp^2^ = 0.184, df = 3, F = 3.296) muscles. Pairwise comparisons revealed that TA EMG amplitude was greater in the allograft group than in the SB-HT (*p* = 0.005, d = 1.04), DB-HT (*p* = 0.019, d = 0.97), and healthy (*p* = 0.008, d = 1.08) groups. For the GC muscle, the allograft group exhibited higher amplitude than the healthy group (*p* = 0.043, d = 0.52). Likewise, RF amplitude was greater in the allograft group compared with the healthy group (*p* = 0.013, d = 1.03).

In contrast, BF amplitude was lower in the allograft group than in both the SB-HT (*p* = 0.003, d = 1.04) and DB-HT (*p* = 0.016, d = 0.91) groups. For the ST muscle, the SB-HT (*p* = 0.009, d = 0.93), DB-HT (*p* < 0.001, d = 1.28), and allograft (*p* = 0.002, d = 1.23) groups presented greater amplitude than the healthy group. Finally, GM amplitude was higher in the SB-HT group compared with the healthy group (*p* = 0.041, d = 0.78).

Regarding interaction effects, a significant group-by-fatigue interaction was identified for the VM muscle (*p* = 0.002, ηp^2^ = 0.279, df = 3, F = 5.672). Post hoc analyses indicated a significant reduction in EMG amplitude following fatigue in the DB-HT group (from 25.68 before fatigue to 14.93 after fatigue; mean difference = 10.75 %MVIC, 95% CI −4.13 to −17.37, *p* = 0.004). In contrast, no significant fatigue-related changes were observed in the other groups during the late stance phase (Table 4).

During running, overall stance time across the entire stance phase is presented in Table 5. The analysis showed no significant main effect of group (*p* = 0.269, ηp^2^ = 0.085, df = 3, F = 1.356) and no significant main effect of fatigue (*p* = 0.657, ηp^2^ = 0.005, df = 1, F = 0.200) for stance time. However, a significant group-by-fatigue interaction was observed (*p* = 0.044, ηp^2^ = 0.167, df = 3, F = 2.934).

## 4. Discussion

This study aimed to assess how a running fatigue protocol affects EMG amplitude in the lower-limb muscles of individuals with ACLR who underwent different graft types (SB-HT, DB-HT, and allograft), compared with healthy controls. The findings show that graft type was associated with distinct lower limb EMG activation patterns during running after ACLR. Fatigue-related group differences were limited, with significant group-by-fatigue interaction only seen for vastus medialis during late stance. While fatigue generally reduced ST and GM activity during the early stance phase, graft-specific neuromuscular adaptations were observed mainly during the late stance phase. Notably, allograft patients showed compensatory hyperactivation of the TA, GC, and RF, while the DB-HT group exhibited a distinct, fatigue-related decrease in VM activation. These results suggest that graft choice may be associated with different neuromuscular activation strategies during running under fatigued conditions.

One possible explanation for the higher EMG amplitudes observed in the allograft group during the late stance phase is that they may reflect a compensatory neuromuscular response related to altered sensory input following reconstruction. Our finding of increased activation in the allograft group, despite no apparent performance advantage, may indicate a different neuromuscular strategy during late stance. This pattern may represent an adaptive response to altered ligament-related sensory input; however, its functional implications remain unclear and should be interpreted cautiously, particularly because movement efficiency and joint loading were not directly assessed in the present study [38]. In addition, because graft processing characteristics were not documented in the present study, no conclusions can be drawn regarding their potential influence on the observed findings.

In contrast, the fatigue-related reduction in VM activation observed in the DB-HT group may indicate reduced quadriceps motor drive during fatigue. One possible explanation is that this pattern may be related to arthrogenic muscle inhibition (AMI). AMI is a reflex-driven protective response associated with altered joint afferent input and is often discussed in the context of post-surgical biomechanical and inflammatory changes [39]. While DB-HT techniques aim to restore normal joint movement, increased surgical complexity and tunnel density may influence the local joint environment [40]. However, this interpretation should be considered speculative, as AMI was not directly assessed in the present study, and related factors such as pain, effusion, corticospinal function, voluntary activation, and quadriceps torque were not measured. Therefore, the present findings should be interpreted as suggesting a possible mechanism rather than confirming AMI as the cause of the reduced VM activation. This pattern may suggest that DB-HT patients could exhibit greater fatigue-related reductions in quadriceps activation during late stance during demanding activities; however, this interpretation requires confirmation in future studies [41].

These results reinforce the use of fatigue as a “functional stress test” that unmasks latent neuromuscular deficits [42]. Conventional resting assessments often mask these impairments because they fail to challenge the central nervous system’s ability to coordinate muscle recruitment as peripheral capacity declines. The discrepancy between gross kinematic stability, often preserved in athletes, and fine-tuned neuromuscular control highlights a “control-performance dissociation.” Our data suggest that while reconstructed limbs may appear functionally adequate at rest, graft-dependent differences in muscle activation patterns may persist long after the RTS transition [43,44]. This emphasizes that graft-related factors may continue to influence neuromuscular control strategies into the later stages of rehabilitation.

The phase-specific findings are clinically relevant. Early stance showed a general reduction in ST and GM activation after fatigue, suggesting altered recruitment of the posterior chain and hip stabilizers during initial loading. In contrast, the late stance revealed more graft-related differences, including the DB-HT-specific VM response and the higher activation pattern in the allograft group. This supports the value of analyzing stance phases separately rather than relying on whole stance EMG averages.

Clinically, these findings suggest that late-stage rehabilitation and return-to-running assessment may need to consider graft-specific neuromuscular profiles. However, because the present study did not directly evaluate rehabilitation interventions or training responses, no specific clinical recommendations can be made. In addition, fatigue-focused functional assessments may be useful in RTS evaluation, as they could help identify graft-specific neuromuscular patterns that are not apparent during standard non-fatigue assessments. Overall, the clinical relevance of the present findings lies in highlighting potential graft-specific neuromuscular considerations warranting further investigation, rather than in establishing definitive rehabilitation recommendations.

This study has several limitations to consider when interpreting the results. First, surface EMG is limited to superficial muscles and may be affected by crosstalk and electrode placement variability, making it difficult to directly assess deep stabilizers such as the popliteus and potentially leading to an underestimation of the complexity of neuromuscular control in the knee. Second, although participants were matched on time since surgery and all assessments were performed after they received clearance to RTS, differences among individuals in graft healing, surgical technique details, and prior rehabilitation could still confound neuromuscular adaptations. In addition, the cross-sectional design limits causal inference, and the relatively small sample size in each group may reduce the generalizability of the findings. Third, the use of the 220–age formula to estimate maximum heart rate is a limitation, as it may not precisely account for individual physiological variability compared to a direct maximal oxygen uptake (VO_2_max) test. However, to mitigate this, we employed the Borg scale (>17) as a complementary subjective measure to confirm that participants had indeed reached a state of high-intensity fatigue. Nevertheless, some between-group variability in the actual level of fatigue reached cannot be completely ruled out. In addition, the absence of simultaneous kinematic and kinetic assessments limits the interpretation of the functional and biomechanical significance of the observed EMG changes. Furthermore, between-group comparisons were made between the reconstructed limb in the ACLR groups and the dominant limb in the healthy control group; this difference in limb selection should be considered when interpreting the findings. Further, using a treadmill for the fatigue protocol and overground running for the post-fatigue assessment may introduce additional variability because of the known biomechanical differences between these running conditions. However, this methodological approach was primarily dictated by equipment availability, as the biomechanical measurements could only be performed on the overground in our laboratory. Therefore, future studies should consider conducting both the fatigue protocol and biomechanical assessment in the same running environment. Finally, the study included only male participants, which limits the applicability of the results to females.

## 5. Conclusions

This study indicates that graft type is associated with distinct lower-limb EMG activation patterns during running after ACLR. Fatigue induced general reductions in semitendinosus and gluteus medius activation during early stance. Graft-specific fatigue effects were observed in the vastus medialis during late stance, with the DB-HT group showing reduced activation. The allograft group demonstrated higher activation of selected muscles during late stance, which may reflect different neuromuscular strategies for stability. While these results provide a potential framework for understanding fatigue-related neuromuscular alterations after ACLR, given the cross-sectional design and small subgroup sample sizes, these findings should be interpreted as preliminary and require confirmation in future longitudinal studies.

## Figures and Tables

**Table 1:** Demographic and anthropometric characteristics of participants.

Characteristics	SB-HT (N = 16)	DB-HT (N = 12)	Allograft (N = 10)	Healthy Group (N = 15)	*p*-Value
Age (years)	26.6 ± 0.4	26.9 ± 0.4	25.9 ± 0.6	26.5 ± 0.3	1.000
Height (cm)	179.5 ± 3.5	179.4 ± 6.5	180.3 ± 2.2	181.8 ± 3.2	0.316
Weight (kg)	79.8 ± 2.0	82.3 ± 1.1	80.7 ± 1.1	81.0 ± 2.2	0.215
Body mass index (kg/m^2^)	22.3 ± 2.0	22.4 ± 1.1	24.0 ± 2.0	22.8 ± 3.0	0.256
Time post-ACLR surgery (months)	10.8 ± 0.8	10.6 ± 1.0	11.6 ± 1.0	-	0.124
Injury–surgery interval (months)	7.5 ± 0.3	8.2 ± 1.1	7.3 ± 1.1	-	0.210
Tegner activity score	6.3 ± 0.4	6.0 ± 0.6	6.2 ± 0.9	6.4 ± 0.7	0.219
Weekly walking/running (km)	7.6 ± 1.0	7.6 ± 0.5	7.9 ± 1.9	9.2 ± 3.1	0.125
Navicular drop (mm)	7.7 ± 1.8	7.4 ± 1.2	8.0 ± 1.5	7.3 ± 1.2	0.356
Foot posture index	3.3 ± 0.3	3.0 ± 0.5	3.2 ± 1.6	3.3 ± 0.2	0.100

**Group Designations: SB-HT:** single-bundle hamstring tendon, **DB-HT:** double-bundle hamstring tendon.

**Table 2:** Group-specific outcomes of the running-induced fatigue protocol.

Variable	SB-HT (N = 16)	DB-HT (N = 12)	Allograft (N = 10)	Healthy (N = 15)
Total protocol duration (min)	16.8 ± 2.1	16.4 ± 1.9	17.1 ± 2.3	17.5 ± 2.0
Final treadmill speed (km/h)	12.4 ± 1.0	12.2 ± 0.9	12.6 ± 1.1	12.8 ± 1.0
Final heart rate (beats/min)	176.3 ± 6.8	174.9 ± 7.1	177.1 ± 6.5	178.4 ± 5.9
Final heart rate (%HRmax)	88.6 ± 2.9	88.1 ± 3.1	89.0 ± 2.7	89.4 ± 2.5
Final Borg score	18.3 ± 0.5	18.2 ± 0.6	18.4 ± 0.5	18.5 ± 0.5
Localized fatigue (0–10)	7.9 ± 1.1	8.1 ± 1.0	7.8 ± 1.2	8.0 ± 1.0
Perceived knee instability/discomfort (0–10)	3.2 ± 1.4	3.6 ± 1.5	3.0 ± 1.3	0.8 ± 0.6
Met primary fatigue criterion (Borg > 17), n (%)	16 (100)	12 (100)	10 (100)	15 (100)
Met complementary HR criterion (≥85% HRmax), n (%)	15 (93.8)	11 (91.7)	10 (100)	15 (100)

**Group Designations: SB-HT:** single-bundle hamstring tendon, **DB-HT:** double-bundle hamstring tendon.

**Table 3:** (**A**) Descriptive EMG amplitude (%MVIC) during the early stance phase of running. (**B**) ANOVA results for EMG amplitude during the early stance phase.

A											
Muscle				Healthy Group		ACLR Group (Allograft)		ACLR Group (SB-HT)		ACLR Group (DB-HT)	
				Before Fatigue	After Fatigue	Before Fatigue	After Fatigue	Before Fatigue	After Fatigue	Before Fatigue	After Fatigue
Tibialis anterior				16.42 ± 10.72	19.84 ± 8.15	26.62 ± 8.44	22.18 ± 8.28	22.60 ± 9.02	24.41 ± 9.62	18.28 ± 3.68	19.68 ± 5.80
Gastrocnemius				7.89 ± 6.33	8.54 ± 5.50	6.79 ± 2.50	7.02 ± 4.17	10.19 ± 5.05	9.42 ± 6.77	11.98 ± 5.76	13.56 ± 7.51
Vastus lateralis				13.38 ± 10.82	18.57 ± 18.37	27.13 ± 22.04	18.64 ± 12.70	14.59 ± 10.79	13.56 ± 12.10	17.45 ± 13.21	15.03 ± 10.49
Rectus femoris				10.71 ± 7.88	12.13 ± 4.68	23.50 ± 18.44	22.36 ± 16.31	17.08 ± 12.57	15.27 ± 15.13	17.53 ± 13.77	11.77 ± 7.95
Vastus medialis				8.14 ± 2.44	13.87 ± 13.23	17.88 ± 12.23	16.53 ± 12.21	14.00 ± 12.83	12.88 ± 7.50	15.36 ± 8.71	12.22 ± 7.85
Biceps femoris				6.97 ± 3.78	6.94 ± 2.28	17.87 ± 13.10	19.23 ± 17.03	19.11 ± 8.58	22.51 ± 16.28	13.63 ± 5.85	16.51 ± 6.81
Semitendinosus				8.47 ± 6.92	6.26 ± 2.87	22.29 ± 10.74	17.94 ± 10.98	21.17 ± 13.06	18.40 ± 8.96	21.92 ± 15.78	16.57 ± 7.52
Gluteus medius				10.71 ± 4.96	5.82 ± 4.23	24.72 ± 12.95	8.16 ± 4.17	23.22 ± 13.31	11.09 ± 6.57	18.25 ± 13.36	9.44 ± 5.22
**B**											
**Muscle**			**Main Effect of Fatigue**			**Main Effect of Group**			**Group-by-Fatigue Interaction**		
Tibialis anterior			F(1,49) = 0.246, *p* = 0.632, ηp^2^ = 0.005			F(3,49) = 2.333, *p* = 0.116, ηp^2^ = 0.125			F(3,49) = 2.164, *p* = 0.137, ηp^2^ = 0.117		
Gastrocnemius			F(1,49) = 0.395, *p* = 0.566, ηp^2^ = 0.008			F(3,49) = 2.815, *p* = 0.070, ηp^2^ = 0.147			F(3,49) = 0.610, *p* = 0.656, ηp^2^ = 0.036		
Vastus lateralis			F(1,49) = 0.545, *p* = 0.498, ηp^2^ = 0.011			F(3,49) = 1.420, *p* = 0.294, ηp^2^ = 0.080			F(3,49) = 1.248, *p* = 0.350, ηp^2^ = 0.071		
Rectus femoris			F(1,49) = 1.989, *p* = 0.186, ηp^2^ = 0.039			F(3,49) = 1.795, *p* = 0.199, ηp^2^ = 0.099			F(3,49) = 1.343, *p* = 0.319, ηp^2^ = 0.076		
Vastus medialis			F(1,49) = 0.000, *p* = 0.979, ηp^2^ = 0.000			F(3,49) = 0.842, *p* = 0.525, ηp^2^ = 0.049			F(3,49) = 2.659, *p* = 0.082, ηp^2^ = 0.140		
Biceps femoris			F(1,49) = 1.883, *p* = 0.200, ηp^2^ = 0.037			F(3,49) = 5.679, *p* = 0.004 *, ηp^2^ = 0.258			F(3,49) = 0.333, *p* = 0.823, ηp^2^ = 0.020		
Semitendinosus			F(1,49) = 4.905, *p* = 0.042, ηp^2^ = 0.091			F(3,49) = 6.510, *p* = 0.002 *, ηp^2^ = 0.285			F(3,49) = 0.198, *p* = 0.913, ηp^2^ = 0.012		
Gluteus medius			F(1,49) = 38.814, *p* < 0.001 *, ηp^2^ = 0.442			F(3,49) = 4.447, *p* = 0.013 *, ηp^2^ = 0.214			F(3,49) = 1.896, *p* = 0.181, ηp^2^ = 0.104		

*: Significance level: *p* < 0.05; ηp^2^: partial eta squared. Statistical test: Two-way ANOVA with repeated measures.

**Table 4:** (**A**) Descriptive EMG amplitude (%MVIC) during the late stance phase of running. (**B**) ANOVA results for EMG amplitude during the late stance phase.

A											
Muscle				Healthy Group		ACLR Group (Allograft)		ACLR Group (SB-HT)		ACLR Group (DB-HT)	
				Before Fatigue	Before Fatigue	Before Fatigue	After Fatigue	Before Fatigue	After Fatigue	Before Fatigue	After Fatigue
Tibialis anterior				6.21 ± 2.17	6.98 ± 2.44	12.69 ± 7.86	10.24 ± 5.66	6.71 ± 3.23	6.81 ± 2.30	6.88 ± 2.46	7.30 ± 3.03
Gastrocnemius				28.02 ± 8.44	29.01 ± 6.42	35.43 ± 8.48	36.32 ± 12.01	36.41 ± 13.85	35.09 ± 12.19	35.13 ± 14.58	36.10 ± 11.37
Vastus lateralis				17.78 ± 9.09	22.37 ± 13.32	30.65 ± 18.57	34.16 ± 15.16	24.86 ± 12.38	24.05 ± 11.93	30.73 ± 12.19	22.44 ± 12.34
Rectus femoris				12.55 ± 7.99	16.02 ± 10.82	29.92 ± 17.40	38.80 ± 27.88	24.10 ± 17.78	22.37 ± 13.48	28.24 ± 14.23	21.30 ± 12.08
Vastus medialis				11.01 ± 3.40	14.89 ± 9.10	19.64 ± 9.56	20.02 ± 9.95	19.37 ± 18.63	19.25 ± 14.07	25.68 ± 12.64	14.92 ± 6.08
Biceps femoris				5.25 ± 3.98	5.07 ± 3.33	16.06 ± 11.43	17.99 ± 16.48	18.37 ± 8.42	18.85 ± 9.67	20.19 ± 9.20	20.06 ± 10.89
Semitendinosus				5.39 ± 5.00	4.63 ± 4.36	15.79 ± 7.98	15.24 ± 8.07	13.95 ± 9.19	12.06 ± 6.55	18.72 ± 10.62	13.19 ± 5.27
Gluteus medius				12.27 ± 5.11	16.63 ± 10.18	22.70 ± 15.06	25.56 ± 10.22	26.34 ± 18.06	27.05 ± 16.22	20.76 ± 10.52	14.77 ± 7.71
**B**											
**Muscle**			**Main Effect of Fatigue**			**Main Effect of Group**			**Group-by-Fatigue Interaction**		
Tibialis anterior			F(1,49) = 0.246, *p* = 0.635, ηp^2^ = 0.005			F(3,49) = 5.889, *p* = 0.003 *, ηp^2^ = 0.265			F(3,49) = 1.343, *p* = 0.320, ηp^2^ = 0.076		
Gastrocnemius			F(1,49) = 0.049, *p* = 0.826, ηp^2^ = 0.001			F(3,49) = 6.802, *p* < 0.001 *, ηp^2^ = 0.294			F(3,49) = 0.148, *p* = 0.938, ηp^2^ = 0.009		
Vastus lateralis			F(1,49) = 0.000, *p* = 0.901, ηp^2^ = 0.000			F(3,49) = 2.355, *p* = 0.111, ηp^2^ = 0.126			F(3,49) = 2.312, *p* = 0.118, ηp^2^ = 0.124		
Rectus femoris			F(1,49) = 0.197, *p* = 0.690, ηp^2^ = 0.004			F(3,49) = 4.007, *p* = 0.021 *, ηp^2^ = 0.197			F(3,49) = 2.248, *p* = 0.124, ηp^2^ = 0.121		
Vastus medialis			F(1,49) = 1.725, *p* = 0.223, ηp^2^ = 0.034			F(3,49) = 1.192, *p* = 0.373, ηp^2^ = 0.068			F(3,49) = 6.320, *p* = 0.002 *, ηp^2^ = 0.279		
Biceps femoris			F(1,49) = 0.197, *p* = 0.692, ηp^2^ = 0.004			F(3,49) = 8.154, *p* < 0.001 *, ηp^2^ = 0.333			F(3,49) = 0.132, *p* = 0.949, ηp^2^ = 0.008		
Semitendinosus			F(1,49) = 3.072, *p* = 0.103, ηp^2^ = 0.059			F(3,49) = 8.641, *p* < 0.001 *, ηp^2^ = 0.346			F(3,49) = 0.860, *p* = 0.517, ηp^2^ = 0.050		
Gluteus medius			F(1,49) = 0.049, *p* = 0.806, ηp^2^ = 0.001			F(3,49) = 3.683, *p* = 0.029 *, ηp^2^ = 0.184			F(3,49) = 1.478, *p* = 0.277, ηp^2^ = 0.083		

*: Significance level: *p* < 0.05; ηp^2^: partial eta squared. Statistical test: Two-way ANOVA with repeated measures.

**Table 5:** Overall stance time (ms) during running.

Group	Before Fatigue (ms)	After Fatigue (ms)
ACLR (DB-HT)	328.41 ± 42.93	338.61 ± 57.60
ACLR (SB-HT)	338.64 ± 40.07	319.35 ± 40.36
ACLR (Allograft)	352.51 ± 61.81	366.55 ± 58.24
Healthy	321.18 ± 32.92	324.72 ± 32.92
**Statistical Effects**	***p*-value**	**ηp^2^**
Main effect of Fatigue	0.657	0.005
Main effect of Group	0.269	0.085
Group-by-Fatigue Interaction	0.044 *	0.167

Note: Data are mean ± SD. Statistical test: Two-way repeated measures ANOVA. *: Significant difference (*p* < 0.05). ηp^2^: partial eta squared.

## Data Availability

The complete datasets generated and analyzed during the present study, including de-identified individual-level raw EMG data, processed muscle activation variables, and demographic characteristics, are fully available from the corresponding author upon reasonable request. No ethical restrictions or privacy limitations prevent the sharing of these data, and they will be made available to researchers wishing to replicate or build upon our findings.

## Supplementary Materials

The following supporting information can be downloaded at: https://www.mdpi.com/article/10.3390/s26154904/s1, File S1: Details of the MVIC normalization procedure, including participant position, stabilization, and equipment setup.

## Abbreviations

The following abbreviations are used in this manuscript:

ACLR	Anterior cruciate ligament reconstruction
SB-HT	Single-bundle hamstring tendon
DB-HT	Double-bundle hamstring tendon
RTS	Return to sport
ROM	Range of motion
HT	Hamstring
EMG	Electromyography
