# Supplementary material for: Graft-Specific Neuromuscular Responses and EMG Activities During Running Before and After Running-Induced Fatigue in Individuals with ACL Reconstruction
Source: Sensors (Basel). 2026 Aug 3;26(15):4904. doi: 10.3390/s26154904 (PMC13469413; doi:10.3390/s26154904)
Supplement: Supplementary file 1 [file sensors-26-04904-s001.zip › sensors-4413330-Supplementary File S1.pdf]

Section 1: Early Stance Phase - EMG Amplitude Outputs

Tibialis Anterior (TA) - Early Stance

```
GLM NtaLRpre NtaLRpos BY groups
  /WSFACTOR=fatigue 2 Polynomial
  /METHOD=SSTYPE(3)
  /POSTHOC=groups(TUKEY)
  /EMMEANS=TABLES(OVERALL)
  /EMMEANS=TABLES(groups) COMPARE ADJ(BONFERRONI)
  /EMMEANS=TABLES(fatigue) COMPARE ADJ(BONFERRONI)
  /EMMEANS=TABLES(groups*fatigue)
  /PRINT=DESCRIPTIVE ETASQ OPOWER PARAMETER HOMOGENEITY
  /CRITERIA=ALPHA(.05)
  /WSDESIGN=fatigue
  /DESIGN=groups.
```

General Linear Model

Notes

Output Created		17-MAY-2026 21:25:48
Comments		
Input	Data	C:\Users\RaSa\Desktop\پروپوزال\New folder\data\emgmi1.sav
	Active Dataset	DataSet2
	Filter	<none>
	Weight	<none>
	Split File	<none>
	N of Rows in Working Data File	48
Missing Value Handling	Definition of Missing	User-defined missing values are treated as missing.
	Cases Used	Statistics are based on all cases with valid data for all variables in the model.

Syntax		GLM NtaLRpre NtaLRpos BY groups<br>/WSFACTOR=fatigue 2 Polynomial<br>/METHOD=SSTYPE(3)<br>/POSTHOC=groups(TUKEY)<br>/EMMEANS=TABLES(OVERALL)<br>/EMMEANS=TABLES(groups) COMPARE ADJ(BONFERRONI)<br>/EMMEANS=TABLES(fatigue) COMPARE ADJ(BONFERRONI)<br>/EMMEANS=TABLES(groups*fatigue)<br>/PRINT=DESCRIPTIVE ETASQ OPOWER PARAMETER HOMOGENEITY<br>/CRITERIA=ALPHA(.05)<br>/WSDESIGN=fatigue<br>/DESIGN=groups.
Resources	Processor Time	00:00:00.03
	Elapsed Time	00:00:00.03

Within-Subjects Factors

Measure: MEASURE_1

fatigue	Dependent Variable
1	NtaLRpre
2	NtaLRpos

Between-Subjects Factors

		N
groups	1.00	10
	2.00	16
	3.00	12
	4.00	15

Descriptive Statistics

	groups	Mean	Std. Deviation	N
NtaLRpre	1.00	26.6284	8.44212	10
	2.00	22.6057	9.02421	16
	3.00	18.2886	3.68325	12
	4.00	16.4299	10.72058	15
	Total	20.8654	8.91293	53

NtaLRpos	1.00	22.1887	8.28831	10
	2.00	24.4141	9.62103	16
	3.00	19.6898	5.80578	12
	4.00	19.8408	8.15645	15
	Total	21.7677	8.22605	53

Box's Test of Equality of Covariance Matrices[a]

Box's M	20.717
F	2.101
df1	9
df2	12234.839
Sig.	.026

Tests the null hypothesis that the observed covariance matrices of the dependent variables are equal across groups.
a. Design: Intercept + groups
Within Subjects Design: fatigue

Multivariate Tests[a]

Effect		Value	F	Hypothesis df	Error df	Sig.	Partial Eta Squared	Noncent. Parameter	Observed Power[c]
fatigue	Pillai's Trace	.005	.232[b]	1.000	44.000	.632	.005	.232	.076
	Wilks' Lambda	.995	.232[b]	1.000	44.000	.632	.005	.232	.076
	Hotelling's Trace	.005	.232[b]	1.000	44.000	.632	.005	.232	.076
	Roy's Largest Root	.005	.232[b]	1.000	44.000	.632	.005	.232	.076
fatigue * groups	Pillai's Trace	.117	1.938[b]	3.000	44.000	.137	.117	5.815	.466
	Wilks' Lambda	.883	1.938[b]	3.000	44.000	.137	.117	5.815	.466
	Hotelling's Trace	.132	1.938[b]	3.000	44.000	.137	.117	5.815	.466
	Roy's Largest Root	.132	1.938[b]	3.000	44.000	.137	.117	5.815	.466

a. Design: Intercept + groups
Within Subjects Design: fatigue
b. Exact statistic
c. Computed using alpha = .05

Mauchly's Test of Sphericity[a]

Measure: MEASURE_1

Within Subjects Effect	Mauchly's W	Approx. Chi-Square	df	Sig.	Epsilon[b] Greenhouse-Geisser	Huynh-Feldt	Lower-bound
fatigue	1.000	.000	0	.	1.000	1.000	1.000

Tests the null hypothesis that the error covariance matrix of the orthonormalized transformed dependent variables is proportional to an identity matrix.
a. Design: Intercept + groups
Within Subjects Design: fatigue

b. May be used to adjust the degrees of freedom for the averaged tests of significance. Corrected tests are displayed in the Tests of Within-Subjects Effects table.

Tests of Within-Subjects Effects

Measure: MEASURE_1

Source		Type III Sum of Squares	df	Mean Square	F	Sig.	Partial Eta Squared	Noncent. Parameter	Observed Power[a]
fatigue	Sphericity Assumed	6.835	1	6.835	.232	.632	.005	.232	.076
	Greenhouse-Geisser	6.835	1.000	6.835	.232	.632	.005	.232	.076
	Huynh-Feldt	6.835	1.000	6.835	.232	.632	.005	.232	.076
	Lower-bound	6.835	1.000	6.835	.232	.632	.005	.232	.076
fatigue * groups	Sphericity Assumed	171.089	3	57.030	1.938	.137	.117	5.815	.466
	Greenhouse-Geisser	171.089	3.000	57.030	1.938	.137	.117	5.815	.466
	Huynh-Feldt	171.089	3.000	57.030	1.938	.137	.117	5.815	.466
	Lower-bound	171.089	3.000	57.030	1.938	.137	.117	5.815	.466
Error(fatigue)	Sphericity Assumed	1294.606	44	29.423					
	Greenhouse-Geisser	1294.606	44.000	29.423					
	Huynh-Feldt	1294.606	44.000	29.423					
	Lower-bound	1294.606	44.000	29.423					

a. Computed using alpha = .05

Tests of Within-Subjects Contrasts

Measure: MEASURE_1

Source	fatigue	Type III Sum of Squares	df	Mean Square	F	Sig.	Partial Eta Squared	Noncent. Parameter	Observed Power[a]
fatigue	Linear	6.835	1	6.835	.232	.632	.005	.232	.076
fatigue * groups	Linear	171.089	3	57.030	1.938	.137	.117	5.815	.466
Error(fatigue)	Linear	1294.606	44	29.423					

a. Computed using alpha = .05

Levene's Test of Equality of Error Variances[a]

		Levene Statistic	df1	df2	Sig.
NtaLRpre	Based on Mean	3.189	3	44	.033
	Based on Median	1.532	3	44	.220
	Based on Median and with adjusted df	1.532	3	25.584	.230
	Based on trimmed mean	3.019	3	44	.040
NtaLRpos	Based on Mean	.841	3	44	.479
	Based on Median	.773	3	44	.515
	Based on Median and with adjusted df	.773	3	37.329	.516
	Based on trimmed mean	.842	3	44	.478

Tests the null hypothesis that the error variance of the dependent variable is equal across groups.
a. Design: Intercept + groups
Within Subjects Design: fatigue

Tests of Between-Subjects Effects

Measure: MEASURE_1
Transformed Variable: Average

Source	Type III Sum of Squares	df	Mean Square	F	Sig.	Partial Eta Squared	Noncent. Parameter	Observed Power[a]
Intercept	41582.488	1	41582.488	383.593	.000	.897	383.593	1.000
groups	678.676	3	226.225	2.087	.116	.125	6.261	.498
Error	4769.717	44	108.403					

a. Computed using alpha = .05

Parameter Estimates

Dependent Variable	Parameter	B	Std. Error	t	Sig.	95% Confidence Interval		Partial Eta Squared	Noncent. Parameter	Observed Power[b]
						Lower Bound	Upper Bound			
NtaLRpre	Intercept	16.430	2.527	6.502	.000	11.337	21.522	.490	6.502	1.000
	[groups=1.00]	10.199	3.767	2.708	.010	2.607	17.790	.143	2.708	.754
	[groups=2.00]	6.176	3.282	1.881	.067	-.439	12.791	.074	1.881	.452
	[groups=3.00]	1.859	3.498	.531	.598	-5.192	8.909	.006	.531	.081
	[groups=4.00]	0[a]	.	.	.	.	.	.	.	.
NtaLRpos	Intercept	19.841	2.479	8.004	.000	14.845	24.837	.593	8.004	1.000
	[groups=1.00]	2.348	3.695	.635	.528	-5.099	9.795	.009	.635	.095
	[groups=2.00]	4.573	3.220	1.420	.163	-1.917	11.063	.044	1.420	.285
	[groups=3.00]	-.151	3.432	-.044	.965	-7.067	6.765	.000	.044	.050
	[groups=4.00]	0[a]	.	.	.	.	.	.	.	.

a. This parameter is set to zero because it is redundant.
b. Computed using alpha = .05

Estimated Marginal Means

1. Grand Mean

Measure: MEASURE_1

Mean	Std. Error	95% Confidence Interval	
		Lower Bound	Upper Bound
21.261	1.086	19.073	23.448

2. groups

Estimates

Measure: MEASURE_1

groups	Mean	Std. Error	95% Confidence Interval	
			Lower Bound	Upper Bound

1.00	24.409	2.454	19.463	29.354
2.00	23.510	1.841	19.801	27.219
3.00	18.989	2.125	14.706	23.272
4.00	18.135	2.220	13.662	22.609

Pairwise Comparisons

Measure: MEASURE_1

(I) groups	(J) groups	Mean Difference (I-J)	Std. Error	Sig.[a]	95% Confidence Interval for Difference[a]	
					Lower Bound	Upper Bound
1.00	2.00	.899	3.068	1.000	-7.576	9.374
	3.00	5.419	3.246	.613	-3.550	14.389
	4.00	6.273	3.309	.387	-2.869	15.416
2.00	1.00	-.899	3.068	1.000	-9.374	7.576
	3.00	4.521	2.811	.690	-3.247	12.288
	4.00	5.375	2.884	.414	-2.592	13.341
3.00	1.00	-5.419	3.246	.613	-14.389	3.550
	2.00	-4.521	2.811	.690	-12.288	3.247
	4.00	.854	3.073	1.000	-7.637	9.344
4.00	1.00	-6.273	3.309	.387	-15.416	2.869
	2.00	-5.375	2.884	.414	-13.341	2.592
	3.00	-.854	3.073	1.000	-9.344	7.637

Based on estimated marginal means

a. Adjustment for multiple comparisons: Bonferroni.

Univariate Tests

Measure: MEASURE_1

	Sum of Squares	df	Mean Square	F	Sig.	Partial Eta Squared	Noncent. Parameter	Observed Power[a]
Contrast	339.338	3	113.113	2.087	.116	.125	6.261	.498
Error	2384.859	44	54.201					

The F tests the effect of groups. This test is based on the linearly independent pairwise comparisons among the estimated marginal means.

a. Computed using alpha = .05

3. fatigue

Estimates

Measure: MEASURE_1

fatigue	Mean	Std. Error	95% Confidence Interval	
			Lower Bound	Upper Bound
1	20.988	1.236	18.498	23.479
2	21.533	1.212	19.090	23.976

Pairwise Comparisons

Measure: MEASURE_1

(I) fatigue	(J) fatigue	Mean Difference (I-J)	Std. Error	Sig.[a]	95% Confidence Interval for Difference[a]	
					Lower Bound	Upper Bound
1	2	-.545	1.131	.632	-2.825	1.734
2	1	.545	1.131	.632	-1.734	2.825

Based on estimated marginal means

a. Adjustment for multiple comparisons: Bonferroni.

Multivariate Tests

	Value	F	Hypothesis df	Error df	Sig.	Partial Eta Squared	Noncent. Parameter	Observed Power[b]
Pillai's trace	.005	.232[a]	1.000	44.000	.632	.005	.232	.076
Wilks' lambda	.995	.232[a]	1.000	44.000	.632	.005	.232	.076
Hotelling's trace	.005	.232[a]	1.000	44.000	.632	.005	.232	.076
Roy's largest root	.005	.232[a]	1.000	44.000	.632	.005	.232	.076

Each F tests the multivariate effect of fatigue. These tests are based on the linearly independent pairwise comparisons among the estimated marginal means.

a. Exact statistic

b. Computed using alpha = .05

4. groups * fatigue

Measure: MEASURE_1

groups	fatigue	Mean	Std. Error	95% Confidence Interval	
				Lower Bound	Upper Bound
1.00	1	26.628	2.793	20.999	32.258
	2	22.189	2.740	16.666	27.712
2.00	1	22.606	2.095	18.383	26.828
	2	24.414	2.055	20.272	28.556
3.00	1	18.289	2.419	13.413	23.164
	2	19.690	2.373	14.907	24.473
4.00	1	16.430	2.527	11.337	21.522
	2	19.841	2.479	14.845	24.837

Post Hoc Tests

groups

Multiple Comparisons

Measure: MEASURE_1

Tukey HSD

(I) groups	(J) groups	Mean Difference (I-J)	Std. Error	Sig.	95% Confidence Interval	
					Lower Bound	Upper Bound
1.00	2.00	.8987	3.06756	.991	-7.2917	9.0891
	3.00	5.4194	3.24641	.352	-3.2485	14.0873
	4.00	6.2732	3.30904	.245	-2.5619	15.1084
2.00	1.00	-.8987	3.06756	.991	-9.0891	7.2917
	3.00	4.5207	2.81147	.385	-2.9859	12.0273

	4.00	5.3745	2.88357	.258	-2.3246	13.0737
3.00	1.00	-5.4194	3.24641	.352	-14.0873	3.2485
	2.00	-4.5207	2.81147	.385	-12.0273	2.9859
	4.00	.8538	3.07314	.992	-7.3515	9.0591
4.00	1.00	-6.2732	3.30904	.245	-15.1084	2.5619
	2.00	-5.3745	2.88357	.258	-13.0737	2.3246
	3.00	-.8538	3.07314	.992	-9.0591	7.3515

Based on observed means.
The error term is Mean Square(Error) = 54.201.

Homogeneous Subsets

MEASURE_1

Tukey HSD[a,b,c]

groups	N	Subset<br>1
4.00	15	18.1353
3.00	12	18.9892
2.00	16	23.5099
1.00	10	24.4086
Sig.		.188

Means for groups in homogeneous subsets are displayed.
Based on observed means.
The error term is Mean Square(Error) = 54.201.
a. Uses Harmonic Mean Sample Size = 11.499.
b. The group sizes are unequal. The harmonic mean of the group sizes is used. Type I error levels are not guaranteed.
c. Alpha = .05.

```
GLM NgasLRpre NgasLRpos BY groups
  /WSFACTOR=fatigue 2 Polynomial
  /METHOD=SSTYPE(3)
  /POSTHOC=groups(TUKEY)
  /EMMEANS=TABLES(OVERALL)
  /EMMEANS=TABLES(groups) COMPARE ADJ(BONFERRONI)
  /EMMEANS=TABLES(fatigue) COMPARE ADJ(BONFERRONI)
  /EMMEANS=TABLES(groups*fatigue)
  /PRINT=DESCRIPTIVE ETASQ OPOWER PARAMETER HOMOGENEITY
  /CRITERIA=ALPHA(.05)
  /WSDESIGN=fatigue
  /DESIGN=groups.
```

General Linear Model

Notes

Output Created		17-MAY-2026 21:26:21
Comments		
Input	Data	C:\Users\RaSa\Desktop\پروپوزال\New folder\data\emgmi1.sav
	Active Dataset	DataSet2
	Filter	<none>
	Weight	<none>
	Split File	<none>
	N of Rows in Working Data File	48
Missing Value Handling	Definition of Missing	User-defined missing values are treated as missing.
	Cases Used	Statistics are based on all cases with valid data for all variables in the model.

Syntax		GLM NgasLRpre NgasLRpos BY groups<br>/WSFACTOR=fatigue 2 Polynomial<br>/METHOD=SSTYPE(3)<br>/POSTHOC=groups(TUKEY)<br>/EMMEANS=TABLES(OVERALL)<br>/EMMEANS=TABLES(groups) COMPARE ADJ(BONFERRONI)<br>/EMMEANS=TABLES(fatigue) COMPARE ADJ(BONFERRONI)<br>/EMMEANS=TABLES(groups*fatigue)<br>/PRINT=DESCRIPTIVE ETASQ OPOWER PARAMETER HOMOGENEITY<br>/CRITERIA=ALPHA(.05)<br>/WSDESIGN=fatigue<br>/DESIGN=groups.
Resources	Processor Time	00:00:00.02
	Elapsed Time	00:00:00.02

Within-Subjects Factors

Measure: MEASURE_1

fatigue	Dependent Variable
1	NgasLRpre
2	NgasLRpos

Between-Subjects Factors

		N
groups	1.00	10
	2.00	16
	3.00	12
	4.00	15

Descriptive Statistics

	groups	Mean	Std. Deviation	N
NgasLRpre	1.00	6.7961	2.50869	10
	2.00	10.1974	5.05291	16
	3.00	11.9859	5.76575	12
	4.00	7.8996	6.33578	15
	Total	9.4802	5.41143	53
NgasLRpos	1.00	7.0246	4.17547	10
	2.00	9.4216	6.77956	16
	3.00	13.5692	7.51355	12
	4.00	8.5489	5.50525	15
	Total	9.8091	6.54329	53

Box's Test of Equality of Covariance Matrices[a]

Box's M	12.359
F	1.253
df1	9
df2	12234.839
Sig.	.257

Tests the null hypothesis that the observed covariance matrices of the dependent variables are equal across groups.

a. Design: Intercept + groups
Within Subjects Design: fatigue

Multivariate Tests[a]

Effect		Value	F	Hypothesis df	Error df	Sig.	Partial Eta Squared	Noncent. Parameter	Observed Power[c]
fatigue	Pillai's Trace	.008	.335[b]	1.000	44.000	.566	.008	.335	.087
	Wilks' Lambda	.992	.335[b]	1.000	44.000	.566	.008	.335	.087
	Hotelling's Trace	.008	.335[b]	1.000	44.000	.566	.008	.335	.087
	Roy's Largest Root	.008	.335[b]	1.000	44.000	.566	.008	.335	.087
fatigue * groups	Pillai's Trace	.036	.542[b]	3.000	44.000	.656	.036	1.625	.152
	Wilks' Lambda	.964	.542[b]	3.000	44.000	.656	.036	1.625	.152
	Hotelling's Trace	.037	.542[b]	3.000	44.000	.656	.036	1.625	.152

Roy's Largest Root	.037	.542[b]	3.000	44.000	.656	.036	1.625	.152

a. Design: Intercept + groups
Within Subjects Design: fatigue
b. Exact statistic
c. Computed using alpha = .05

Mauchly's Test of Sphericity[a]

Measure: MEASURE_1

Within Subjects Effect	Mauchly's W	Approx. Chi-Square	df	Sig.	Epsilon[b]		
					Greenhouse-Geisser	Huynh-Feldt	Lower-bound
fatigue	1.000	.000	0	.	1.000	1.000	1.000

Tests the null hypothesis that the error covariance matrix of the orthonormalized transformed dependent variables is proportional to an identity matrix.
a. Design: Intercept + groups
Within Subjects Design: fatigue
b. May be used to adjust the degrees of freedom for the averaged tests of significance. Corrected tests are displayed in the Tests of Within-Subjects Effects table.

Tests of Within-Subjects Effects

Measure: MEASURE_1

Source		Type III Sum of Squares	df	Mean Square	F	Sig.	Partial Eta Squared	Noncent. Parameter	Observed Power[a]
fatigue	Sphericity Assumed	4.083	1	4.083	.335	.566	.008	.335	.087
	Greenhouse-Geisser	4.083	1.000	4.083	.335	.566	.008	.335	.087
	Huynh-Feldt	4.083	1.000	4.083	.335	.566	.008	.335	.087
	Lower-bound	4.083	1.000	4.083	.335	.566	.008	.335	.087
fatigue * groups	Sphericity Assumed	19.814	3	6.605	.542	.656	.036	1.625	.152
	Greenhouse-Geisser	19.814	3.000	6.605	.542	.656	.036	1.625	.152
	Huynh-Feldt	19.814	3.000	6.605	.542	.656	.036	1.625	.152
	Lower-bound	19.814	3.000	6.605	.542	.656	.036	1.625	.152
Error(fatigue)	Sphericity Assumed	536.638	44	12.196					
	Greenhouse-Geisser	536.638	44.000	12.196					
	Huynh-Feldt	536.638	44.000	12.196					
	Lower-bound	536.638	44.000	12.196					

a. Computed using alpha = .05

Tests of Within-Subjects Contrasts

Measure: MEASURE_1

Source	fatigue	Type III Sum of Squares	df	Mean Square	F	Sig.	Partial Eta Squared	Noncent. Parameter	Observed Power[a]
fatigue	Linear	4.083	1	4.083	.335	.566	.008	.335	.087
fatigue * groups	Linear	19.814	3	6.605	.542	.656	.036	1.625	.152
Error(fatigue)	Linear	536.638	44	12.196					

a. Computed using alpha = .05

Levene's Test of Equality of Error Variances[a]

		Levene Statistic	df1	df2	Sig.
NgasLRpre	Based on Mean	.984	3	44	.409
	Based on Median	.832	3	44	.483
	Based on Median and with adjusted df	.832	3	28.303	.487
	Based on trimmed mean	.894	3	44	.452
NgasLRpos	Based on Mean	1.863	3	44	.150
	Based on Median	1.014	3	44	.396
	Based on Median and with adjusted df	1.014	3	41.417	.396
	Based on trimmed mean	1.643	3	44	.193

Tests the null hypothesis that the error variance of the dependent variable is equal across groups.

a. Design: Intercept + groups
Within Subjects Design: fatigue

Tests of Between-Subjects Effects

Measure: MEASURE_1
Transformed Variable: Average

Source	Type III Sum of Squares	df	Mean Square	F	Sig.	Partial Eta Squared	Noncent. Parameter	Observed Power[a]
Intercept	8181.151	1	8181.151	148.947	.000	.772	148.947	1.000
groups	415.393	3	138.464	2.521	.070	.147	7.563	.584
Error	2416.769	44	54.927					

a. Computed using alpha = .05

Parameter Estimates

Dependent Variable	Parameter	B	Std. Error	t	Sig.	95% Confidence Interval Lower Bound	95% Confidence Interval Upper Bound	Partial Eta Squared	Noncent. Parameter	Observed Power[b]
NgasLRpre	Intercept	7.900	1.575	5.016	.000	4.726	11.074	.364	5.016	.998
	[groups=1.00]	-1.103	2.348	-.470	.641	-5.835	3.628	.005	.470	.075
	[groups=2.00]	2.298	2.046	1.123	.267	-1.825	6.421	.028	1.123	.196
	[groups=3.00]	4.086	2.180	1.874	.068	-.308	8.480	.074	1.874	.450
	[groups=4.00]	0[a]	.	.	.	.	.	.	.	.
NgasLRpos	Intercept	8.549	1.903	4.492	.000	4.713	12.384	.314	4.492	.992
	[groups=1.00]	-1.524	2.837	-.537	.594	-7.242	4.193	.007	.537	.082
	[groups=2.00]	.873	2.472	.353	.726	-4.110	5.855	.003	.353	.064
	[groups=3.00]	5.020	2.635	1.905	.063	-.290	10.330	.076	1.905	.462
	[groups=4.00]	0[a]	.	.	.	.	.	.	.	.

a. This parameter is set to zero because it is redundant.
b. Computed using alpha = .05

Estimated Marginal Means

1. Grand Mean

Measure: MEASURE_1

Mean	Std. Error	95% Confidence Interval Lower Bound	95% Confidence Interval Upper Bound
9.430	.773	7.873	10.988

2. groups

Estimates

Measure: MEASURE_1

groups	Mean	Std. Error	95% Confidence Interval

			Lower Bound	Upper Bound
1.00	6.910	1.747	3.390	10.431
2.00	9.809	1.310	7.169	12.450
3.00	12.778	1.513	9.729	15.826
4.00	8.224	1.580	5.040	11.409

Pairwise Comparisons

Measure: MEASURE_1

(I) groups	(J) groups	Mean Difference (I-J)	Std. Error	Sig.[a]	95% Confidence Interval for Difference[a] Lower Bound	Upper Bound
1.00	2.00	-2.899	2.184	1.000	-8.932	3.134
	3.00	-5.867	2.311	.088	-12.252	.517
	4.00	-1.314	2.355	1.000	-7.822	5.194
2.00	1.00	2.899	2.184	1.000	-3.134	8.932
	3.00	-2.968	2.001	.871	-8.497	2.561
	4.00	1.585	2.053	1.000	-4.086	7.256
3.00	1.00	5.867	2.311	.088	-.517	12.252
	2.00	2.968	2.001	.871	-2.561	8.497
	4.00	4.553	2.188	.259	-1.490	10.597
4.00	1.00	1.314	2.355	1.000	-5.194	7.822
	2.00	-1.585	2.053	1.000	-7.256	4.086
	3.00	-4.553	2.188	.259	-10.597	1.490

Based on estimated marginal means

a. Adjustment for multiple comparisons: Bonferroni.

Univariate Tests

Measure: MEASURE_1

	Sum of Squares	df	Mean Square	F	Sig.	Partial Eta Squared	Noncent. Parameter	Observed Power[a]
Contrast	207.697	3	69.232	2.521	.070	.147	7.563	.584
Error	1208.384	44	27.463					

The F tests the effect of groups. This test is based on the linearly independent pairwise comparisons among the estimated marginal means.

a. Computed using alpha = .05

3. fatigue

Estimates

Measure: MEASURE_1

fatigue	Mean	Std. Error	95% Confidence Interval Lower Bound	95% Confidence Interval Upper Bound
1	9.220	.770	7.668	10.772
2	9.641	.931	7.765	11.517

Pairwise Comparisons

Measure: MEASURE_1

(I) fatigue	(J) fatigue	Mean Difference (I-J)	Std. Error	Sig.[a]	95% Confidence Interval for Difference[a] Lower Bound	95% Confidence Interval for Difference[a] Upper Bound
1	2	-.421	.728	.566	-1.889	1.046
2	1	.421	.728	.566	-1.046	1.889

Based on estimated marginal means

a. Adjustment for multiple comparisons: Bonferroni.

Multivariate Tests

	Value	F	Hypothesis df	Error df	Sig.	Partial Eta Squared	Noncent. Parameter	Observed Power[b]
Pillai's trace	.008	.335[a]	1.000	44.000	.566	.008	.335	.087
Wilks' lambda	.992	.335[a]	1.000	44.000	.566	.008	.335	.087
Hotelling's trace	.008	.335[a]	1.000	44.000	.566	.008	.335	.087
Roy's largest root	.008	.335[a]	1.000	44.000	.566	.008	.335	.087

Each F tests the multivariate effect of fatigue. These tests are based on the linearly independent pairwise comparisons among the estimated marginal means.

a. Exact statistic

b. Computed using alpha = .05

4. groups * fatigue

Measure: MEASURE_1

groups	fatigue	Mean	Std. Error	95% Confidence Interval Lower Bound	95% Confidence Interval Upper Bound
1.00	1	6.796	1.741	3.287	10.305

	2	7.025	2.104	2.784	11.265
2.00	1	10.197	1.306	7.566	12.829
	2	9.422	1.578	6.241	12.602
3.00	1	11.986	1.508	8.947	15.025
	2	13.569	1.822	9.897	17.241
4.00	1	7.900	1.575	4.726	11.074
	2	8.549	1.903	4.713	12.384

Post Hoc Tests

groups

Multiple Comparisons

Measure: MEASURE_1
Tukey HSD

(I) groups	(J) groups	Mean Difference (I-J)	Std. Error	Sig.	95% Confidence Interval	
					Lower Bound	Upper Bound
1.00	2.00	-2.8992	2.18356	.551	-8.7293	2.9310
	3.00	-5.8672	2.31086	.068	-12.0372	.3028
	4.00	-1.3139	2.35545	.944	-7.6030	4.9752
2.00	1.00	2.8992	2.18356	.551	-2.9310	8.7293
	3.00	-2.9681	2.00127	.456	-8.3114	2.3753
	4.00	1.5852	2.05259	.867	-3.8952	7.0657
3.00	1.00	5.8672	2.31086	.068	-.3028	12.0372
	2.00	2.9681	2.00127	.456	-2.3753	8.3114
	4.00	4.5533	2.18753	.175	-1.2874	10.3940
4.00	1.00	1.3139	2.35545	.944	-4.9752	7.6030
	2.00	-1.5852	2.05259	.867	-7.0657	3.8952
	3.00	-4.5533	2.18753	.175	-10.3940	1.2874

Based on observed means.
The error term is Mean Square(Error) = 27.463.

Homogeneous Subsets

MEASURE_1

Tukey HSD[a,b,c]

groups	N	Subset	
		1	2
1.00	10	6.9103	
4.00	15	8.2242	8.2242
2.00	16	9.8095	9.8095
3.00	12		12.7776
Sig.		.551	.174

Means for groups in homogeneous subsets are displayed.
Based on observed means.
The error term is Mean Square(Error) = 27.463.
a. Uses Harmonic Mean Sample Size = 11.499.
b. The group sizes are unequal. The harmonic mean of the group sizes is used. Type I error levels are not guaranteed.
c. Alpha = .05.

Vastus Lateralis (VL) - Early Stance

```
GLM NvlLRpre NvlLRpos BY groups
  /WSFACTOR=fatigue 2 Polynomial
  /METHOD=SSTYPE(3)
  /POSTHOC=groups(TUKEY)
  /EMMEANS=TABLES(OVERALL)
  /EMMEANS=TABLES(groups) COMPARE ADJ(BONFERRONI)
  /EMMEANS=TABLES(fatigue) COMPARE ADJ(BONFERRONI)
  /EMMEANS=TABLES(groups*fatigue)
  /PRINT=DESCRIPTIVE ETASQ OPOWER PARAMETER HOMOGENEITY
  /CRITERIA=ALPHA(.05)
  /WSDESIGN=fatigue
  /DESIGN=groups.
```

General Linear Model

Notes

Output Created		17-MAY-2026 21:49:41
Comments		
Input	Data	C:\Users\RaSa\Desktop\پروپوزال\New folder\data\emgmi1.sav
	Active Dataset	DataSet2
	Filter	<none>
	Weight	<none>
	Split File	<none>
	N of Rows in Working Data File	48
Missing Value Handling	Definition of Missing	User-defined missing values are treated as missing.
	Cases Used	Statistics are based on all cases with valid data for all variables in the model.

Syntax		GLM NvlLRpre NvlLRpos BY groups<br>/WSFACTOR=fatigue 2 Polynomial<br>/METHOD=SSTYPE(3)<br>/POSTHOC=groups(TUKEY)<br>/EMMEANS=TABLES(OVERALL)<br>/EMMEANS=TABLES(groups) COMPARE ADJ(BONFERRONI)<br>/EMMEANS=TABLES(fatigue) COMPARE ADJ(BONFERRONI)<br>/EMMEANS=TABLES(groups*fatigue)<br>/PRINT=DESCRIPTIVE ETASQ OPOWER PARAMETER HOMOGENEITY<br>/CRITERIA=ALPHA(.05)<br>/WSDESIGN=fatigue<br>/DESIGN=groups.
Resources	Processor Time	00:00:00.03
	Elapsed Time	00:00:00.03

Within-Subjects Factors

Measure: MEASURE_1

fatigue	Dependent Variable
1	NvlLRpre
2	NvlLRpos

Between-Subjects Factors

		N
groups	1.00	10
	2.00	16
	3.00	12
	4.00	15

Descriptive Statistics

	groups	Mean	Std. Deviation	N
NvILRpre	1.00	27.1388	22.04167	10
	2.00	14.5901	10.79458	16
	3.00	17.4550	13.21612	12
	4.00	13.3862	10.82154	15
	Total	17.3833	14.50170	53
NvILRpos	1.00	18.6483	12.70640	10
	2.00	13.5639	12.10761	16
	3.00	15.0361	10.49954	12
	4.00	18.5726	18.37837	15
	Total	16.0331	13.30717	53

Box's Test of Equality of Covariance Matrices[a]

Box's M	24.105
F	2.444
df1	9
df2	12234.839
Sig.	.009

Tests the null hypothesis that the observed covariance matrices of the dependent variables are equal across groups.
a. Design: Intercept + groups
Within Subjects Design: fatigue

Multivariate Tests[a]

Effect		Value	F	Hypothesis df	Error df	Sig.	Partial Eta Squared	Noncent. Parameter	Observed Power[c]
fatigue	Pillai's Trace	.011	.467[b]	1.000	44.000	.498	.011	.467	.103
	Wilks' Lambda	.989	.467[b]	1.000	44.000	.498	.011	.467	.103
	Hotelling's Trace	.011	.467[b]	1.000	44.000	.498	.011	.467	.103
	Roy's Largest Root	.011	.467[b]	1.000	44.000	.498	.011	.467	.103
fatigue * groups	Pillai's Trace	.071	1.123[b]	3.000	44.000	.350	.071	3.370	.282
	Wilks' Lambda	.929	1.123[b]	3.000	44.000	.350	.071	3.370	.282
	Hotelling's Trace	.077	1.123[b]	3.000	44.000	.350	.071	3.370	.282

	Roy's Largest Root	.077	1.123[b]	3.000	44.000	.350	.071	3.370	.282

a. Design: Intercept + groups
Within Subjects Design: fatigue
b. Exact statistic
c. Computed using alpha = .05

Mauchly's Test of Sphericity[a]

Measure: MEASURE_1

Within Subjects Effect	Mauchly's W	Approx. Chi-Square	df	Sig.	Epsilon[b] Greenhouse-Geisser	Huynh-Feldt	Lower-bound
fatigue	1.000	.000	0	.	1.000	1.000	1.000

Tests the null hypothesis that the error covariance matrix of the orthonormalized transformed dependent variables is proportional to an identity matrix.
a. Design: Intercept + groups
Within Subjects Design: fatigue
b. May be used to adjust the degrees of freedom for the averaged tests of significance. Corrected tests are displayed in the Tests of Within-Subjects Effects table.

Tests of Within-Subjects Effects

Measure: MEASURE_1

Source		Type III Sum of Squares	df	Mean Square	F	Sig.	Partial Eta Squared	Noncent. Parameter	Observed Power[a]
fatigue	Sphericity Assumed	65.474	1	65.474	.467	.498	.011	.467	.103
	Greenhouse-Geisser	65.474	1.000	65.474	.467	.498	.011	.467	.103
	Huynh-Feldt	65.474	1.000	65.474	.467	.498	.011	.467	.103
	Lower-bound	65.474	1.000	65.474	.467	.498	.011	.467	.103
fatigue * groups	Sphericity Assumed	472.115	3	157.372	1.123	.350	.071	3.370	.282
	Greenhouse-Geisser	472.115	3.000	157.372	1.123	.350	.071	3.370	.282
	Huynh-Feldt	472.115	3.000	157.372	1.123	.350	.071	3.370	.282
	Lower-bound	472.115	3.000	157.372	1.123	.350	.071	3.370	.282
Error(fatigue)	Sphericity Assumed	6164.636	44	140.105					
	Greenhouse-Geisser	6164.636	44.000	140.105					
	Huynh-Feldt	6164.636	44.000	140.105					
	Lower-bound	6164.636	44.000	140.105					

a. Computed using alpha = .05

Tests of Within-Subjects Contrasts

Measure: MEASURE_1

Source	fatigue	Type III Sum of Squares	df	Mean Square	F	Sig.	Partial Eta Squared	Noncent. Parameter	Observed Power[a]
fatigue	Linear	65.474	1	65.474	.467	.498	.011	.467	.103
fatigue * groups	Linear	472.115	3	157.372	1.123	.350	.071	3.370	.282
Error(fatigue)	Linear	6164.636	44	140.105					

a. Computed using alpha = .05

Levene's Test of Equality of Error Variances[a]

		Levene Statistic	df1	df2	Sig.
NvILRpre	Based on Mean	1.430	3	44	.247
	Based on Median	1.019	3	44	.393
	Based on Median and with adjusted df	1.019	3	28.859	.399
	Based on trimmed mean	1.369	3	44	.265
NvILRpos	Based on Mean	1.244	3	44	.305
	Based on Median	.624	3	44	.603
	Based on Median and with adjusted df	.624	3	28.908	.605
	Based on trimmed mean	1.181	3	44	.328

Tests the null hypothesis that the error variance of the dependent variable is equal across groups.

a. Design: Intercept + groups
Within Subjects Design: fatigue

Tests of Between-Subjects Effects

Measure: MEASURE_1
Transformed Variable: Average

Source	Type III Sum of Squares	df	Mean Square	F	Sig.	Partial Eta Squared	Noncent. Parameter	Observed Power[a]
Intercept	27528.929	1	27528.929	113.808	.000	.721	113.808	1.000
groups	927.019	3	309.006	1.277	.294	.080	3.832	.317
Error	10643.090	44	241.888					

a. Computed using alpha = .05

Parameter Estimates

Dependent Variable	Parameter	B	Std. Error	t	Sig.	95% Confidence Interval Lower Bound	95% Confidence Interval Upper Bound	Partial Eta Squared	Noncent. Parameter	Observed
NvILRpre	Intercept	13.386	4.246	3.152	.003	4.828	21.944	.184	3.152	
	[groups=1.00]	13.753	6.330	2.173	.035	.995	26.510	.097	2.173	
	[groups=2.00]	1.204	5.516	.218	.828	-9.913	12.321	.001	.218	
	[groups=3.00]	4.069	5.879	.692	.492	-7.779	15.917	.011	.692	
	[groups=4.00]	0[a]	.	.	.	.	.	.	.	
NvILRpos	Intercept	18.573	4.086	4.545	.000	10.338	26.808	.320	4.545	
	[groups=1.00]	.076	6.091	.012	.990	-12.200	12.352	.000	.012	
	[groups=2.00]	-5.009	5.308	-.944	.351	-15.706	5.689	.020	.944	
	[groups=3.00]	-3.536	5.657	-.625	.535	-14.937	7.864	.009	.625	
	[groups=4.00]	0[a]	.	.	.	.	.	.	.	

a. This parameter is set to zero because it is redundant.

b. Computed using alpha = .05

Estimated Marginal Means

1. Grand Mean

Measure: MEASURE_1

Mean	Std. Error	95% Confidence Interval Lower Bound	95% Confidence Interval Upper Bound
17.299	1.622	14.031	20.567

2. groups

Estimates

Measure: MEASURE_1

groups	Mean	Std. Error	95% Confidence Interval

			Lower Bound	Upper Bound
1.00	22.894	3.666	15.506	30.282
2.00	14.077	2.749	8.536	19.618
3.00	16.246	3.175	9.847	22.644
4.00	15.979	3.316	9.297	22.662

Pairwise Comparisons

Measure: MEASURE_1

(I) groups	(J) groups	Mean Difference (I-J)	Std. Error	Sig.[a]	95% Confidence Interval for Difference[a] Lower Bound	Upper Bound
1.00	2.00	8.817	4.582	.365	-3.843	21.477
	3.00	6.648	4.849	1.000	-6.750	20.046
	4.00	6.914	4.943	1.000	-6.742	20.571
2.00	1.00	-8.817	4.582	.365	-21.477	3.843
	3.00	-2.169	4.200	1.000	-13.772	9.435
	4.00	-1.902	4.307	1.000	-13.803	9.998
3.00	1.00	-6.648	4.849	1.000	-20.046	6.750
	2.00	2.169	4.200	1.000	-9.435	13.772
	4.00	.266	4.591	1.000	-12.417	12.949
4.00	1.00	-6.914	4.943	1.000	-20.571	6.742
	2.00	1.902	4.307	1.000	-9.998	13.803
	3.00	-.266	4.591	1.000	-12.949	12.417

Based on estimated marginal means
a. Adjustment for multiple comparisons: Bonferroni.

Univariate Tests

Measure: MEASURE_1

	Sum of Squares	df	Mean Square	F	Sig.	Partial Eta Squared	Noncent. Parameter	Observed Power[a]
Contrast	463.509	3	154.503	1.277	.294	.080	3.832	.317
Error	5321.545	44	120.944					

The F tests the effect of groups. This test is based on the linearly independent pairwise comparisons among the estimated marginal means.
a. Computed using alpha = .05

3. fatigue

Estimates

Measure: MEASURE_1

fatigue	Mean	Std. Error	95% Confidence Interval	
			Lower Bound	Upper Bound
1	18.143	2.077	13.958	22.328
2	16.455	1.998	12.428	20.482

Pairwise Comparisons

Measure: MEASURE_1

(I) fatigue	(J) fatigue	Mean Difference (I-J)	Std. Error	Sig.[a]	95% Confidence Interval for Difference[a]	
					Lower Bound	Upper Bound
1	2	1.687	2.468	.498	-3.287	6.662
2	1	-1.687	2.468	.498	-6.662	3.287

Based on estimated marginal means

a. Adjustment for multiple comparisons: Bonferroni.

Multivariate Tests

	Value	F	Hypothesis df	Error df	Sig.	Partial Eta Squared	Noncent. Parameter	Observed Power[b]
Pillai's trace	.011	.467[a]	1.000	44.000	.498	.011	.467	.103
Wilks' lambda	.989	.467[a]	1.000	44.000	.498	.011	.467	.103
Hotelling's trace	.011	.467[a]	1.000	44.000	.498	.011	.467	.103
Roy's largest root	.011	.467[a]	1.000	44.000	.498	.011	.467	.103

Each F tests the multivariate effect of fatigue. These tests are based on the linearly independent pairwise comparisons among the estimated marginal means.

a. Exact statistic

b. Computed using alpha = .05

4. groups * fatigue

Measure: MEASURE_1

groups	fatigue	Mean	Std. Error	95% Confidence Interval	
				Lower Bound	Upper Bound
1.00	1	27.139	4.694	17.678	36.600

	2	18.648	4.517	9.544	27.752
2.00	1	14.590	3.521	7.494	21.686
	2	13.564	3.388	6.736	20.392
3.00	1	17.455	4.065	9.262	25.648
	2	15.036	3.912	7.152	22.920
4.00	1	13.386	4.246	4.828	21.944
	2	18.573	4.086	10.338	26.808

Post Hoc Tests

groups

Multiple Comparisons

Measure: MEASURE_1
Tukey HSD

(I) groups	(J) groups	Mean Difference (I-J)	Std. Error	Sig.	95% Confidence Interval Lower Bound	Upper Bound
1.00	2.00	8.8165	4.58228	.233	-3.4182	21.0512
	3.00	6.6480	4.84943	.524	-6.3000	19.5960
	4.00	6.9142	4.94299	.507	-6.2836	20.1120
2.00	1.00	-8.8165	4.58228	.233	-21.0512	3.4182
	3.00	-2.1685	4.19973	.955	-13.3818	9.0448
	4.00	-1.9024	4.30743	.971	-13.4032	9.5985
3.00	1.00	-6.6480	4.84943	.524	-19.5960	6.3000
	2.00	2.1685	4.19973	.955	-9.0448	13.3818
	4.00	.2662	4.59060	1.000	-11.9908	12.5231
4.00	1.00	-6.9142	4.94299	.507	-20.1120	6.2836
	2.00	1.9024	4.30743	.971	-9.5985	13.4032
	3.00	-.2662	4.59060	1.000	-12.5231	11.9908

Based on observed means.
The error term is Mean Square(Error) = 120.944.

Homogeneous Subsets

MEASURE_1

Tukey HSD[a,b,c]

groups	N	Subset 1
2.00	16	14.0770
4.00	15	15.9794
3.00	12	16.2455
1.00	10	22.8936
Sig.		.234

Means for groups in homogeneous subsets are displayed.
Based on observed means.
The error term is Mean Square(Error) = 120.944.
a. Uses Harmonic Mean Sample Size = 11.499.
b. The group sizes are unequal. The harmonic mean of the group sizes is used. Type I error levels are not guaranteed.
c. Alpha = .05.

Rectus Femoris (RF) - Early Stance

```
GLM NrfLRpre NrfLRpos BY groups
  /WSFACTOR=fatigue 2 Polynomial
  /METHOD=SSTYPE(3)
  /POSTHOC=groups(TUKEY)
  /EMMEANS=TABLES(OVERALL)
  /EMMEANS=TABLES(groups) COMPARE ADJ(BONFERRONI)
  /EMMEANS=TABLES(fatigue) COMPARE ADJ(BONFERRONI)
  /EMMEANS=TABLES(groups*fatigue)
  /PRINT=DESCRIPTIVE ETASQ OPOWER PARAMETER HOMOGENEITY
  /CRITERIA=ALPHA(.05)
  /WSDESIGN=fatigue
  /DESIGN=groups.
```

General Linear Model

Notes

Output Created		17-MAY-2026 21:27:15
Comments		
Input	Data	C:\Users\RaSa\Desktop\پروپوزال\New folder\data\emgmi1.sav
	Active Dataset	DataSet2
	Filter	<none>
	Weight	<none>
	Split File	<none>
	N of Rows in Working Data File	48
Missing Value Handling	Definition of Missing	User-defined missing values are treated as missing.
	Cases Used	Statistics are based on all cases with valid data for all variables in the model.

Syntax		GLM NrfLRpre NrfLRpos BY groups<br>/WSFACTOR=fatigue 2 Polynomial<br>/METHOD=SSTYPE(3)<br>/POSTHOC=groups(TUKEY)<br>/EMMEANS=TABLES(OVERALL)<br>/EMMEANS=TABLES(groups) COMPARE ADJ(BONFERRONI)<br>/EMMEANS=TABLES(fatigue) COMPARE ADJ(BONFERRONI)<br>/EMMEANS=TABLES(groups*fatigue)<br>/PRINT=DESCRIPTIVE ETASQ OPOWER PARAMETER HOMOGENEITY<br>/CRITERIA=ALPHA(.05)<br>/WSDESIGN=fatigue<br>/DESIGN=groups.
Resources	Processor Time	00:00:00.03
	Elapsed Time	00:00:00.03

Within-Subjects Factors

Measure: MEASURE_1

fatigue	Dependent Variable
1	NrfLRpre
2	NrfLRpos

Between-Subjects Factors

		N
groups	1.00	10
	2.00	16
	3.00	12
	4.00	15

Descriptive Statistics

	groups	Mean	Std. Deviation	N
NrfLRpre	1.00	23.5026	18.44864	10
	2.00	17.0842	12.57746	16
	3.00	17.5380	13.77894	12
	4.00	10.7110	7.88165	15
	Total	16.9406	13.54614	53
NrfLRpos	1.00	22.3622	16.31520	10
	2.00	15.2744	15.13193	16
	3.00	11.7729	7.95613	12
	4.00	12.1352	4.68070	15
	Total	15.0085	12.36310	53

Box's Test of Equality of Covariance Matrices[a]

Box's M	23.370
F	2.370
df1	9
df2	12234.839
Sig.	.011

Tests the null hypothesis that the observed covariance matrices of the dependent variables are equal across groups.

a. Design: Intercept + groups
Within Subjects Design: fatigue

Multivariate Tests[a]

Effect		Value	F	Hypothesis df	Error df	Sig.	Partial Eta Squared	Noncent. Parameter	Observed Power[c]
fatigue	Pillai's Trace	.039	1.805[b]	1.000	44.000	.186	.039	1.805	.260
	Wilks' Lambda	.961	1.805[b]	1.000	44.000	.186	.039	1.805	.260
	Hotelling's Trace	.041	1.805[b]	1.000	44.000	.186	.039	1.805	.260
	Roy's Largest Root	.041	1.805[b]	1.000	44.000	.186	.039	1.805	.260
fatigue * groups	Pillai's Trace	.076	1.205[b]	3.000	44.000	.319	.076	3.615	.301
	Wilks' Lambda	.924	1.205[b]	3.000	44.000	.319	.076	3.615	.301

	Hotelling's Trace	.082	1.205[b]	3.000	44.000	.319	.076	3.615	.301
	Roy's Largest Root	.082	1.205[b]	3.000	44.000	.319	.076	3.615	.301

a. Design: Intercept + groups
Within Subjects Design: fatigue
b. Exact statistic
c. Computed using alpha = .05

Mauchly's Test of Sphericity[a]

Measure: MEASURE_1

Within Subjects Effect	Mauchly's W	Approx. Chi-Square	df	Sig.	Epsilon[b] Greenhouse-Geisser	Huynh-Feldt	Lower-bound
fatigue	1.000	.000	0	.	1.000	1.000	1.000

Tests the null hypothesis that the error covariance matrix of the orthonormalized transformed dependent variables is proportional to an identity matrix.
a. Design: Intercept + groups
Within Subjects Design: fatigue
b. May be used to adjust the degrees of freedom for the averaged tests of significance. Corrected tests are displayed in the Tests of Within-Subjects Effects table.

Tests of Within-Subjects Effects

Measure: MEASURE_1

Source		Type III Sum of Squares	df	Mean Square	F	Sig.	Partial Eta Squared	Noncent. Parameter	Observed Power[a]
fatigue	Sphericity Assumed	76.413	1	76.413	1.805	.186	.039	1.805	.260
	Greenhouse-Geisser	76.413	1.000	76.413	1.805	.186	.039	1.805	.260
	Huynh-Feldt	76.413	1.000	76.413	1.805	.186	.039	1.805	.260
	Lower-bound	76.413	1.000	76.413	1.805	.186	.039	1.805	.260
fatigue * groups	Sphericity Assumed	153.050	3	51.017	1.205	.319	.076	3.615	.301
	Greenhouse-Geisser	153.050	3.000	51.017	1.205	.319	.076	3.615	.301
	Huynh-Feldt	153.050	3.000	51.017	1.205	.319	.076	3.615	.301
	Lower-bound	153.050	3.000	51.017	1.205	.319	.076	3.615	.301
Error(fatigue)	Sphericity Assumed	1862.985	44	42.341					
	Greenhouse-Geisser	1862.985	44.000	42.341					
	Huynh-Feldt	1862.985	44.000	42.341					
	Lower-bound	1862.985	44.000	42.341					

a. Computed using alpha = .05

Tests of Within-Subjects Contrasts

Measure: MEASURE_1

Source	fatigue	Type III Sum of Squares	df	Mean Square	F	Sig.	Partial Eta Squared	Noncent. Parameter	Observed Power[a]
fatigue	Linear	76.413	1	76.413	1.805	.186	.039	1.805	.260
fatigue * groups	Linear	153.050	3	51.017	1.205	.319	.076	3.615	.301
Error(fatigue)	Linear	1862.985	44	42.341					

a. Computed using alpha = .05

Levene's Test of Equality of Error Variances[a]

		Levene Statistic	df1	df2	Sig.
NrfLRpre	Based on Mean	1.968	3	44	.133
	Based on Median	1.234	3	44	.309
	Based on Median and with adjusted df	1.234	3	34.579	.312
	Based on trimmed mean	1.741	3	44	.172
NrfLRpos	Based on Mean	4.149	3	44	.011
	Based on Median	1.501	3	44	.227
	Based on Median and with adjusted df	1.501	3	28.484	.235
	Based on trimmed mean	3.359	3	44	.027

Tests the null hypothesis that the error variance of the dependent variable is equal across groups.

a. Design: Intercept + groups
Within Subjects Design: fatigue

Tests of Between-Subjects Effects

Measure: MEASURE_1
Transformed Variable: Average

Source	Type III Sum of Squares	df	Mean Square	F	Sig.	Partial Eta Squared	Noncent. Parameter	Observed Power[a]
Intercept	24434.150	1	24434.150	86.549	.000	.663	86.549	1.000
groups	1370.258	3	456.753	1.618	.199	.099	4.854	.395
Error	12421.880	44	282.315					

a. Computed using alpha = .05

Parameter Estimates

Dependent Variable	Parameter	B	Std. Error	t	Sig.	95% Confidence Interval		Partial Eta Squared	Noncent. Parameter	Observed Power[b]
						Lower Bound	Upper Bound			
NrfLRpre	Intercept	10.711	4.016	2.667	.011	2.618	18.804	.139	2.667	.742
	[groups=1.00]	12.792	5.986	2.137	.038	.727	24.856	.094	2.137	.552
	[groups=2.00]	6.373	5.217	1.222	.228	-4.140	16.887	.033	1.222	.223
	[groups=3.00]	6.827	5.560	1.228	.226	-4.378	18.032	.033	1.228	.225
	[groups=4.00]	0[a]	.	.	.	.	.	.	.	.
NrfLRpos	Intercept	12.135	3.659	3.317	.002	4.761	19.509	.200	3.317	.900
	[groups=1.00]	10.227	5.454	1.875	.067	-.765	21.220	.074	1.875	.450
	[groups=2.00]	3.139	4.753	.660	.512	-6.440	12.718	.010	.660	.099
	[groups=3.00]	-.362	5.065	-.072	.943	-10.571	9.847	.000	.072	.051
	[groups=4.00]	0[a]	.	.	.	.	.	.	.	.

a. This parameter is set to zero because it is redundant.

b. Computed using alpha = .05

Estimated Marginal Means

1. Grand Mean

Measure: MEASURE_1

Mean	Std. Error	95% Confidence Interval	
		Lower Bound	Upper Bound
16.298	1.752	12.767	19.828

2. groups

Estimates

Measure: MEASURE_1

groups	Mean	Std. Error	95% Confidence Interval

			Lower Bound	Upper Bound
1.00	22.932	3.960	14.951	30.914
2.00	16.179	2.970	10.193	22.165
3.00	14.655	3.430	7.743	21.568
4.00	11.423	3.582	4.204	18.643

Pairwise Comparisons

Measure: MEASURE_1

(I) groups	(J) groups	Mean Difference (I-J)	Std. Error	Sig.[a]	95% Confidence Interval for Difference[a] Lower Bound	Upper Bound
1.00	2.00	6.753	4.950	1.000	-6.924	20.430
	3.00	8.277	5.239	.728	-6.198	22.751
	4.00	11.509	5.340	.220	-3.244	26.263
2.00	1.00	-6.753	4.950	1.000	-20.430	6.924
	3.00	1.524	4.537	1.000	-11.011	14.059
	4.00	4.756	4.653	1.000	-8.101	17.613
3.00	1.00	-8.277	5.239	.728	-22.751	6.198
	2.00	-1.524	4.537	1.000	-14.059	11.011
	4.00	3.232	4.959	1.000	-10.470	16.934
4.00	1.00	-11.509	5.340	.220	-26.263	3.244
	2.00	-4.756	4.653	1.000	-17.613	8.101
	3.00	-3.232	4.959	1.000	-16.934	10.470

Based on estimated marginal means

a. Adjustment for multiple comparisons: Bonferroni.

Univariate Tests

Measure: MEASURE_1

	Sum of Squares	df	Mean Square	F	Sig.	Partial Eta Squared	Noncent. Parameter	Observed Power[a]
Contrast	685.129	3	228.376	1.618	.199	.099	4.854	.395
Error	6210.940	44	141.158					

The F tests the effect of groups. This test is based on the linearly independent pairwise comparisons among the estimated marginal means.

a. Computed using alpha = .05

3. fatigue

Estimates

Measure: MEASURE_1

fatigue	Mean	Std. Error	95% Confidence Interval Lower Bound	95% Confidence Interval Upper Bound
1	17.209	1.964	13.251	21.167
2	15.386	1.789	11.780	18.992

Pairwise Comparisons

Measure: MEASURE_1

(I) fatigue	(J) fatigue	Mean Difference (I-J)	Std. Error	Sig.[a]	95% Confidence Interval for Difference[a] Lower Bound	95% Confidence Interval for Difference[a] Upper Bound
1	2	1.823	1.357	.186	-.912	4.557
2	1	-1.823	1.357	.186	-4.557	.912

Based on estimated marginal means

a. Adjustment for multiple comparisons: Bonferroni.

Multivariate Tests

	Value	F	Hypothesis df	Error df	Sig.	Partial Eta Squared	Noncent. Parameter	Observed Power[b]
Pillai's trace	.039	1.805[a]	1.000	44.000	.186	.039	1.805	.260
Wilks' lambda	.961	1.805[a]	1.000	44.000	.186	.039	1.805	.260
Hotelling's trace	.041	1.805[a]	1.000	44.000	.186	.039	1.805	.260
Roy's largest root	.041	1.805[a]	1.000	44.000	.186	.039	1.805	.260

Each F tests the multivariate effect of fatigue. These tests are based on the linearly independent pairwise comparisons among the estimated marginal means.

a. Exact statistic

b. Computed using alpha = .05

4. groups * fatigue

Measure: MEASURE_1

groups	fatigue	Mean	Std. Error	95% Confidence Interval Lower Bound	95% Confidence Interval Upper Bound
1.00	1	23.503	4.440	14.555	32.450
	2	22.362	4.045	14.210	30.514

2.00	1	17.084	3.330	10.374	23.795
	2	15.274	3.034	9.160	21.389
3.00	1	17.538	3.845	9.789	25.287
	2	11.773	3.503	4.713	18.833
4.00	1	10.711	4.016	2.618	18.804
	2	12.135	3.659	4.761	19.509

Post Hoc Tests

groups

Multiple Comparisons

Measure: MEASURE_1
Tukey HSD

(I) groups	(J) groups	Mean Difference (I-J)	Std. Error	Sig.	95% Confidence Interval Lower Bound	Upper Bound
1.00	2.00	6.7531	4.95041	.528	-6.4645	19.9707
	3.00	8.2769	5.23902	.400	-5.7113	22.2651
	4.00	11.5093	5.34010	.152	-2.7488	25.7674
2.00	1.00	-6.7531	4.95041	.528	-19.9707	6.4645
	3.00	1.5238	4.53712	.987	-10.5903	13.6380
	4.00	4.7562	4.65348	.737	-7.6686	17.1810
3.00	1.00	-8.2769	5.23902	.400	-22.2651	5.7113
	2.00	-1.5238	4.53712	.987	-13.6380	10.5903
	4.00	3.2324	4.95940	.914	-10.0092	16.4740
4.00	1.00	-11.5093	5.34010	.152	-25.7674	2.7488
	2.00	-4.7562	4.65348	.737	-17.1810	7.6686
	3.00	-3.2324	4.95940	.914	-16.4740	10.0092

Based on observed means.
The error term is Mean Square(Error) = 141.158.

Homogeneous Subsets

MEASURE_1

Tukey HSD[a,b,c]

groups	N	Subset 1
4.00	15	11.4231
3.00	12	14.6554
2.00	16	16.1793
1.00	10	22.9324
Sig.		.108

Means for groups in homogeneous subsets are displayed.
Based on observed means.
The error term is Mean Square(Error) = 141.158.
a. Uses Harmonic Mean Sample Size = 11.499.
b. The group sizes are unequal. The harmonic mean of the group sizes is used. Type I error levels are not guaranteed.
c. Alpha = .05.

Vastus Medialis (VM) - Early Stance

```
GLM NvmLRpre NvmLRpos BY groups
  /WSFACTOR=fatigue 2 Polynomial
  /METHOD=SSTYPE(3)
  /POSTHOC=groups(TUKEY)
  /EMMEANS=TABLES(OVERALL)
  /EMMEANS=TABLES(groups) COMPARE ADJ(BONFERRONI)
  /EMMEANS=TABLES(fatigue) COMPARE ADJ(BONFERRONI)
  /EMMEANS=TABLES(groups*fatigue)
  /PRINT=DESCRIPTIVE ETASQ OPOWER PARAMETER HOMOGENEITY
  /CRITERIA=ALPHA(.05)
  /WSDESIGN=fatigue
  /DESIGN=groups.
```

General Linear Model

Notes

Output Created		17-MAY-2026 21:27:35
Comments		
Input	Data	C:\Users\RaSa\Desk-top\پروپوزال\New folder\data\emgmi1.sav
	Active Dataset	DataSet2
	Filter	<none>
	Weight	<none>
	Split File	<none>
	N of Rows in Working Data File	48
Missing Value Handling	Definition of Missing	User-defined missing values are treated as missing.
	Cases Used	Statistics are based on all cases with valid data for all variables in the model.

Syntax		GLM NvmLRpre NvmLRpos BY groups /WSFACTOR=fatigue 2 Polynomial /METHOD=SSTYPE(3) /POSTHOC=groups(TUKEY) /EMMEANS=TABLES(OVERALL) /EMMEANS=TABLES(groups) COMPARE ADJ(BONFERRONI) /EMMEANS=TABLES(fatigue) COMPARE ADJ(BONFERRONI) /EMMEANS=TABLES(groups*fatigue) /PRINT=DESCRIPTIVE ETASQ OPOWER PARAMETER HOMOGENEITY /CRITERIA=ALPHA(.05) /WSDESIGN=fatigue /DESIGN=groups.
Resources	Processor Time	00:00:00.05
	Elapsed Time	00:00:00.05

Within-Subjects Factors

Measure: MEASURE_1

fatigue	Dependent Variable
1	NvmLRpre
2	NvmLRpos

Between-Subjects Factors

		N
groups	1.00	10
	2.00	16
	3.00	12
	4.00	15

Descriptive Statistics

	groups	Mean	Std. Deviation	N
NvmLRpre	1.00	17.8815	12.23109	10
	2.00	14.0038	12.83438	16
	3.00	37.7901	81.17009	12
	4.00	8.1408	2.44081	15
	Total	19.3338	41.80524	53
NvmLRpos	1.00	16.5393	12.21783	10
	2.00	17.3792	18.61574	16
	3.00	12.2267	7.85815	12
	4.00	13.8745	13.23445	15
	Total	15.1304	13.86904	53

Box's Test of Equality of Covariance Matrices[a]

Box's M	119.914
F	12.160
df1	9
df2	12234.839
Sig.	.000

Tests the null hypothesis that the observed covariance matrices of the dependent variables are equal across groups.
a. Design: Intercept + groups
Within Subjects Design: fatigue

Multivariate Tests[a]

Effect		Value	F	Hypothesis df	Error df	Sig.	Partial Eta Squared	Noncent. Parameter	Observed Power[c]
fatigue	Pillai's Trace	.012	.551[b]	1.000	44.000	.462	.012	.551	.112
	Wilks' Lambda	.988	.551[b]	1.000	44.000	.462	.012	.551	.112
	Hotelling's Trace	.013	.551[b]	1.000	44.000	.462	.012	.551	.112
	Roy's Largest Root	.013	.551[b]	1.000	44.000	.462	.012	.551	.112
fatigue * groups	Pillai's Trace	.094	1.524[b]	3.000	44.000	.222	.094	4.571	.374
	Wilks' Lambda	.906	1.524[b]	3.000	44.000	.222	.094	4.571	.374

	Hotelling's Trace	.104	1.524[b]	3.000	44.000	.222	.094	4.571	.374
	Roy's Largest Root	.104	1.524[b]	3.000	44.000	.222	.094	4.571	.374

a. Design: Intercept + groups
 Within Subjects Design: fatigue
b. Exact statistic
c. Computed using alpha = .05

Mauchly's Test of Sphericity[a]

Measure: MEASURE_1

Within Subjects Effect	Mauchly's W	Approx. Chi-Square	df	Sig.	Epsilon[b] Greenhouse-Geisser	Huynh-Feldt	Lower-bound
fatigue	1.000	.000	0	.	1.000	1.000	1.000

Tests the null hypothesis that the error covariance matrix of the orthonormalized transformed dependent variables is proportional to an identity matrix.
a. Design: Intercept + groups
 Within Subjects Design: fatigue
b. May be used to adjust the degrees of freedom for the averaged tests of significance. Corrected tests are displayed in the Tests of Within-Subjects Effects table.

Tests of Within-Subjects Effects

Measure: MEASURE_1

Source		Type III Sum of Squares	df	Mean Square	F	Sig.	Partial Eta Squared	Noncent. Parameter	Observed Power[a]
fatigue	Sphericity Assumed	455.245	1	455.245	.551	.462	.012	.551	.112
	Greenhouse-Geisser	455.245	1.000	455.245	.551	.462	.012	.551	.112
	Huynh-Feldt	455.245	1.000	455.245	.551	.462	.012	.551	.112
	Lower-bound	455.245	1.000	455.245	.551	.462	.012	.551	.112
fatigue * groups	Sphericity Assumed	3776.951	3	1258.984	1.524	.222	.094	4.571	.374
	Greenhouse-Geisser	3776.951	3.000	1258.984	1.524	.222	.094	4.571	.374
	Huynh-Feldt	3776.951	3.000	1258.984	1.524	.222	.094	4.571	.374
	Lower-bound	3776.951	3.000	1258.984	1.524	.222	.094	4.571	.374
Error(fatigue)	Sphericity Assumed	36354.001	44	826.227					
	Greenhouse-Geisser	36354.001	44.000	826.227					
	Huynh-Feldt	36354.001	44.000	826.227					
	Lower-bound	36354.001	44.000	826.227					

a. Computed using alpha = .05

Tests of Within-Subjects Contrasts

Measure: MEASURE_1

Source	fatigue	Type III Sum of Squares	df	Mean Square	F	Sig.	Partial Eta Squared	Noncent. Parameter	Observed Power[a]
fatigue	Linear	455.245	1	455.245	.551	.462	.012	.551	.112
fatigue * groups	Linear	3776.951	3	1258.984	1.524	.222	.094	4.571	.374
Error(fatigue)	Linear	36354.001	44	826.227					

a. Computed using alpha = .05

Levene's Test of Equality of Error Variances[a]

		Levene Statistic	df1	df2	Sig.
NvmLRpre	Based on Mean	3.472	3	44	.024
	Based on Median	1.065	3	44	.374
	Based on Median and with adjusted df	1.065	3	11.759	.401
	Based on trimmed mean	1.677	3	44	.186
NvmLRpos	Based on Mean	.366	3	44	.778
	Based on Median	.163	3	44	.920
	Based on Median and with adjusted df	.163	3	31.679	.920
	Based on trimmed mean	.199	3	44	.897

Tests the null hypothesis that the error variance of the dependent variable is equal across groups.
a. Design: Intercept + groups
Within Subjects Design: fatigue

Tests of Between-Subjects Effects

Measure: MEASURE_1
Transformed Variable: Average

Source	Type III Sum of Squares	df	Mean Square	F	Sig.	Partial Eta Squared	Noncent. Parameter	Observed Power[a]
Intercept	27308.513	1	27308.513	24.688	.000	.359	24.688	.998
groups	2379.625	3	793.208	.717	.547	.047	2.151	.190
Error	48670.760	44	1106.154					

a. Computed using alpha = .05

Parameter Estimates

Dependent Variable	Parameter	B	Std. Error	t	Sig.	95% Confidence Interval Lower Bound	95% Confidence Interval Upper Bound	Partial Eta Squared	Noncent. Parameter	Observed Power[b]
NvmLRpre	Intercept	8.141	12.548	.649	.520	-17.147	33.429	.009	.649	.097
	[groups=1.00]	9.741	18.705	.521	.605	-27.956	47.438	.006	.521	.080
	[groups=2.00]	5.863	16.300	.360	.721	-26.987	38.713	.003	.360	.064
	[groups=3.00]	29.649	17.371	1.707	.095	-5.360	64.659	.062	1.707	.386
	[groups=4.00]	0[a]	.	.	.	.	.	.	.	.
NvmLRpos	Intercept	13.875	4.270	3.250	.002	5.270	22.479	.194	3.250	.888
	[groups=1.00]	2.665	6.365	.419	.677	-10.163	15.492	.004	.419	.069
	[groups=2.00]	3.505	5.546	.632	.531	-7.673	14.683	.009	.632	.095
	[groups=3.00]	-1.648	5.911	-.279	.782	-13.561	10.265	.002	.279	.059
	[groups=4.00]	0[a]	.	.	.	.	.	.	.	.

a. This parameter is set to zero because it is redundant.

b. Computed using alpha = .05

Estimated Marginal Means

1. Grand Mean

Measure: MEASURE_1

Mean	Std. Error	95% Confidence Interval Lower Bound	95% Confidence Interval Upper Bound
17.229	3.468	10.241	24.218

2. groups

Estimates

Measure: MEASURE_1

groups	Mean	Std. Error	95% Confidence Interval

			Lower Bound	Upper Bound
1.00	17.210	7.839	1.412	33.009
2.00	15.691	5.879	3.842	27.541
3.00	25.008	6.789	11.326	38.691
4.00	11.008	7.091	-3.283	25.298

Pairwise Comparisons

Measure: MEASURE_1

(I) groups	(J) groups	Mean Difference (I-J)	Std. Error	Sig.[a]	95% Confidence Interval for Difference[a] Lower Bound	Upper Bound
1.00	2.00	1.519	9.799	1.000	-25.554	28.592
	3.00	-7.798	10.370	1.000	-36.449	20.853
	4.00	6.203	10.570	1.000	-23.001	35.407
2.00	1.00	-1.519	9.799	1.000	-28.592	25.554
	3.00	-9.317	8.981	1.000	-34.130	15.496
	4.00	4.684	9.211	1.000	-20.765	30.133
3.00	1.00	7.798	10.370	1.000	-20.853	36.449
	2.00	9.317	8.981	1.000	-15.496	34.130
	4.00	14.001	9.817	.965	-13.121	41.123
4.00	1.00	-6.203	10.570	1.000	-35.407	23.001
	2.00	-4.684	9.211	1.000	-30.133	20.765
	3.00	-14.001	9.817	.965	-41.123	13.121

Based on estimated marginal means
a. Adjustment for multiple comparisons: Bonferroni.

Univariate Tests

Measure: MEASURE_1

	Sum of Squares	df	Mean Square	F	Sig.	Partial Eta Squared	Noncent. Parameter	Observed Power[a]
Contrast	1189.813	3	396.604	.717	.547	.047	2.151	.190
Error	24335.380	44	553.077					

The F tests the effect of groups. This test is based on the linearly independent pairwise comparisons among the estimated marginal means.
a. Computed using alpha = .05

3. fatigue

Estimates

Measure: MEASURE_1

fatigue	Mean	Std. Error	95% Confidence Interval Lower Bound	95% Confidence Interval Upper Bound
1	19.454	6.136	7.088	31.821
2	15.005	2.088	10.797	19.213

Pairwise Comparisons

Measure: MEASURE_1

(I) fatigue	(J) fatigue	Mean Difference (I-J)	Std. Error	Sig.[a]	95% Confidence Interval for Difference[a] Lower Bound	95% Confidence Interval for Difference[a] Upper Bound
1	2	4.449	5.994	.462	-7.631	16.529
2	1	-4.449	5.994	.462	-16.529	7.631

Based on estimated marginal means

a. Adjustment for multiple comparisons: Bonferroni.

Multivariate Tests

	Value	F	Hypothesis df	Error df	Sig.	Partial Eta Squared	Noncent. Parameter	Observed Power[b]
Pillai's trace	.012	.551[a]	1.000	44.000	.462	.012	.551	.112
Wilks' lambda	.988	.551[a]	1.000	44.000	.462	.012	.551	.112
Hotelling's trace	.013	.551[a]	1.000	44.000	.462	.012	.551	.112
Roy's largest root	.013	.551[a]	1.000	44.000	.462	.012	.551	.112

Each F tests the multivariate effect of fatigue. These tests are based on the linearly independent pairwise comparisons among the estimated marginal means.

a. Exact statistic

b. Computed using alpha = .05

4. groups * fatigue

Measure: MEASURE_1

groups	fatigue	Mean	Std. Error	95% Confidence Interval Lower Bound	95% Confidence Interval Upper Bound
1.00	1	17.882	13.872	-10.075	45.838

	2	16.539	4.720	7.026	26.052
2.00	1	14.004	10.404	-6.964	34.971
	2	17.379	3.540	10.244	24.514
3.00	1	37.790	12.013	13.579	62.001
	2	12.227	4.088	3.988	20.465
4.00	1	8.141	12.548	-17.147	33.429
	2	13.875	4.270	5.270	22.479

Post Hoc Tests

groups

Multiple Comparisons

Measure: MEASURE_1
Tukey HSD

(I) groups	(J) groups	Mean Difference (I-J)	Std. Error	Sig.	95% Confidence Interval Lower Bound	Upper Bound
1.00	2.00	1.5189	9.79899	.999	-24.6445	27.6823
	3.00	-7.7980	10.37028	.875	-35.4867	19.8907
	4.00	6.2027	10.57037	.936	-22.0202	34.4257
2.00	1.00	-1.5189	9.79899	.999	-27.6823	24.6445
	3.00	-9.3169	8.98093	.729	-33.2961	14.6622
	4.00	4.6838	9.21124	.957	-19.9102	29.2779
3.00	1.00	7.7980	10.37028	.875	-19.8907	35.4867
	2.00	9.3169	8.98093	.729	-14.6622	33.2961
	4.00	14.0008	9.81679	.490	-12.2101	40.2116
4.00	1.00	-6.2027	10.57037	.936	-34.4257	22.0202
	2.00	-4.6838	9.21124	.957	-29.2779	19.9102
	3.00	-14.0008	9.81679	.490	-40.2116	12.2101

Based on observed means.
The error term is Mean Square(Error) = 553.077.

Homogeneous Subsets

MEASURE_1

Tukey HSD[a,b,c]

groups	N	Subset 1
4.00	15	11.0077
2.00	16	15.6915
1.00	10	17.2104
3.00	12	25.0084
Sig.		.489

Means for groups in homogeneous subsets are displayed.
Based on observed means.
The error term is Mean Square(Error) = 553.077.
a. Uses Harmonic Mean Sample Size = 11.499.
b. The group sizes are unequal. The harmonic mean of the group sizes is used. Type I error levels are not guaranteed.
c. Alpha = .05.

Biceps Femoris (BF) - Early Stance

```
GLM NbfLRpre NbfLRpos BY groups
  /WSFACTOR=fatigue 2 Polynomial
  /METHOD=SSTYPE(3)
  /POSTHOC=groups(TUKEY)
  /EMMEANS=TABLES(OVERALL)
  /EMMEANS=TABLES(groups) COMPARE ADJ(BONFERRONI)
  /EMMEANS=TABLES(fatigue) COMPARE ADJ(BONFERRONI)
  /EMMEANS=TABLES(groups*fatigue)
  /PRINT=DESCRIPTIVE ETASQ OPOWER PARAMETER HOMOGENEITY
  /CRITERIA=ALPHA(.05)
  /WSDESIGN=fatigue
  /DESIGN=groups.
```

General Linear Model

Notes

Output Created		17-MAY-2026 21:27:54
Comments		
Input	Data	C:\Users\RaSa\Desk-top\پروپوزال\New folder\data\emgmi1.sav
	Active Dataset	DataSet2
	Filter	<none>
	Weight	<none>
	Split File	<none>
	N of Rows in Working Data File	48
Missing Value Handling	Definition of Missing	User-defined missing values are treated as missing.
	Cases Used	Statistics are based on all cases with valid data for all variables in the model.

Syntax		GLM NbfLRpre NbfLRpos BY groups<br>/WSFACTOR=fatigue 2 Polynomial<br>/METHOD=SSTYPE(3)<br>/POSTHOC=groups(TUKEY)<br>/EMMEANS=TABLES(OVERALL)<br>/EMMEANS=TABLES(groups) COMPARE ADJ(BONFERRONI)<br>/EMMEANS=TABLES(fatigue) COMPARE ADJ(BONFERRONI)<br>/EMMEANS=TABLES(groups*fatigue)<br>/PRINT=DESCRIPTIVE ETASQ OPOWER PARAMETER HOMOGENEITY<br>/CRITERIA=ALPHA(.05)<br>/WSDESIGN=fatigue<br>/DESIGN=groups.
Resources	Processor Time	00:00:00.03
	Elapsed Time	00:00:00.03

Within-Subjects Factors

Measure: MEASURE_1

fatigue	Dependent Variable
1	NbfLRpre
2	NbfLRpos

Between-Subjects Factors

		N
groups	1.00	10
	2.00	16
	3.00	12
	4.00	15

Descriptive Statistics

	groups	Mean	Std. Deviation	N
NbfLRpre	1.00	17.8728	13.10520	10
	2.00	19.1192	8.58687	16
	3.00	13.6337	5.85553	12
	4.00	6.9770	3.78296	15
	Total	14.7315	9.30913	53
NbfLRpos	1.00	19.2323	17.03301	10
	2.00	22.5169	16.28125	16
	3.00	16.5109	6.81087	12
	4.00	6.9459	2.28533	15
	Total	16.8312	13.45199	53

Box's Test of Equality of Covariance Matrices[a]

Box's M	45.983
F	4.663
df1	9
df2	12234.839
Sig.	.000

Tests the null hypothesis that the observed covariance matrices of the dependent variables are equal across groups.
a. Design: Intercept + groups
Within Subjects Design: fatigue

Multivariate Tests[a]

Effect		Value	F	Hypothesis df	Error df	Sig.	Partial Eta Squared	Noncent. Parameter	Observed Power[c]
fatigue	Pillai's Trace	.037	1.693[b]	1.000	44.000	.200	.037	1.693	.247
	Wilks' Lambda	.963	1.693[b]	1.000	44.000	.200	.037	1.693	.247
	Hotelling's Trace	.038	1.693[b]	1.000	44.000	.200	.037	1.693	.247
	Roy's Largest Root	.038	1.693[b]	1.000	44.000	.200	.037	1.693	.247
fatigue * groups	Pillai's Trace	.020	.302[b]	3.000	44.000	.823	.020	.907	.104
	Wilks' Lambda	.980	.302[b]	3.000	44.000	.823	.020	.907	.104

	Hotelling's Trace	.021	.302[b]	3.000	44.000	.823	.020	.907	.104
	Roy's Largest Root	.021	.302[b]	3.000	44.000	.823	.020	.907	.104

a. Design: Intercept + groups
Within Subjects Design: fatigue
b. Exact statistic
c. Computed using alpha = .05

Mauchly's Test of Sphericity[a]

Measure: MEASURE_1

Within Subjects Effect	Mauchly's W	Approx. Chi-Square	df	Sig.	Epsilon[b] Greenhouse-Geisser	Huynh-Feldt	Lower-bound
fatigue	1.000	.000	0	.	1.000	1.000	1.000

Tests the null hypothesis that the error covariance matrix of the orthonormalized transformed dependent variables is proportional to an identity matrix.
a. Design: Intercept + groups
Within Subjects Design: fatigue
b. May be used to adjust the degrees of freedom for the averaged tests of significance. Corrected tests are displayed in the Tests of Within-Subjects Effects table.

Tests of Within-Subjects Effects

Measure: MEASURE_1

Source		Type III Sum of Squares	df	Mean Square	F	Sig.	Partial Eta Squared	Noncent. Parameter	Observed Power[a]
fatigue	Sphericity Assumed	83.099	1	83.099	1.693	.200	.037	1.693	.247
	Greenhouse-Geisser	83.099	1.000	83.099	1.693	.200	.037	1.693	.247
	Huynh-Feldt	83.099	1.000	83.099	1.693	.200	.037	1.693	.247
	Lower-bound	83.099	1.000	83.099	1.693	.200	.037	1.693	.247
fatigue * groups	Sphericity Assumed	44.542	3	14.847	.302	.823	.020	.907	.104
	Greenhouse-Geisser	44.542	3.000	14.847	.302	.823	.020	.907	.104
	Huynh-Feldt	44.542	3.000	14.847	.302	.823	.020	.907	.104
	Lower-bound	44.542	3.000	14.847	.302	.823	.020	.907	.104
Error(fatigue)	Sphericity Assumed	2159.925	44	49.089					
	Greenhouse-Geisser	2159.925	44.000	49.089					
	Huynh-Feldt	2159.925	44.000	49.089					
	Lower-bound	2159.925	44.000	49.089					

a. Computed using alpha = .05

Tests of Within-Subjects Contrasts

Measure: MEASURE_1

Source	fatigue	Type III Sum of Squares	df	Mean Square	F	Sig.	Partial Eta Squared	Noncent. Parameter	Observed Power[a]
fatigue	Linear	83.099	1	83.099	1.693	.200	.037	1.693	.247
fatigue * groups	Linear	44.542	3	14.847	.302	.823	.020	.907	.104
Error(fatigue)	Linear	2159.925	44	49.089					

a. Computed using alpha = .05

Levene's Test of Equality of Error Variances[a]

		Levene Statistic	df1	df2	Sig.
NbfLRpre	Based on Mean	3.350	3	44	.027
	Based on Median	2.906	3	44	.045
	Based on Median and with adjusted df	2.906	3	25.620	.054
	Based on trimmed mean	3.288	3	44	.029
NbfLRpos	Based on Mean	3.099	3	44	.036
	Based on Median	1.699	3	44	.181
	Based on Median and with adjusted df	1.699	3	24.579	.193
	Based on trimmed mean	2.429	3	44	.078

Tests the null hypothesis that the error variance of the dependent variable is equal across groups.

a. Design: Intercept + groups
Within Subjects Design: fatigue

Tests of Between-Subjects Effects

Measure: MEASURE_1
Transformed Variable: Average

Source	Type III Sum of Squares	df	Mean Square	F	Sig.	Partial Eta Squared	Noncent. Parameter	Observed Power[a]
Intercept	21678.614	1	21678.614	123.878	.000	.738	123.878	1.000
groups	2673.486	3	891.162	5.092	.004	.258	15.277	.895
Error	7699.996	44	175.000					

a. Computed using alpha = .05

Parameter Estimates

Dependent Variable	Parameter	B	Std. Error	t	Sig.	95% Confidence Interval Lower Bound	95% Confidence Interval Upper Bound	Partial Eta Squared	Noncent. Parameter	Observed Power[b]
NbfLRpre	Intercept	6.977	2.490	2.802	.008	1.959	11.995	.151	2.802	.783
	[groups=1.00]	10.896	3.712	2.936	.005	3.416	18.376	.164	2.936	.819
	[groups=2.00]	12.142	3.234	3.754	.001	5.624	18.661	.243	3.754	.956
	[groups=3.00]	6.657	3.447	1.931	.060	-.290	13.603	.078	1.931	.472
	[groups=4.00]	0[a]	.	.	.	.	.	.	.	.
NbfLRpos	Intercept	6.946	3.765	1.845	.072	-.641	14.533	.072	1.845	.438
	[groups=1.00]	12.286	5.612	2.189	.034	.976	23.597	.098	2.189	.572
	[groups=2.00]	15.571	4.890	3.184	.003	5.715	25.427	.187	3.184	.876
	[groups=3.00]	9.565	5.212	1.835	.073	-.939	20.069	.071	1.835	.435
	[groups=4.00]	0[a]	.	.	.	.	.	.	.	.

a. This parameter is set to zero because it is redundant.

b. Computed using alpha = .05

Estimated Marginal Means

1. Grand Mean

Measure: MEASURE_1

Mean	Std. Error	95% Confidence Interval Lower Bound	95% Confidence Interval Upper Bound
15.351	1.379	12.571	18.131

2. groups

Estimates

Measure: MEASURE_1

groups	Mean	Std. Error	95% Confidence Interval

			Lower Bound	Upper Bound
1.00	18.553	3.118	12.269	24.837
2.00	20.818	2.339	16.105	25.531
3.00	15.072	2.700	9.630	20.514
4.00	6.961	2.820	1.277	12.646

Pairwise Comparisons

Measure: MEASURE_1

(I) groups	(J) groups	Mean Difference (I-J)	Std. Error	Sig.[b]	95% Confidence Interval for Difference[b] Lower Bound	Upper Bound
1.00	2.00	-2.266	3.898	1.000	-13.034	8.503
	3.00	3.480	4.125	1.000	-7.916	14.876
	4.00	11.591	4.204	.051	-.025	23.207
2.00	1.00	2.266	3.898	1.000	-8.503	13.034
	3.00	5.746	3.572	.689	-4.123	15.615
	4.00	13.857*	3.664	.003	3.734	23.979
3.00	1.00	-3.480	4.125	1.000	-14.876	7.916
	2.00	-5.746	3.572	.689	-15.615	4.123
	4.00	8.111	3.905	.262	-2.677	18.899
4.00	1.00	-11.591	4.204	.051	-23.207	.025
	2.00	-13.857*	3.664	.003	-23.979	-3.734
	3.00	-8.111	3.905	.262	-18.899	2.677

Based on estimated marginal means

*. The mean difference is significant at the .05 level.

b. Adjustment for multiple comparisons: Bonferroni.

Univariate Tests

Measure: MEASURE_1

	Sum of Squares	df	Mean Square	F	Sig.	Partial Eta Squared	Noncent. Parameter	Observed Power[a]
Contrast	1336.743	3	445.581	5.092	.004	.258	15.277	.895
Error	3849.998	44	87.500					

The F tests the effect of groups. This test is based on the linearly independent pairwise comparisons among the estimated marginal means.

a. Computed using alpha = .05

3. fatigue

Estimates

Measure: MEASURE_1

fatigue	Mean	Std. Error	95% Confidence Interval Lower Bound	Upper Bound
1	14.401	1.218	11.947	16.854
2	16.302	1.841	12.591	20.012

Pairwise Comparisons

Measure: MEASURE_1

(I) fatigue	(J) fatigue	Mean Difference (I-J)	Std. Error	Sig.[a]	95% Confidence Interval for Difference[a] Lower Bound	Upper Bound
1	2	-1.901	1.461	.200	-4.845	1.044
2	1	1.901	1.461	.200	-1.044	4.845

Based on estimated marginal means

a. Adjustment for multiple comparisons: Bonferroni.

Multivariate Tests

	Value	F	Hypothesis df	Error df	Sig.	Partial Eta Squared	Noncent. Parameter	Observed Power[b]
Pillai's trace	.037	1.693[a]	1.000	44.000	.200	.037	1.693	.247
Wilks' lambda	.963	1.693[a]	1.000	44.000	.200	.037	1.693	.247
Hotelling's trace	.038	1.693[a]	1.000	44.000	.200	.037	1.693	.247
Roy's largest root	.038	1.693[a]	1.000	44.000	.200	.037	1.693	.247

Each F tests the multivariate effect of fatigue. These tests are based on the linearly independent pairwise comparisons among the estimated marginal means.

a. Exact statistic

b. Computed using alpha = .05

4. groups * fatigue

Measure: MEASURE_1

groups	fatigue	Mean	Std. Error	95% Confidence Interval Lower Bound	Upper Bound
1.00	1	17.873	2.753	12.325	23.420

	2	19.232	4.162	10.844	27.620
2.00	1	19.119	2.064	14.959	23.280
	2	22.517	3.122	16.226	28.808
3.00	1	13.634	2.384	8.830	18.438
	2	16.511	3.604	9.247	23.775
4.00	1	6.977	2.490	1.959	11.995
	2	6.946	3.765	-.641	14.533

Post Hoc Tests

groups

Multiple Comparisons

Measure: MEASURE_1
Tukey HSD

(I) groups	(J) groups	Mean Difference (I-J)	Std. Error	Sig.	95% Confidence Interval Lower Bound	Upper Bound
1.00	2.00	-2.2655	3.89756	.937	-12.6720	8.1410
	3.00	3.4802	4.12479	.833	-7.5330	14.4934
	4.00	11.5911*	4.20437	.041	.3654	22.8168
2.00	1.00	2.2655	3.89756	.937	-8.1410	12.6720
	3.00	5.7458	3.57217	.384	-3.7920	15.2835
	4.00	13.8566*	3.66378	.003	4.0743	23.6389
3.00	1.00	-3.4802	4.12479	.833	-14.4934	7.5330
	2.00	-5.7458	3.57217	.384	-15.2835	3.7920
	4.00	8.1109	3.90464	.176	-2.3145	18.5363
4.00	1.00	-11.5911*	4.20437	.041	-22.8168	-.3654
	2.00	-13.8566*	3.66378	.003	-23.6389	-4.0743
	3.00	-8.1109	3.90464	.176	-18.5363	2.3145

Based on observed means.
The error term is Mean Square(Error) = 87.500.

*. The mean difference is significant at the .05 level.

Homogeneous Subsets

MEASURE_1

Tukey HSD[a,b,c]

groups	N	Subset 1	Subset 2
4.00	15	6.9614	
3.00	12	15.0723	15.0723
1.00	10		18.5525
2.00	16		20.8181
Sig.		.176	.462

Means for groups in homogeneous subsets are displayed.
Based on observed means.
The error term is Mean Square(Error) = 87.500.
a. Uses Harmonic Mean Sample Size = 11.499.
b. The group sizes are unequal. The harmonic mean of the group sizes is used. Type I error levels are not guaranteed.
c. Alpha = .05.

Semitendinosus (ST) - Early Stance

```
GLM NstLRpre NstLRpos BY groups
  /WSFACTOR=fatigue 2 Polynomial
  /METHOD=SSTYPE(3)
  /POSTHOC=groups(TUKEY)
  /EMMEANS=TABLES(OVERALL)
  /EMMEANS=TABLES(groups) COMPARE ADJ(BONFERRONI)
  /EMMEANS=TABLES(fatigue) COMPARE ADJ(BONFERRONI)
  /EMMEANS=TABLES(groups*fatigue)
  /PRINT=DESCRIPTIVE ETASQ OPOWER PARAMETER HOMOGENEITY
  /CRITERIA=ALPHA(.05)
  /WSDESIGN=fatigue
  /DESIGN=groups.
```

General Linear Model

Notes

Output Created		17-MAY-2026 21:28:16
Comments		
Input	Data	C:\Users\RaSa\Desk-top\پروپوزال\New folder\data\emgmi1.sav
	Active Dataset	DataSet2
	Filter	<none>
	Weight	<none>
	Split File	<none>
	N of Rows in Working Data File	48
Missing Value Handling	Definition of Missing	User-defined missing values are treated as missing.
	Cases Used	Statistics are based on all cases with valid data for all variables in the model.

Syntax		GLM NstLRpre NstLRpos BY groups<br>/WSFACTOR=fatigue 2 Polynomial<br>/METHOD=SSTYPE(3)<br>/POSTHOC=groups(TUKEY)<br>/EMMEANS=TABLES(OVERALL)<br>/EMMEANS=TABLES(groups) COMPARE ADJ(BONFERRONI)<br>/EMMEANS=TABLES(fatigue) COMPARE ADJ(BONFERRONI)<br>/EMMEANS=TABLES(groups*fatigue)<br>/PRINT=DESCRIPTIVE ETASQ OPOWER PARAMETER HOMOGENEITY<br>/CRITERIA=ALPHA(.05)<br>/WSDESIGN=fatigue<br>/DESIGN=groups.
Resources	Processor Time	00:00:00.03
	Elapsed Time	00:00:00.03

Within-Subjects Factors

Measure: MEASURE_1

fatigue	Dependent Variable
1	NstLRpre
2	NstLRpos

Between-Subjects Factors

		N
groups	1.00	10
	2.00	16
	3.00	12
	4.00	15

Descriptive Statistics

	groups	Mean	Std. Deviation	N
NstLRpre	1.00	22.2962	10.74389	10
	2.00	21.1781	13.06886	16
	3.00	21.9299	15.78402	12
	4.00	8.4763	6.92382	15
	Total	18.6649	13.20444	53
NstLRpos	1.00	17.9444	10.98195	10
	2.00	18.4063	8.96121	16
	3.00	16.5733	7.52385	12
	4.00	6.2622	2.87971	15
	Total	15.0784	9.23444	53

Box's Test of Equality of Covariance Matrices[a]

Box's M	31.003
F	3.144
df1	9
df2	12234.839
Sig.	.001

Tests the null hypothesis that the observed covariance matrices of the dependent variables are equal across groups.

a. Design: Intercept + groups
Within Subjects Design: fatigue

Multivariate Tests[a]

Effect		Value	F	Hypothesis df	Error df	Sig.	Partial Eta Squared	Noncent. Parameter	Observed Power[c]
fatigue	Pillai's Trace	.091	4.393[b]	1.000	44.000	.042	.091	4.393	.536
	Wilks' Lambda	.909	4.393[b]	1.000	44.000	.042	.091	4.393	.536
	Hotelling's Trace	.100	4.393[b]	1.000	44.000	.042	.091	4.393	.536
	Roy's Largest Root	.100	4.393[b]	1.000	44.000	.042	.091	4.393	.536
fatigue * groups	Pillai's Trace	.012	.175[b]	3.000	44.000	.913	.012	.525	.080
	Wilks' Lambda	.988	.175[b]	3.000	44.000	.913	.012	.525	.080

	Hotelling's Trace	.012	.175[b]	3.000	44.000	.913	.012	.525	.080
	Roy's Largest Root	.012	.175[b]	3.000	44.000	.913	.012	.525	.080

a. Design: Intercept + groups
Within Subjects Design: fatigue
b. Exact statistic
c. Computed using alpha = .05

Mauchly's Test of Sphericity[a]

Measure: MEASURE_1

Within Subjects Effect	Mauchly's W	Approx. Chi-Square	df	Sig.	Epsilon[b] Greenhouse-Geisser	Epsilon[b] Huynh-Feldt	Epsilon[b] Lower-bound
fatigue	1.000	.000	0	.	1.000	1.000	1.000

Tests the null hypothesis that the error covariance matrix of the orthonormalized transformed dependent variables is proportional to an identity matrix.
a. Design: Intercept + groups
Within Subjects Design: fatigue
b. May be used to adjust the degrees of freedom for the averaged tests of significance. Corrected tests are displayed in the Tests of Within-Subjects Effects table.

Tests of Within-Subjects Effects

Measure: MEASURE_1

Source		Type III Sum of Squares	df	Mean Square	F	Sig.	Partial Eta Squared	Noncent. Parameter	Observed Power[a]
fatigue	Sphericity Assumed	310.364	1	310.364	4.393	.042	.091	4.393	.536
	Greenhouse-Geisser	310.364	1.000	310.364	4.393	.042	.091	4.393	.536
	Huynh-Feldt	310.364	1.000	310.364	4.393	.042	.091	4.393	.536
	Lower-bound	310.364	1.000	310.364	4.393	.042	.091	4.393	.536
fatigue * groups	Sphericity Assumed	37.104	3	12.368	.175	.913	.012	.525	.080
	Greenhouse-Geisser	37.104	3.000	12.368	.175	.913	.012	.525	.080
	Huynh-Feldt	37.104	3.000	12.368	.175	.913	.012	.525	.080
	Lower-bound	37.104	3.000	12.368	.175	.913	.012	.525	.080
Error(fatigue)	Sphericity Assumed	3108.400	44	70.645					
	Greenhouse-Geisser	3108.400	44.000	70.645					
	Huynh-Feldt	3108.400	44.000	70.645					
	Lower-bound	3108.400	44.000	70.645					

a. Computed using alpha = .05

Tests of Within-Subjects Contrasts

Measure: MEASURE_1

Source	fatigue	Type III Sum of Squares	df	Mean Square	F	Sig.	Partial Eta Squared	Noncent. Parameter	Observed Power[a]
fatigue	Linear	310.364	1	310.364	4.393	.042	.091	4.393	.536
fatigue * groups	Linear	37.104	3	12.368	.175	.913	.012	.525	.080
Error(fatigue)	Linear	3108.400	44	70.645					

a. Computed using alpha = .05

Levene's Test of Equality of Error Variances[a]

		Levene Statistic	df1	df2	Sig.
NstLRpre	Based on Mean	1.497	3	44	.229
	Based on Median	1.604	3	44	.202
	Based on Median and with adjusted df	1.604	3	34.840	.206
	Based on trimmed mean	1.527	3	44	.221
NstLRpos	Based on Mean	4.460	3	44	.008
	Based on Median	3.774	3	44	.017
	Based on Median and with adjusted df	3.774	3	29.609	.021
	Based on trimmed mean	4.352	3	44	.009

Tests the null hypothesis that the error variance of the dependent variable is equal across groups.

a. Design: Intercept + groups
Within Subjects Design: fatigue

Tests of Between-Subjects Effects

Measure: MEASURE_1
Transformed Variable: Average

Source	Type III Sum of Squares	df	Mean Square	F	Sig.	Partial Eta Squared	Noncent. Parameter	Observed Power[a]
Intercept	25451.477	1	25451.477	173.036	.000	.797	173.036	1.000
groups	2585.359	3	861.786	5.859	.002	.285	17.577	.935
Error	6471.849	44	147.087					

a. Computed using alpha = .05

Parameter Estimates

Dependent Variable	Parameter	B	Std. Error	t	Sig.	95% Confidence Interval		Partial Eta Squared	Noncent. Parameter	Observed Power[b]
						Lower Bound	Upper Bound			
NstLRpre	Intercept	8.476	3.722	2.277	.028	.975	15.978	.105	2.277	.605
	[groups=1.00]	13.820	5.549	2.491	.017	2.638	25.002	.124	2.491	.683
	[groups=2.00]	12.702	4.835	2.627	.012	2.957	22.446	.136	2.627	.729
	[groups=3.00]	13.454	5.153	2.611	.012	3.068	23.839	.134	2.611	.724
	[groups=4.00]	0[a]	.	.	.	.	.	.	.	.
NstLRpos	Intercept	6.262	2.437	2.569	.014	1.350	11.174	.130	2.569	.710
	[groups=1.00]	11.682	3.633	3.215	.002	4.360	19.004	.190	3.215	.882
	[groups=2.00]	12.144	3.166	3.836	.000	5.763	18.525	.251	3.836	.963
	[groups=3.00]	10.311	3.374	3.056	.004	3.511	17.111	.175	3.056	.848
	[groups=4.00]	0[a]	.	.	.	.	.	.	.	.

a. This parameter is set to zero because it is redundant.

b. Computed using alpha = .05

Estimated Marginal Means

1. Grand Mean

Measure: MEASURE_1

Mean	Std. Error	95% Confidence Interval	
		Lower Bound	Upper Bound
16.633	1.264	14.085	19.182

2. groups

Estimates

Measure: MEASURE_1

groups	Mean	Std. Error	95% Confidence Interval

			Lower Bound	Upper Bound
1.00	20.120	2.859	14.359	25.881
2.00	19.792	2.144	15.471	24.113
3.00	19.252	2.476	14.262	24.241
4.00	7.369	2.586	2.158	12.580

Pairwise Comparisons

Measure: MEASURE_1

(I) groups	(J) groups	Mean Difference (I-J)	Std. Error	Sig.[b]	95% Confidence Interval for Difference[b] Lower Bound	Upper Bound
1.00	2.00	.328	3.573	1.000	-9.544	10.200
	3.00	.869	3.782	1.000	-9.579	11.316
	4.00	12.751*	3.855	.011	2.102	23.400
2.00	1.00	-.328	3.573	1.000	-10.200	9.544
	3.00	.541	3.275	1.000	-8.507	9.589
	4.00	12.423*	3.359	.004	3.143	21.703
3.00	1.00	-.869	3.782	1.000	-11.316	9.579
	2.00	-.541	3.275	1.000	-9.589	8.507
	4.00	11.882*	3.580	.011	1.992	21.772
4.00	1.00	-12.751*	3.855	.011	-23.400	-2.102
	2.00	-12.423*	3.359	.004	-21.703	-3.143
	3.00	-11.882*	3.580	.011	-21.772	-1.992

Based on estimated marginal means

*. The mean difference is significant at the .05 level.

b. Adjustment for multiple comparisons: Bonferroni.

Univariate Tests

Measure: MEASURE_1

	Sum of Squares	df	Mean Square	F	Sig.	Partial Eta Squared	Noncent. Parameter	Observed Power[a]
Contrast	1292.680	3	430.893	5.859	.002	.285	17.577	.935
Error	3235.924	44	73.544					

The F tests the effect of groups. This test is based on the linearly independent pairwise comparisons among the estimated marginal means.

a. Computed using alpha = .05

3. fatigue

Estimates

Measure: MEASURE_1

fatigue	Mean	Std. Error	95% Confidence Interval Lower Bound	95% Confidence Interval Upper Bound
1	18.470	1.820	14.802	22.139
2	14.797	1.192	12.394	17.199

Pairwise Comparisons

Measure: MEASURE_1

(I) fatigue	(J) fatigue	Mean Difference (I-J)	Std. Error	Sig.[b]	95% Confidence Interval for Difference[b] Lower Bound	95% Confidence Interval for Difference[b] Upper Bound
1	2	3.674*	1.753	.042	.141	7.206
2	1	-3.674*	1.753	.042	-7.206	-.141

Based on estimated marginal means

*. The mean difference is significant at the .05 level.

b. Adjustment for multiple comparisons: Bonferroni.

Multivariate Tests

	Value	F	Hypothesis df	Error df	Sig.	Partial Eta Squared	Noncent. Parameter	Observed Power[b]
Pillai's trace	.091	4.393[a]	1.000	44.000	.042	.091	4.393	.536
Wilks' lambda	.909	4.393[a]	1.000	44.000	.042	.091	4.393	.536
Hotelling's trace	.100	4.393[a]	1.000	44.000	.042	.091	4.393	.536
Roy's largest root	.100	4.393[a]	1.000	44.000	.042	.091	4.393	.536

Each F tests the multivariate effect of fatigue. These tests are based on the linearly independent pairwise comparisons among the estimated marginal means.

a. Exact statistic

b. Computed using alpha = .05

4. groups * fatigue

Measure: MEASURE_1

groups	fatigue	Mean	Std. Error	95% Confidence Interval Lower Bound	95% Confidence Interval Upper Bound

1.00	1	22.296	4.115	14.003	30.589
	2	17.944	2.694	12.514	23.375
2.00	1	21.178	3.086	14.958	27.398
	2	18.406	2.021	14.334	22.479
3.00	1	21.930	3.564	14.748	29.112
	2	16.573	2.333	11.871	21.276
4.00	1	8.476	3.722	.975	15.978
	2	6.262	2.437	1.350	11.174

Post Hoc Tests

groups

Multiple Comparisons

Measure: MEASURE_1
Tukey HSD

(I) groups	(J) groups	Mean Difference (I-J)	Std. Error	Sig.	95% Confidence Interval Lower Bound	Upper Bound
1.00	2.00	.3281	3.57324	1.000	-9.2124	9.8687
	3.00	.8687	3.78156	.996	-9.2280	10.9655
	4.00	12.7511*	3.85452	.010	2.4595	23.0427
2.00	1.00	-.3281	3.57324	1.000	-9.8687	9.2124
	3.00	.5406	3.27492	.998	-8.2035	9.2847
	4.00	12.4229*	3.35891	.003	3.4546	21.3913
3.00	1.00	-.8687	3.78156	.996	-10.9655	9.2280
	2.00	-.5406	3.27492	.998	-9.2847	8.2035
	4.00	11.8823*	3.57973	.009	2.3244	21.4402
4.00	1.00	-12.7511*	3.85452	.010	-23.0427	-2.4595
	2.00	-12.4229*	3.35891	.003	-21.3913	-3.4546
	3.00	-11.8823*	3.57973	.009	-21.4402	-2.3244

Based on observed means.
The error term is Mean Square(Error) = 73.544.
*. The mean difference is significant at the .05 level.

Homogeneous Subsets

MEASURE_1

Tukey HSD[a,b,c]

groups	N	Subset 1	Subset 2
4.00	15	7.3693	
3.00	12		19.2516
2.00	16		19.7922
1.00	10		20.1203
Sig.		1.000	.995

Means for groups in homogeneous subsets are displayed.
Based on observed means.
The error term is Mean Square(Error) = 73.544.
a. Uses Harmonic Mean Sample Size = 11.499.
b. The group sizes are unequal. The harmonic mean of the group sizes is used. Type I error levels are not guaranteed.
c. Alpha = .05.

Gluteus Medius (GM) - Early Stance

```
GLM NgmLRpre NgmLRpos BY groups
  /WSFACTOR=fatigue 2 Polynomial
  /METHOD=SSTYPE(3)
  /POSTHOC=groups(TUKEY)
  /EMMEANS=TABLES(OVERALL)
  /EMMEANS=TABLES(groups) COMPARE ADJ(BONFERRONI)
  /EMMEANS=TABLES(fatigue) COMPARE ADJ(BONFERRONI)
  /EMMEANS=TABLES(groups*fatigue)
  /PRINT=DESCRIPTIVE ETASQ OPOWER PARAMETER HOMOGENEITY
  /CRITERIA=ALPHA(.05)
  /WSDESIGN=fatigue
  /DESIGN=groups.
```

General Linear Model

Notes

Output Created		17-MAY-2026 21:28:36
Comments		
Input	Data	C:\Users\RaSa\Desk-top\پروپوزال\New folder\data\emgmi1.sav
	Active Dataset	DataSet2
	Filter	<none>
	Weight	<none>
	Split File	<none>
	N of Rows in Working Data File	48
Missing Value Handling	Definition of Missing	User-defined missing values are treated as missing.
	Cases Used	Statistics are based on all cases with valid data for all variables in the model.

Syntax		GLM NgmLRpre NgmLRpos BY groups<br>/WSFACTOR=fatigue 2 Polynomial<br>/METHOD=SSTYPE(3)<br>/POSTHOC=groups(TUKEY)<br>/EMMEANS=TABLES(OVERALL)<br>/EMMEANS=TABLES(groups) COMPARE ADJ(BONFERRONI)<br>/EMMEANS=TABLES(fatigue) COMPARE ADJ(BONFERRONI)<br>/EMMEANS=TABLES(groups*fatigue)<br>/PRINT=DESCRIPTIVE ETASQ OPOWER PARAMETER HOMOGENEITY<br>/CRITERIA=ALPHA(.05)<br>/WSDESIGN=fatigue<br>/DESIGN=groups.
Resources	Processor Time	00:00:00.03
	Elapsed Time	00:00:00.03

Within-Subjects Factors

Measure: MEASURE_1

fatigue	Dependent Variable
1	NgmLRpre
2	NgmLRpos

Between-Subjects Factors

		N
groups	1.00	10
	2.00	16
	3.00	12
	4.00	15

Descriptive Statistics

	groups	Mean	Std. Deviation	N
NgmLRpre	1.00	24.7244	12.95172	10
	2.00	23.2299	13.31438	16
	3.00	18.2594	13.36672	12
	4.00	10.7101	4.96063	15
	Total	19.3984	12.67315	53
NgmLRpos	1.00	8.1639	4.17914	10
	2.00	11.0940	6.57498	16
	3.00	9.4492	5.22427	12
	4.00	5.8220	4.23223	15
	Total	8.9252	5.56816	53

Box's Test of Equality of Covariance Matrices[a]

Box's M	23.074
F	2.340
df1	9
df2	12234.839
Sig.	.012

Tests the null hypothesis that the observed covariance matrices of the dependent variables are equal across groups.

a. Design: Intercept + groups
Within Subjects Design: fatigue

Multivariate Tests[a]

Effect		Value	F	Hypothesis df	Error df	Sig.	Partial Eta Squared	Noncent. Parameter	Observed Power[c]
fatigue	Pillai's Trace	.442	34.885[b]	1.000	44.000	.000	.442	34.885	1.000
	Wilks' Lambda	.558	34.885[b]	1.000	44.000	.000	.442	34.885	1.000
	Hotelling's Trace	.793	34.885[b]	1.000	44.000	.000	.442	34.885	1.000
	Roy's Largest Root	.793	34.885[b]	1.000	44.000	.000	.442	34.885	1.000
fatigue * groups	Pillai's Trace	.104	1.697[b]	3.000	44.000	.181	.104	5.091	.413
	Wilks' Lambda	.896	1.697[b]	3.000	44.000	.181	.104	5.091	.413

	Hotelling's Trace	.116	1.697[b]	3.000	44.000	.181	.104	5.091	.413
	Roy's Largest Root	.116	1.697[b]	3.000	44.000	.181	.104	5.091	.413

a. Design: Intercept + groups
 Within Subjects Design: fatigue
b. Exact statistic
c. Computed using alpha = .05

Mauchly's Test of Sphericity[a]

Measure: MEASURE_1

Within Subjects Effect	Mauchly's W	Approx. Chi-Square	df	Sig.	Epsilon[b]		
					Greenhouse-Geisser	Huynh-Feldt	Lower-bound
fatigue	1.000	.000	0	.	1.000	1.000	1.000

Tests the null hypothesis that the error covariance matrix of the orthonormalized transformed dependent variables is proportional to an identity matrix.
a. Design: Intercept + groups
 Within Subjects Design: fatigue
b. May be used to adjust the degrees of freedom for the averaged tests of significance. Corrected tests are displayed in the Tests of Within-Subjects Effects table.

Tests of Within-Subjects Effects

Measure: MEASURE_1

Source		Type III Sum of Squares	df	Mean Square	F	Sig.	Partial Eta Squared	Noncent. Parameter	Observed Power[a]
fatigue	Sphericity Assumed	2583.433	1	2583.433	34.885	.000	.442	34.885	1.000
	Greenhouse-Geisser	2583.433	1.000	2583.433	34.885	.000	.442	34.885	1.000
	Huynh-Feldt	2583.433	1.000	2583.433	34.885	.000	.442	34.885	1.000
	Lower-bound	2583.433	1.000	2583.433	34.885	.000	.442	34.885	1.000
fatigue * groups	Sphericity Assumed	377.025	3	125.675	1.697	.181	.104	5.091	.413
	Greenhouse-Geisser	377.025	3.000	125.675	1.697	.181	.104	5.091	.413
	Huynh-Feldt	377.025	3.000	125.675	1.697	.181	.104	5.091	.413
	Lower-bound	377.025	3.000	125.675	1.697	.181	.104	5.091	.413
Error(fatigue)	Sphericity Assumed	3258.469	44	74.056					
	Greenhouse-Geisser	3258.469	44.000	74.056					
	Huynh-Feldt	3258.469	44.000	74.056					
	Lower-bound	3258.469	44.000	74.056					

a. Computed using alpha = .05

Tests of Within-Subjects Contrasts

Measure: MEASURE_1

Source	fatigue	Type III Sum of Squares	df	Mean Square	F	Sig.	Partial Eta Squared	Noncent. Parameter	Observed Power[a]
fatigue	Linear	2583.433	1	2583.433	34.885	.000	.442	34.885	1.000
fatigue * groups	Linear	377.025	3	125.675	1.697	.181	.104	5.091	.413
Error(fatigue)	Linear	3258.469	44	74.056					

a. Computed using alpha = .05

Levene's Test of Equality of Error Variances[a]

		Levene Statistic	df1	df2	Sig.
NgmLRpre	Based on Mean	2.319	3	44	.088
	Based on Median	1.886	3	44	.146
	Based on Median and with adjusted df	1.886	3	36.293	.149
	Based on trimmed mean	2.250	3	44	.096
NgmLRpos	Based on Mean	2.759	3	44	.053
	Based on Median	2.551	3	44	.068
	Based on Median and with adjusted df	2.551	3	42.781	.068
	Based on trimmed mean	2.747	3	44	.054

Tests the null hypothesis that the error variance of the dependent variable is equal across groups.

a. Design: Intercept + groups

Within Subjects Design: fatigue

Tests of Between-Subjects Effects

Measure: MEASURE_1

Transformed Variable: Average

Source	Type III Sum of Squares	df	Mean Square	F	Sig.	Partial Eta Squared	Noncent. Parameter	Observed Power[a]
Intercept	17854.832	1	17854.832	186.096	.000	.809	186.096	1.000
groups	1148.772	3	382.924	3.991	.013	.214	11.973	.802
Error	4221.554	44	95.944					

a. Computed using alpha = .05

Parameter Estimates

Dependent Variable	Parameter	B	Std. Error	t	Sig.	95% Confidence Interval		Partial Eta Squared	Noncent. Parameter	Observed Power[b]
						Lower Bound	Upper Bound			
NgmLRpre	Intercept	10.710	3.583	2.989	.005	3.490	17.931	.169	2.989	.832
	[groups=1.00]	14.014	5.341	2.624	.012	3.251	24.778	.135	2.624	.728
	[groups=2.00]	12.520	4.654	2.690	.010	3.140	21.899	.141	2.690	.749
	[groups=3.00]	7.549	4.960	1.522	.135	-2.447	17.546	.050	1.522	.319
	[groups=4.00]	0[a]	.	.	.	.	.	.	.	.
NgmLRpos	Intercept	5.822	1.618	3.598	.001	2.561	9.083	.227	3.598	.940
	[groups=1.00]	2.342	2.412	.971	.337	-2.520	7.204	.021	.971	.158
	[groups=2.00]	5.272	2.102	2.508	.016	1.035	9.509	.125	2.508	.689
	[groups=3.00]	3.627	2.240	1.619	.113	-.888	8.142	.056	1.619	.354
	[groups=4.00]	0[a]	.	.	.	.	.	.	.	.

a. This parameter is set to zero because it is redundant.

b. Computed using alpha = .05

Estimated Marginal Means

1. Grand Mean

Measure: MEASURE_1

Mean	Std. Error	95% Confidence Interval	
		Lower Bound	Upper Bound
13.932	1.021	11.873	15.990

2. groups

Estimates

Measure: MEASURE_1

groups	Mean	Std. Error	95% Confidence Interval

			Lower Bound	Upper Bound
1.00	16.444	2.309	11.791	21.097
2.00	17.162	1.732	13.672	20.652
3.00	13.854	1.999	9.825	17.884
4.00	8.266	2.088	4.057	12.475

Pairwise Comparisons

Measure: MEASURE_1

(I) groups	(J) groups	Mean Difference (I-J)	Std. Error	Sig.[b]	95% Confidence Interval for Difference[b] Lower Bound	Upper Bound
1.00	2.00	-.718	2.886	1.000	-8.691	7.255
	3.00	2.590	3.054	1.000	-5.848	11.028
	4.00	8.178	3.113	.071	-.423	16.779
2.00	1.00	.718	2.886	1.000	-7.255	8.691
	3.00	3.308	2.645	1.000	-4.000	10.615
	4.00	8.896*	2.713	.012	1.401	16.391
3.00	1.00	-2.590	3.054	1.000	-11.028	5.848
	2.00	-3.308	2.645	1.000	-10.615	4.000
	4.00	5.588	2.891	.358	-2.399	13.576
4.00	1.00	-8.178	3.113	.071	-16.779	.423
	2.00	-8.896*	2.713	.012	-16.391	-1.401
	3.00	-5.588	2.891	.358	-13.576	2.399

Based on estimated marginal means

*. The mean difference is significant at the .05 level.

b. Adjustment for multiple comparisons: Bonferroni.

Univariate Tests

Measure: MEASURE_1

	Sum of Squares	df	Mean Square	F	Sig.	Partial Eta Squared	Noncent. Parameter	Observed Power[a]
Contrast	574.386	3	191.462	3.991	.013	.214	11.973	.802
Error	2110.777	44	47.972					

The F tests the effect of groups. This test is based on the linearly independent pairwise comparisons among the estimated marginal means.

a. Computed using alpha = .05

3. fatigue

Estimates

Measure: MEASURE_1

fatigue	Mean	Std. Error	95% Confidence Interval Lower Bound	95% Confidence Interval Upper Bound
1	19.231	1.752	15.700	22.762
2	8.632	.791	7.037	10.227

Pairwise Comparisons

Measure: MEASURE_1

(I) fatigue	(J) fatigue	Mean Difference (I-J)	Std. Error	Sig.[b]	95% Confidence Interval for Difference[b] Lower Bound	95% Confidence Interval for Difference[b] Upper Bound
1	2	10.599*	1.794	.000	6.982	14.215
2	1	-10.599*	1.794	.000	-14.215	-6.982

Based on estimated marginal means

*. The mean difference is significant at the .05 level.

b. Adjustment for multiple comparisons: Bonferroni.

Multivariate Tests

	Value	F	Hypothesis df	Error df	Sig.	Partial Eta Squared	Noncent. Parameter	Observed Power[b]
Pillai's trace	.442	34.885[a]	1.000	44.000	.000	.442	34.885	1.000
Wilks' lambda	.558	34.885[a]	1.000	44.000	.000	.442	34.885	1.000
Hotelling's trace	.793	34.885[a]	1.000	44.000	.000	.442	34.885	1.000
Roy's largest root	.793	34.885[a]	1.000	44.000	.000	.442	34.885	1.000

Each F tests the multivariate effect of fatigue. These tests are based on the linearly independent pairwise comparisons among the estimated marginal means.

a. Exact statistic

b. Computed using alpha = .05

4. groups * fatigue

Measure: MEASURE_1

groups	fatigue	Mean	Std. Error	95% Confidence Interval Lower Bound	95% Confidence Interval Upper Bound

1.00	1	24.724	3.961	16.742	32.707
	2	8.164	1.789	4.558	11.770
2.00	1	23.230	2.971	17.243	29.217
	2	11.094	1.342	8.390	13.798
3.00	1	18.259	3.430	11.346	25.172
	2	9.449	1.549	6.327	12.572
4.00	1	10.710	3.583	3.490	17.931
	2	5.822	1.618	2.561	9.083

Post Hoc Tests

groups

Multiple Comparisons

Measure: MEASURE_1
Tukey HSD

(I) groups	(J) groups	Mean Difference (I-J)	Std. Error	Sig.	95% Confidence Interval Lower Bound	Upper Bound
1.00	2.00	-.7178	2.88592	.995	-8.4232	6.9877
	3.00	2.5899	3.05417	.831	-5.5648	10.7445
	4.00	8.1781	3.11309	.055	-.1339	16.4901
2.00	1.00	.7178	2.88592	.995	-6.9877	8.4232
	3.00	3.3076	2.64499	.599	-3.7545	10.3697
	4.00	8.8959*	2.71282	.011	1.6526	16.1391
3.00	1.00	-2.5899	3.05417	.831	-10.7445	5.5648
	2.00	-3.3076	2.64499	.599	-10.3697	3.7545
	4.00	5.5882	2.89116	.229	-2.1312	13.3077
4.00	1.00	-8.1781	3.11309	.055	-16.4901	.1339
	2.00	-8.8959*	2.71282	.011	-16.1391	-1.6526
	3.00	-5.5882	2.89116	.229	-13.3077	2.1312

Based on observed means.
The error term is Mean Square(Error) = 47.972.
*. The mean difference is significant at the .05 level.

Homogeneous Subsets

MEASURE_1

Tukey HSD[a,b,c]

groups	N	Subset	
		1	2
4.00	15	8.2661	
3.00	12	13.8543	13.8543
1.00	10		16.4442
2.00	16		17.1619
Sig.		.229	.664

Means for groups in homogeneous subsets are displayed.
Based on observed means.
The error term is Mean Square(Error) = 47.972.
a. Uses Harmonic Mean Sample Size = 11.499.
b. The group sizes are unequal. The harmonic mean of the group sizes is used. Type I error levels are not guaranteed.
c. Alpha = .05.

Section 2: Late Stance Phase - EMG Amplitude Outputs

2.1. Tibialis Anterior (TA) - Late Stance

```
GLM NtaPOpre NtaPOpos BY groups
  /WSFACTOR=fatigue 2 Polynomial
  /METHOD=SSTYPE(3)
  /POSTHOC=groups(TUKEY)
  /EMMEANS=TABLES(OVERALL)
  /EMMEANS=TABLES(groups) COMPARE ADJ(BONFERRONI)
  /EMMEANS=TABLES(fatigue) COMPARE ADJ(BONFERRONI)
  /EMMEANS=TABLES(groups*fatigue)
  /PRINT=DESCRIPTIVE ETASQ OPOWER PARAMETER HOMOGENEITY
  /CRITERIA=ALPHA(.05)
  /WSDESIGN=fatigue
  /DESIGN=groups.
```

General Linear Model

Notes

Output Created		17-MAY-2026 21:29:15
Comments		
Input	Data	C:\Users\RaSa\Desktop\پروپوزال\New folder\data\emgmi1.sav
	Active Dataset	DataSet2
	Filter	<none>
	Weight	<none>
	Split File	<none>
	N of Rows in Working Data File	48
Missing Value Handling	Definition of Missing	User-defined missing values are treated as missing.
	Cases Used	Statistics are based on all cases with valid data for all variables in the model.

Syntax		GLM NtaPOpre NtaPOpos BY groups<br>/WSFACTOR=fatigue 2 Polynomial<br>/METHOD=SSTYPE(3)<br>/POSTHOC=groups(TUKEY)<br>/EMMEANS=TABLES(OVERALL)<br>/EMMEANS=TABLES(groups) COMPARE ADJ(BONFERRONI)<br>/EMMEANS=TABLES(fatigue) COMPARE ADJ(BONFERRONI)<br>/EMMEANS=TABLES(groups*fatigue)<br>/PRINT=DESCRIPTIVE ETASQ OPOWER PARAMETER HOMOGENEITY<br>/CRITERIA=ALPHA(.05)<br>/WSDESIGN=fatigue<br>/DESIGN=groups.
Resources	Processor Time	00:00:00.03
	Elapsed Time	00:00:00.03

Within-Subjects Factors

Measure: MEASURE_1

fatigue	Dependent Variable
1	NtaPOpre
2	NtaPOpos

Between-Subjects Factors

		N
groups	1.00	10
	2.00	16
	3.00	12
	4.00	15

Descriptive Statistics

	groups	Mean	Std. Deviation	N
NtaPOpre	1.00	12.6963	7.86558	10
	2.00	6.7152	3.23213	16
	3.00	6.8873	2.46219	12
	4.00	6.2150	2.17271	15
	Total	7.7651	4.69879	53
NtaPOpos	1.00	10.2457	5.66454	10
	2.00	6.8104	2.30615	16
	3.00	7.3036	3.03460	12
	4.00	6.9876	2.44840	15
	Total	7.6184	3.50030	53

Box's Test of Equality of Covariance Matrices[a]

Box's M	35.598
F	3.610
df1	9
df2	12234.839
Sig.	.000

Tests the null hypothesis that the observed covariance matrices of the dependent variables are equal across groups.

a. Design: Intercept + groups
Within Subjects Design: fatigue

Multivariate Tests[a]

Effect		Value	F	Hypothesis df	Error df	Sig.	Partial Eta Squared	Noncent. Parameter	Observed Power[c]
fatigue	Pillai's Trace	.005	.229[b]	1.000	44.000	.635	.005	.229	.075
	Wilks' Lambda	.995	.229[b]	1.000	44.000	.635	.005	.229	.075
	Hotelling's Trace	.005	.229[b]	1.000	44.000	.635	.005	.229	.075
	Roy's Largest Root	.005	.229[b]	1.000	44.000	.635	.005	.229	.075
fatigue * groups	Pillai's Trace	.076	1.204[b]	3.000	44.000	.320	.076	3.611	.300
	Wilks' Lambda	.924	1.204[b]	3.000	44.000	.320	.076	3.611	.300

	Hotelling's Trace	.082	1.204[b]	3.000	44.000	.320	.076	3.611	.300
	Roy's Largest Root	.082	1.204[b]	3.000	44.000	.320	.076	3.611	.300

a. Design: Intercept + groups
Within Subjects Design: fatigue
b. Exact statistic
c. Computed using alpha = .05

Mauchly's Test of Sphericity[a]

Measure: MEASURE_1

Within Subjects Effect	Mauchly's W	Approx. Chi-Square	df	Sig.	Epsilon[b] Greenhouse-Geisser	Huynh-Feldt	Lower-bound
fatigue	1.000	.000	0	.	1.000	1.000	1.000

Tests the null hypothesis that the error covariance matrix of the orthonormalized transformed dependent variables is proportional to an identity matrix.
a. Design: Intercept + groups
Within Subjects Design: fatigue
b. May be used to adjust the degrees of freedom for the averaged tests of significance. Corrected tests are displayed in the Tests of Within-Subjects Effects table.

Tests of Within-Subjects Effects

Measure: MEASURE_1

Source		Type III Sum of Squares	df	Mean Square	F	Sig.	Partial Eta Squared	Noncent. Parameter	Observed Power[a]
fatigue	Sphericity Assumed	1.956	1	1.956	.229	.635	.005	.229	.075
	Greenhouse-Geisser	1.956	1.000	1.956	.229	.635	.005	.229	.075
	Huynh-Feldt	1.956	1.000	1.956	.229	.635	.005	.229	.075
	Lower-bound	1.956	1.000	1.956	.229	.635	.005	.229	.075
fatigue * groups	Sphericity Assumed	30.904	3	10.301	1.204	.320	.076	3.611	.300
	Greenhouse-Geisser	30.904	3.000	10.301	1.204	.320	.076	3.611	.300
	Huynh-Feldt	30.904	3.000	10.301	1.204	.320	.076	3.611	.300
	Lower-bound	30.904	3.000	10.301	1.204	.320	.076	3.611	.300
Error(fatigue)	Sphericity Assumed	376.591	44	8.559					
	Greenhouse-Geisser	376.591	44.000	8.559					
	Huynh-Feldt	376.591	44.000	8.559					
	Lower-bound	376.591	44.000	8.559					

a. Computed using alpha = .05

Tests of Within-Subjects Contrasts

Measure: MEASURE_1

Source	fatigue	Type III Sum of Squares	df	Mean Square	F	Sig.	Partial Eta Squared	Noncent. Parameter	Observed Power[a]
fatigue	Linear	1.956	1	1.956	.229	.635	.005	.229	.075
fatigue * groups	Linear	30.904	3	10.301	1.204	.320	.076	3.611	.300
Error(fatigue)	Linear	376.591	44	8.559					

a. Computed using alpha = .05

Levene's Test of Equality of Error Variances[a]

		Levene Statistic	df1	df2	Sig.
NtaPOpre	Based on Mean	5.619	3	44	.002
	Based on Median	4.431	3	44	.008
	Based on Median and with adjusted df	4.431	3	18.233	.017
	Based on trimmed mean	5.233	3	44	.004
NtaPOpos	Based on Mean	3.348	3	44	.027
	Based on Median	1.806	3	44	.160
	Based on Median and with adjusted df	1.806	3	21.467	.176
	Based on trimmed mean	3.056	3	44	.038

Tests the null hypothesis that the error variance of the dependent variable is equal across groups.

a. Design: Intercept + groups
Within Subjects Design: fatigue

Tests of Between-Subjects Effects

Measure: MEASURE_1
Transformed Variable: Average

Source	Type III Sum of Squares	df	Mean Square	F	Sig.	Partial Eta Squared	Noncent. Parameter	Observed Power[a]
Intercept	5862.001	1	5862.001	290.900	.000	.869	290.900	1.000
groups	319.395	3	106.465	5.283	.003	.265	15.850	.907
Error	886.654	44	20.151					

a. Computed using alpha = .05

Parameter Estimates

Dependent Variable	Parameter	B	Std. Error	t	Sig.	95% Confidence Interval Lower Bound	95% Confidence Interval Upper Bound	Partial Eta Squared	Noncent. Parameter	Observed Power[b]
NtaPOpre	Intercept	6.215	1.258	4.942	.000	3.680	8.750	.357	4.942	.998
	[groups=1.00]	6.481	1.875	3.457	.001	2.703	10.260	.214	3.457	.922
	[groups=2.00]	.500	1.634	.306	.761	-2.792	3.793	.002	.306	.060
	[groups=3.00]	.672	1.741	.386	.701	-2.837	4.181	.003	.386	.067
	[groups=4.00]	0[a]	.	.	.	.	.	.	.	.
NtaPOpos	Intercept	6.988	1.014	6.891	.000	4.944	9.031	.519	6.891	1.000
	[groups=1.00]	3.258	1.512	2.155	.037	.211	6.305	.095	2.155	.559
	[groups=2.00]	-.177	1.317	-.134	.894	-2.832	2.478	.000	.134	.052
	[groups=3.00]	.316	1.404	.225	.823	-2.513	3.145	.001	.225	.056
	[groups=4.00]	0[a]	.	.	.	.	.	.	.	.

a. This parameter is set to zero because it is redundant.

b. Computed using alpha = .05

Estimated Marginal Means

1. Grand Mean

Measure: MEASURE_1

Mean	Std. Error	95% Confidence Interval Lower Bound	95% Confidence Interval Upper Bound
7.983	.468	7.039	8.926

2. groups

Estimates

Measure: MEASURE_1

groups	Mean	Std. Error	95% Confidence Interval

			Lower Bound	Upper Bound
1.00	11.471	1.058	9.339	13.603
2.00	6.763	.794	5.164	8.362
3.00	7.095	.916	5.249	8.942
4.00	6.601	.957	4.672	8.530

Pairwise Comparisons

Measure: MEASURE_1

(I) groups	(J) groups	Mean Difference (I-J)	Std. Error	Sig.[b]	95% Confidence Interval for Difference[b] Lower Bound	Upper Bound
1.00	2.00	4.708*	1.323	.005	1.054	8.362
	3.00	4.376*	1.400	.019	.508	8.243
	4.00	4.870*	1.427	.008	.928	8.811
2.00	1.00	-4.708*	1.323	.005	-8.362	-1.054
	3.00	-.333	1.212	1.000	-3.682	3.016
	4.00	.162	1.243	1.000	-3.273	3.596
3.00	1.00	-4.376*	1.400	.019	-8.243	-.508
	2.00	.333	1.212	1.000	-3.016	3.682
	4.00	.494	1.325	1.000	-3.167	4.155
4.00	1.00	-4.870*	1.427	.008	-8.811	-.928
	2.00	-.162	1.243	1.000	-3.596	3.273
	3.00	-.494	1.325	1.000	-4.155	3.167

Based on estimated marginal means

*. The mean difference is significant at the .05 level.

b. Adjustment for multiple comparisons: Bonferroni.

Univariate Tests

Measure: MEASURE_1

	Sum of Squares	df	Mean Square	F	Sig.	Partial Eta Squared	Noncent. Parameter	Observed Power[a]
Contrast	159.698	3	53.233	5.283	.003	.265	15.850	.907
Error	443.327	44	10.076					

The F tests the effect of groups. This test is based on the linearly independent pairwise comparisons among the estimated marginal means.

a. Computed using alpha = .05

3. fatigue

Estimates

Measure: MEASURE_1

fatigue	Mean	Std. Error	95% Confidence Interval Lower Bound	95% Confidence Interval Upper Bound
1	8.128	.615	6.889	9.368
2	7.837	.496	6.837	8.836

Pairwise Comparisons

Measure: MEASURE_1

(I) fatigue	(J) fatigue	Mean Difference (I-J)	Std. Error	Sig.[a]	95% Confidence Interval for Difference[a] Lower Bound	95% Confidence Interval for Difference[a] Upper Bound
1	2	.292	.610	.635	-.938	1.521
2	1	-.292	.610	.635	-1.521	.938

Based on estimated marginal means

a. Adjustment for multiple comparisons: Bonferroni.

Multivariate Tests

	Value	F	Hypothesis df	Error df	Sig.	Partial Eta Squared	Noncent. Parameter	Observed Power[b]
Pillai's trace	.005	.229[a]	1.000	44.000	.635	.005	.229	.075
Wilks' lambda	.995	.229[a]	1.000	44.000	.635	.005	.229	.075
Hotelling's trace	.005	.229[a]	1.000	44.000	.635	.005	.229	.075
Roy's largest root	.005	.229[a]	1.000	44.000	.635	.005	.229	.075

Each F tests the multivariate effect of fatigue. These tests are based on the linearly independent pairwise comparisons among the estimated marginal means.

a. Exact statistic

b. Computed using alpha = .05

4. groups * fatigue

Measure: MEASURE_1

groups	fatigue	Mean	Std. Error	95% Confidence Interval Lower Bound	95% Confidence Interval Upper Bound
1.00	1	12.696	1.390	9.894	15.498

	2	10.246	1.121	7.986	12.505
2.00	1	6.715	1.043	4.614	8.817
	2	6.810	.841	5.116	8.505
3.00	1	6.887	1.204	4.461	9.314
	2	7.304	.971	5.347	9.260
4.00	1	6.215	1.258	3.680	8.750
	2	6.988	1.014	4.944	9.031

Post Hoc Tests

groups

Multiple Comparisons

Measure: MEASURE_1
Tukey HSD

(I) groups	(J) groups	Mean Difference (I-J)	Std. Error	Sig.	95% Confidence Interval Lower Bound	Upper Bound
1.00	2.00	4.7082*	1.32259	.005	1.1769	8.2395
	3.00	4.3756*	1.39970	.016	.6384	8.1128
	4.00	4.8697*	1.42670	.007	1.0604	8.6790
2.00	1.00	-4.7082*	1.32259	.005	-8.2395	-1.1769
	3.00	-.3326	1.21217	.993	-3.5691	2.9039
	4.00	.1615	1.24326	.999	-3.1580	3.4810
3.00	1.00	-4.3756*	1.39970	.016	-8.1128	-.6384
	2.00	.3326	1.21217	.993	-2.9039	3.5691
	4.00	.4941	1.32499	.982	-3.0436	4.0319
4.00	1.00	-4.8697*	1.42670	.007	-8.6790	-1.0604
	2.00	-.1615	1.24326	.999	-3.4810	3.1580
	3.00	-.4941	1.32499	.982	-4.0319	3.0436

Based on observed means.
The error term is Mean Square(Error) = 10.076.

*. The mean difference is significant at the .05 level.

Homogeneous Subsets

MEASURE_1

Tukey HSD[a,b,c]

groups	N	Subset 1	Subset 2
4.00	15	6.6013	
2.00	16	6.7628	
3.00	12	7.0954	
1.00	10		11.4710
Sig.		.982	1.000

Means for groups in homogeneous subsets are displayed.
Based on observed means.
The error term is Mean Square(Error) = 10.076.
a. Uses Harmonic Mean Sample Size = 11.499.
b. The group sizes are unequal. The harmonic mean of the group sizes is used. Type I error levels are not guaranteed.
c. Alpha = .05.

2.2. Gastrocnemius (GC) - Late Stance

```
GLM NgasPOpre NgasPOpos BY groups
  /WSFACTOR=fatigue 2 Polynomial
  /METHOD=SSTYPE(3)
  /POSTHOC=groups(TUKEY)
  /EMMEANS=TABLES(OVERALL)
  /EMMEANS=TABLES(groups) COMPARE ADJ(BONFERRONI)
  /EMMEANS=TABLES(fatigue) COMPARE ADJ(BONFERRONI)
  /EMMEANS=TABLES(groups*fatigue)
  /PRINT=DESCRIPTIVE ETASQ OPOWER PARAMETER HOMOGENEITY
  /CRITERIA=ALPHA(.05)
  /WSDESIGN=fatigue
  /DESIGN=groups.
```

General Linear Model

Notes

Output Created		17-MAY-2026 21:29:38
Comments		
Input	Data	C:\Users\RaSa\Desktop\پروپوزال\New folder\data\emgmi1.sav
	Active Dataset	DataSet2
	Filter	<none>
	Weight	<none>
	Split File	<none>
	N of Rows in Working Data File	48
Missing Value Handling	Definition of Missing	User-defined missing values are treated as missing.
	Cases Used	Statistics are based on all cases with valid data for all variables in the model.

Syntax		GLM NgasPOpre NgasPOpos BY groups<br>/WSFACTOR=fatigue 2 Polynomial<br>/METHOD=SSTYPE(3)<br>/POSTHOC=groups(TUKEY)<br>/EMMEANS=TABLES(OVERALL)<br>/EMMEANS=TABLES(groups) COMPARE ADJ(BONFERRONI)<br>/EMMEANS=TABLES(fatigue) COMPARE ADJ(BONFERRONI)<br>/EMMEANS=TABLES(groups*fatigue)<br>/PRINT=DESCRIPTIVE ETASQ OPOWER PARAMETER HOMOGENEITY<br>/CRITERIA=ALPHA(.05)<br>/WSDESIGN=fatigue<br>/DESIGN=groups.
Resources	Processor Time	00:00:00.03
	Elapsed Time	00:00:00.03

Within-Subjects Factors

Measure: MEASURE_1

fatigue	Dependent Variable
1	NgasPOpre
2	NgasPOpos

Between-Subjects Factors

		N
groups	1.00	10
	2.00	16
	3.00	12
	4.00	15

Descriptive Statistics

	groups	Mean	Std. Deviation	N
NgasPOpre	1.00	35.4337	8.48542	10
	2.00	36.4156	13.85894	16
	3.00	35.1353	14.58729	12
	4.00	28.0211	8.44266	15
	Total	33.9877	12.23099	53
NgasPOpos	1.00	36.3209	12.01018	10
	2.00	35.0905	12.19032	16
	3.00	36.1083	11.37273	12
	4.00	29.0132	6.42481	15
	Total	34.1829	10.92656	53

Box's Test of Equality of Covariance Matrices[a]

Box's M	11.100
F	1.126
df1	9
df2	12234.839
Sig.	.340

Tests the null hypothesis that the observed covariance matrices of the dependent variables are equal across groups.
a. Design: Intercept + groups
Within Subjects Design: fatigue

Multivariate Tests[a]

Effect		Value	F	Hypothesis df	Error df	Sig.	Partial Eta Squared	Noncent. Parameter	Observed Power[c]
fatigue	Pillai's Trace	.001	.049[b]	1.000	44.000	.826	.001	.049	.055
	Wilks' Lambda	.999	.049[b]	1.000	44.000	.826	.001	.049	.055
	Hotelling's Trace	.001	.049[b]	1.000	44.000	.826	.001	.049	.055
	Roy's Largest Root	.001	.049[b]	1.000	44.000	.826	.001	.049	.055
fatigue * groups	Pillai's Trace	.009	.135[b]	3.000	44.000	.938	.009	.406	.073
	Wilks' Lambda	.991	.135[b]	3.000	44.000	.938	.009	.406	.073

	Hotelling's Trace	.009	.135[b]	3.000	44.000	.938	.009	.406	.073
	Roy's Largest Root	.009	.135[b]	3.000	44.000	.938	.009	.406	.073

a. Design: Intercept + groups
Within Subjects Design: fatigue
b. Exact statistic
c. Computed using alpha = .05

Mauchly's Test of Sphericity[a]

Measure: MEASURE_1

Within Subjects Effect	Mauchly's W	Approx. Chi-Square	df	Sig.	Epsilon[b] Greenhouse-Geisser	Huynh-Feldt	Lower-bound
fatigue	1.000	.000	0	.	1.000	1.000	1.000

Tests the null hypothesis that the error covariance matrix of the orthonormalized transformed dependent variables is proportional to an identity matrix.
a. Design: Intercept + groups
Within Subjects Design: fatigue
b. May be used to adjust the degrees of freedom for the averaged tests of significance. Corrected tests are displayed in the Tests of Within-Subjects Effects table.

Tests of Within-Subjects Effects

Measure: MEASURE_1

Source		Type III Sum of Squares	df	Mean Square	F	Sig.	Partial Eta Squared	Noncent. Parameter	Observed Power[a]
fatigue	Sphericity Assumed	3.352	1	3.352	.049	.826	.001	.049	.055
	Greenhouse-Geisser	3.352	1.000	3.352	.049	.826	.001	.049	.055
	Huynh-Feldt	3.352	1.000	3.352	.049	.826	.001	.049	.055
	Lower-bound	3.352	1.000	3.352	.049	.826	.001	.049	.055
fatigue * groups	Sphericity Assumed	27.767	3	9.256	.135	.938	.009	.406	.073
	Greenhouse-Geisser	27.767	3.000	9.256	.135	.938	.009	.406	.073
	Huynh-Feldt	27.767	3.000	9.256	.135	.938	.009	.406	.073
	Lower-bound	27.767	3.000	9.256	.135	.938	.009	.406	.073
Error(fatigue)	Sphericity Assumed	3009.300	44	68.393					
	Greenhouse-Geisser	3009.300	44.000	68.393					
	Huynh-Feldt	3009.300	44.000	68.393					
	Lower-bound	3009.300	44.000	68.393					

a. Computed using alpha = .05

Tests of Within-Subjects Contrasts

Measure: MEASURE_1

Source	fatigue	Type III Sum of Squares	df	Mean Square	F	Sig.	Partial Eta Squared	Noncent. Parameter	Observed Power[a]
fatigue	Linear	3.352	1	3.352	.049	.826	.001	.049	.055
fatigue * groups	Linear	27.767	3	9.256	.135	.938	.009	.406	.073
Error(fatigue)	Linear	3009.300	44	68.393					

a. Computed using alpha = .05

Levene's Test of Equality of Error Variances[a]

		Levene Statistic	df1	df2	Sig.
NgasPOpre	Based on Mean	1.580	3	44	.208
	Based on Median	1.660	3	44	.189
	Based on Median and with adjusted df	1.660	3	39.566	.191
	Based on trimmed mean	1.575	3	44	.209
NgasPOpos	Based on Mean	1.562	3	44	.212
	Based on Median	1.458	3	44	.239
	Based on Median and with adjusted df	1.458	3	34.702	.243
	Based on trimmed mean	1.556	3	44	.214

Tests the null hypothesis that the error variance of the dependent variable is equal across groups.

a. Design: Intercept + groups
Within Subjects Design: fatigue

Tests of Between-Subjects Effects

Measure: MEASURE_1
Transformed Variable: Average

Source	Type III Sum of Squares	df	Mean Square	F	Sig.	Partial Eta Squared	Noncent. Parameter	Observed Power[a]
Intercept	105983.094	1	105983.094	534.792	.000	.924	534.792	1.000
groups	885.556	3	295.185	1.490	.231	.092	4.469	.366
Error	8719.759	44	198.176					

a. Computed using alpha = .05

Parameter Estimates

Dependent Variable	Parameter	B	Std. Error	t	Sig.	95% Confidence Interval		Partial Eta Squared	Noncent. Parameter	Observed Power[b]
						Lower Bound	Upper Bound			
NgasPOpre	Intercept	28.021	3.668	7.640	.000	20.630	35.413	.570	7.640	1.000
	[groups=1.00]	7.413	5.467	1.356	.182	-3.606	18.431	.040	1.356	.264
	[groups=2.00]	8.394	4.764	1.762	.085	-1.208	17.996	.066	1.762	.407
	[groups=3.00]	7.114	5.078	1.401	.168	-3.119	17.347	.043	1.401	.278
	[groups=4.00]	0[a]	.	.	.	.	.	.	.	.
NgasPOpos	Intercept	29.013	3.284	8.836	.000	22.396	35.631	.640	8.836	1.000
	[groups=1.00]	7.308	4.895	1.493	.143	-2.557	17.173	.048	1.493	.309
	[groups=2.00]	6.077	4.266	1.425	.161	-2.519	14.674	.044	1.425	.286
	[groups=3.00]	7.095	4.546	1.561	.126	-2.067	16.257	.052	1.561	.333
	[groups=4.00]	0[a]	.	.	.	.	.	.	.	.

a. This parameter is set to zero because it is redundant.
b. Computed using alpha = .05

Estimated Marginal Means

1. Grand Mean

Measure: MEASURE_1

Mean	Std. Error	95% Confidence Interval	
		Lower Bound	Upper Bound
33.942	1.468	30.984	36.900

2. groups

Estimates

Measure: MEASURE_1

groups	Mean	Std. Error	95% Confidence Interval

			Lower Bound	Upper Bound
1.00	35.877	3.318	29.190	42.564
2.00	35.753	2.489	30.738	40.768
3.00	35.622	2.874	29.831	41.413
4.00	28.517	3.001	22.468	34.566

Pairwise Comparisons

Measure: MEASURE_1

(I) groups	(J) groups	Mean Difference (I-J)	Std. Error	Sig.[a]	95% Confidence Interval for Difference[a] Lower Bound	Upper Bound
1.00	2.00	.124	4.148	1.000	-11.335	11.583
	3.00	.256	4.389	1.000	-11.872	12.383
	4.00	7.360	4.474	.643	-5.001	19.721
2.00	1.00	-.124	4.148	1.000	-11.583	11.335
	3.00	.131	3.801	1.000	-10.371	10.634
	4.00	7.236	3.899	.421	-3.536	18.008
3.00	1.00	-.256	4.389	1.000	-12.383	11.872
	2.00	-.131	3.801	1.000	-10.634	10.371
	4.00	7.105	4.155	.566	-4.375	18.585
4.00	1.00	-7.360	4.474	.643	-19.721	5.001
	2.00	-7.236	3.899	.421	-18.008	3.536
	3.00	-7.105	4.155	.566	-18.585	4.375

Based on estimated marginal means

a. Adjustment for multiple comparisons: Bonferroni.

Univariate Tests

Measure: MEASURE_1

	Sum of Squares	df	Mean Square	F	Sig.	Partial Eta Squared	Noncent. Parameter	Observed Power[a]
Contrast	442.778	3	147.593	1.490	.231	.092	4.469	.366
Error	4359.879	44	99.088					

The F tests the effect of groups. This test is based on the linearly independent pairwise comparisons among the estimated marginal means.

a. Computed using alpha = .05

3. fatigue

Estimates

Measure: MEASURE_1

fatigue	Mean	Std. Error	95% Confidence Interval Lower Bound	95% Confidence Interval Upper Bound
1	33.751	1.794	30.137	37.366
2	34.133	1.606	30.897	37.369

Pairwise Comparisons

Measure: MEASURE_1

(I) fatigue	(J) fatigue	Mean Difference (I-J)	Std. Error	Sig.[a]	95% Confidence Interval for Difference[a] Lower Bound	95% Confidence Interval for Difference[a] Upper Bound
1	2	-.382	1.724	.826	-3.857	3.094
2	1	.382	1.724	.826	-3.094	3.857

Based on estimated marginal means

a. Adjustment for multiple comparisons: Bonferroni.

Multivariate Tests

	Value	F	Hypothesis df	Error df	Sig.	Partial Eta Squared	Noncent. Parameter	Observed Power[b]
Pillai's trace	.001	.049[a]	1.000	44.000	.826	.001	.049	.055
Wilks' lambda	.999	.049[a]	1.000	44.000	.826	.001	.049	.055
Hotelling's trace	.001	.049[a]	1.000	44.000	.826	.001	.049	.055
Roy's largest root	.001	.049[a]	1.000	44.000	.826	.001	.049	.055

Each F tests the multivariate effect of fatigue. These tests are based on the linearly independent pairwise comparisons among the estimated marginal means.

a. Exact statistic

b. Computed using alpha = .05

4. groups * fatigue

Measure: MEASURE_1

groups	fatigue	Mean	Std. Error	95% Confidence Interval Lower Bound	95% Confidence Interval Upper Bound
1.00	1	35.434	4.055	27.262	43.605
	2	36.321	3.630	29.005	43.637

2.00	1	36.416	3.041	30.287	42.544
	2	35.090	2.723	29.603	40.578
3.00	1	35.135	3.511	28.058	42.212
	2	36.108	3.144	29.772	42.444
4.00	1	28.021	3.668	20.630	35.413
	2	29.013	3.284	22.396	35.631

Post Hoc Tests

groups

Multiple Comparisons

Measure: MEASURE_1
Tukey HSD

(I) groups	(J) groups	Mean Difference (I-J)	Std. Error	Sig.	95% Confidence Interval Lower Bound	Upper Bound
1.00	2.00	.1243	4.14763	1.000	-10.9499	11.1984
	3.00	.2555	4.38944	1.000	-11.4643	11.9753
	4.00	7.3601	4.47413	.365	-4.5858	19.3061
2.00	1.00	-.1243	4.14763	1.000	-11.1984	10.9499
	3.00	.1313	3.80136	1.000	-10.0184	10.2809
	4.00	7.2359	3.89885	.262	-3.1741	17.6458
3.00	1.00	-.2555	4.38944	1.000	-11.9753	11.4643
	2.00	-.1313	3.80136	1.000	-10.2809	10.0184
	4.00	7.1046	4.15516	.331	-3.9897	18.1989
4.00	1.00	-7.3601	4.47413	.365	-19.3061	4.5858
	2.00	-7.2359	3.89885	.262	-17.6458	3.1741
	3.00	-7.1046	4.15516	.331	-18.1989	3.9897

Based on observed means.
The error term is Mean Square(Error) = 99.088.

Homogeneous Subsets

MEASURE_1

Tukey HSD[a,b,c]

groups	N	Subset 1
4.00	15	28.5172
3.00	12	35.6218
2.00	16	35.7530
1.00	10	35.8773
Sig.		.300

Means for groups in homogeneous subsets are displayed.
Based on observed means.
The error term is Mean Square(Error) = 99.088.
a. Uses Harmonic Mean Sample Size = 11.499.
b. The group sizes are unequal. The harmonic mean of the group sizes is used. Type I error levels are not guaranteed.
c. Alpha = .05.

2.3. Vastus Lateralis (VL) - Late Stance

```
GLM NvlPOpre NvlPOpos BY groups
  /WSFACTOR=fatigue 2 Polynomial
  /METHOD=SSTYPE(3)
  /POSTHOC=groups(TUKEY)
  /EMMEANS=TABLES(OVERALL)
  /EMMEANS=TABLES(groups) COMPARE ADJ(BONFERRONI)
  /EMMEANS=TABLES(fatigue) COMPARE ADJ(BONFERRONI)
  /EMMEANS=TABLES(groups*fatigue)
  /PRINT=DESCRIPTIVE ETASQ OPOWER PARAMETER HOMOGENEITY
  /CRITERIA=ALPHA(.05)
  /WSDESIGN=fatigue
  /DESIGN=groups.
```

General Linear Model

Notes

Output Created		17-MAY-2026 21:30:21
Comments		
Input	Data	C:\Users\RaSa\Desktop\پروپوزال\New folder\data\emgmi1.sav
	Active Dataset	DataSet2
	Filter	<none>
	Weight	<none>
	Split File	<none>
	N of Rows in Working Data File	48
Missing Value Handling	Definition of Missing	User-defined missing values are treated as missing.
	Cases Used	Statistics are based on all cases with valid data for all variables in the model.

Syntax		GLM NvlPOpre NvlPOpos BY groups<br>/WSFACTOR=fatigue 2 Polynomial<br>/METHOD=SSTYPE(3)<br>/POSTHOC=groups(TUKEY)<br>/EMMEANS=TABLES(OVERALL)<br>/EMMEANS=TABLES(groups) COMPARE ADJ(BONFERRONI)<br>/EMMEANS=TABLES(fatigue) COMPARE ADJ(BONFERRONI)<br>/EMMEANS=TABLES(groups*fatigue)<br>/PRINT=DESCRIPTIVE ETASQ OPOWER PARAMETER HOMOGENEITY<br>/CRITERIA=ALPHA(.05)<br>/WSDESIGN=fatigue<br>/DESIGN=groups.
Resources	Processor Time	00:00:00.03
	Elapsed Time	00:00:00.03

Within-Subjects Factors

Measure: MEASURE_1

fatigue	Dependent Variable
1	NvlPOpre
2	NvlPOpos

Between-Subjects Factors

		N
groups	1.00	10
	2.00	16
	3.00	12
	4.00	15

Descriptive Statistics

	groups	Mean	Std. Deviation	N
NvIPOpre	1.00	30.6595	18.57633	10
	2.00	24.8682	12.38781	16
	3.00	30.7375	12.19734	12
	4.00	17.7888	9.09996	15
	Total	25.7990	13.64603	53
NvIPOpos	1.00	34.1649	15.16848	10
	2.00	24.0532	11.93849	16
	3.00	22.4463	12.34061	12
	4.00	22.3777	13.32903	15
	Total	25.1635	13.33155	53

Box's Test of Equality of Covariance Matrices[a]

Box's M	8.329
F	.845
df1	9
df2	12234.839
Sig.	.575

Tests the null hypothesis that the observed covariance matrices of the dependent variables are equal across groups.
a. Design: Intercept + groups
Within Subjects Design: fatigue

Multivariate Tests[a]

Effect		Value	F	Hypothesis df	Error df	Sig.	Partial Eta Squared	Noncent. Parameter	Observed Power[c]
fatigue	Pillai's Trace	.000	.016[b]	1.000	44.000	.901	.000	.016	.052
	Wilks' Lambda	1.000	.016[b]	1.000	44.000	.901	.000	.016	.052
	Hotelling's Trace	.000	.016[b]	1.000	44.000	.901	.000	.016	.052
	Roy's Largest Root	.000	.016[b]	1.000	44.000	.901	.000	.016	.052
fatigue * groups	Pillai's Trace	.124	2.071[b]	3.000	44.000	.118	.124	6.213	.494
	Wilks' Lambda	.876	2.071[b]	3.000	44.000	.118	.124	6.213	.494

	Hotelling's Trace	.141	2.071[b]	3.000	44.000	.118	.124	6.213	.494
	Roy's Largest Root	.141	2.071[b]	3.000	44.000	.118	.124	6.213	.494

a. Design: Intercept + groups
Within Subjects Design: fatigue
b. Exact statistic
c. Computed using alpha = .05

Mauchly's Test of Sphericity[a]

Measure: MEASURE_1

Within Subjects Effect	Mauchly's W	Approx. Chi-Square	df	Sig.	Epsilon[b] Greenhouse-Geisser	Epsilon[b] Huynh-Feldt	Epsilon[b] Lower-bound
fatigue	1.000	.000	0	.	1.000	1.000	1.000

Tests the null hypothesis that the error covariance matrix of the orthonormalized transformed dependent variables is proportional to an identity matrix.
a. Design: Intercept + groups
Within Subjects Design: fatigue
b. May be used to adjust the degrees of freedom for the averaged tests of significance. Corrected tests are displayed in the Tests of Within-Subjects Effects table.

Tests of Within-Subjects Effects

Measure: MEASURE_1

Source		Type III Sum of Squares	df	Mean Square	F	Sig.	Partial Eta Squared	Noncent. Parameter	Observed Power[a]
fatigue	Sphericity Assumed	1.471	1	1.471	.016	.901	.000	.016	.052
	Greenhouse-Geisser	1.471	1.000	1.471	.016	.901	.000	.016	.052
	Huynh-Feldt	1.471	1.000	1.471	.016	.901	.000	.016	.052
	Lower-bound	1.471	1.000	1.471	.016	.901	.000	.016	.052
fatigue * groups	Sphericity Assumed	579.202	3	193.067	2.071	.118	.124	6.213	.494
	Greenhouse-Geisser	579.202	3.000	193.067	2.071	.118	.124	6.213	.494
	Huynh-Feldt	579.202	3.000	193.067	2.071	.118	.124	6.213	.494
	Lower-bound	579.202	3.000	193.067	2.071	.118	.124	6.213	.494
Error(fatigue)	Sphericity Assumed	4101.686	44	93.220					
	Greenhouse-Geisser	4101.686	44.000	93.220					
	Huynh-Feldt	4101.686	44.000	93.220					
	Lower-bound	4101.686	44.000	93.220					

a. Computed using alpha = .05

Tests of Within-Subjects Contrasts

Measure: MEASURE_1

Source	fatigue	Type III Sum of Squares	df	Mean Square	F	Sig.	Partial Eta Squared	Noncent. Parameter	Observed Power[a]
fatigue	Linear	1.471	1	1.471	.016	.901	.000	.016	.052
fatigue * groups	Linear	579.202	3	193.067	2.071	.118	.124	6.213	.494
Error(fatigue)	Linear	4101.686	44	93.220					

a. Computed using alpha = .05

Levene's Test of Equality of Error Variances[a]

		Levene Statistic	df1	df2	Sig.
NvlPOpre	Based on Mean	1.337	3	44	.274
	Based on Median	.696	3	44	.559
	Based on Median and with adjusted df	.696	3	29.964	.562
	Based on trimmed mean	1.284	3	44	.292
NvlPOpos	Based on Mean	.443	3	44	.723
	Based on Median	.305	3	44	.821
	Based on Median and with adjusted df	.305	3	40.193	.821
	Based on trimmed mean	.453	3	44	.717

Tests the null hypothesis that the error variance of the dependent variable is equal across groups.

a. Design: Intercept + groups
Within Subjects Design: fatigue

Tests of Between-Subjects Effects

Measure: MEASURE_1
Transformed Variable: Average

Source	Type III Sum of Squares	df	Mean Square	F	Sig.	Partial Eta Squared	Noncent. Parameter	Observed Power[a]
Intercept	61647.794	1	61647.794	249.866	.000	.850	249.866	1.000
groups	1568.657	3	522.886	2.119	.111	.126	6.358	.505
Error	10855.842	44	246.724					

a. Computed using alpha = .05

Parameter Estimates

Dependent Variable	Parameter	B	Std. Error	t	Sig.	95% Confidence Interval		Partial Eta Squared	Noncent. Parameter	Observed Power[b]
						Lower Bound	Upper Bound			
NvlPOpre	Intercept	17.789	3.944	4.511	.000	9.841	25.737	.316	4.511	.993
	[groups=1.00]	12.871	5.879	2.189	.034	1.023	24.719	.098	2.189	.572
	[groups=2.00]	7.079	5.123	1.382	.174	-3.245	17.404	.042	1.382	.272
	[groups=3.00]	12.949	5.460	2.372	.022	1.945	23.952	.113	2.372	.640
	[groups=4.00]	0[a]	.	.	.	.	.	.	.	.
NvlPOpos	Intercept	22.378	3.918	5.711	.000	14.481	30.274	.426	5.711	1.000
	[groups=1.00]	11.787	5.841	2.018	.050	.016	23.559	.085	2.018	.506
	[groups=2.00]	1.676	5.090	.329	.744	-8.582	11.933	.002	.329	.062
	[groups=3.00]	.069	5.424	.013	.990	-10.864	11.001	.000	.013	.050
	[groups=4.00]	0[a]	.	.	.	.	.	.	.	.

a. This parameter is set to zero because it is redundant.
b. Computed using alpha = .05

Estimated Marginal Means

1. Grand Mean

Measure: MEASURE_1

Mean	Std. Error	95% Confidence Interval	
		Lower Bound	Upper Bound
25.887	1.638	22.586	29.188

2. groups

Estimates

Measure: MEASURE_1

groups	Mean	Std. Error	95% Confidence Interval

			Lower Bound	Upper Bound
1.00	32.412	3.702	24.951	39.874
2.00	24.461	2.777	18.865	30.057
3.00	26.592	3.206	20.130	33.054
4.00	20.083	3.349	13.334	26.832

Pairwise Comparisons

Measure: MEASURE_1

(I) groups	(J) groups	Mean Difference (I-J)	Std. Error	Sig.[a]	95% Confidence Interval for Difference[a] Lower Bound	Upper Bound
1.00	2.00	7.951	4.628	.557	-4.834	20.737
	3.00	5.820	4.898	1.000	-7.711	19.352
	4.00	12.329	4.992	.105	-1.463	26.121
2.00	1.00	-7.951	4.628	.557	-20.737	4.834
	3.00	-2.131	4.241	1.000	-13.850	9.587
	4.00	4.377	4.350	1.000	-7.642	16.396
3.00	1.00	-5.820	4.898	1.000	-19.352	7.711
	2.00	2.131	4.241	1.000	-9.587	13.850
	4.00	6.509	4.636	1.000	-6.300	19.318
4.00	1.00	-12.329	4.992	.105	-26.121	1.463
	2.00	-4.377	4.350	1.000	-16.396	7.642
	3.00	-6.509	4.636	1.000	-19.318	6.300

Based on estimated marginal means
a. Adjustment for multiple comparisons: Bonferroni.

Univariate Tests

Measure: MEASURE_1

	Sum of Squares	df	Mean Square	F	Sig.	Partial Eta Squared	Noncent. Parameter	Observed Power[a]
Contrast	784.329	3	261.443	2.119	.111	.126	6.358	.505
Error	5427.921	44	123.362					

The F tests the effect of groups. This test is based on the linearly independent pairwise comparisons among the estimated marginal means.
a. Computed using alpha = .05

3. fatigue

Estimates

Measure: MEASURE_1

fatigue	Mean	Std. Error	95% Confidence Interval	
			Lower Bound	Upper Bound
1	26.013	1.929	22.127	29.900
2	25.761	1.916	21.899	29.622

Pairwise Comparisons

Measure: MEASURE_1

(I) fatigue	(J) fatigue	Mean Difference (I-J)	Std. Error	Sig.[a]	95% Confidence Interval for Difference[a]	
					Lower Bound	Upper Bound
1	2	.253	2.013	.901	-3.805	4.310
2	1	-.253	2.013	.901	-4.310	3.805

Based on estimated marginal means

a. Adjustment for multiple comparisons: Bonferroni.

Multivariate Tests

	Value	F	Hypothesis df	Error df	Sig.	Partial Eta Squared	Noncent. Parameter	Observed Power[b]
Pillai's trace	.000	.016[a]	1.000	44.000	.901	.000	.016	.052
Wilks' lambda	1.000	.016[a]	1.000	44.000	.901	.000	.016	.052
Hotelling's trace	.000	.016[a]	1.000	44.000	.901	.000	.016	.052
Roy's largest root	.000	.016[a]	1.000	44.000	.901	.000	.016	.052

Each F tests the multivariate effect of fatigue. These tests are based on the linearly independent pairwise comparisons among the estimated marginal means.

a. Exact statistic

b. Computed using alpha = .05

4. groups * fatigue

Measure: MEASURE_1

groups	fatigue	Mean	Std. Error	95% Confidence Interval	
				Lower Bound	Upper Bound
1.00	1	30.659	4.360	21.873	39.446
	2	34.165	4.332	25.435	42.895

2.00	1	24.868	3.270	18.278	31.458
	2	24.053	3.249	17.506	30.601
3.00	1	30.738	3.776	23.128	38.347
	2	22.446	3.751	14.886	30.007
4.00	1	17.789	3.944	9.841	25.737
	2	22.378	3.918	14.481	30.274

Post Hoc Tests

groups

Multiple Comparisons

Measure: MEASURE_1
Tukey HSD

(I) groups	(J) groups	Mean Difference (I-J)	Std. Error	Sig.	95% Confidence Interval Lower Bound	95% Confidence Interval Upper Bound
1.00	2.00	7.9515	4.62785	.327	-4.4049	20.3079
	3.00	5.8203	4.89765	.637	-7.2565	18.8971
	4.00	12.3289	4.99215	.079	-1.0001	25.6580
2.00	1.00	-7.9515	4.62785	.327	-20.3079	4.4049
	3.00	-2.1312	4.24149	.958	-13.4560	9.1936
	4.00	4.3774	4.35027	.747	-7.2378	15.9927
3.00	1.00	-5.8203	4.89765	.637	-18.8971	7.2565
	2.00	2.1312	4.24149	.958	-9.1936	13.4560
	4.00	6.5086	4.63626	.504	-5.8702	18.8875
4.00	1.00	-12.3289	4.99215	.079	-25.6580	1.0001
	2.00	-4.3774	4.35027	.747	-15.9927	7.2378
	3.00	-6.5086	4.63626	.504	-18.8875	5.8702

Based on observed means.
The error term is Mean Square(Error) = 123.362.

Homogeneous Subsets

MEASURE_1

Tukey HSD[a,b,c]

groups	N	Subset 1
4.00	15	20.0833
2.00	16	24.4607
3.00	12	26.5919
1.00	10	32.4122
Sig.		.051

Means for groups in homogeneous subsets are displayed.
Based on observed means.
The error term is Mean Square(Error) = 123.362.
a. Uses Harmonic Mean Sample Size = 11.499.
b. The group sizes are unequal. The harmonic mean of the group sizes is used. Type I error levels are not guaranteed.
c. Alpha = .05.

2.4. Rectus Femoris (RF) - Late Stance

```
GLM NrfPOpre NrfPOpos BY groups
  /WSFACTOR=fatigue 2 Polynomial
  /METHOD=SSTYPE(3)
  /POSTHOC=groups(TUKEY)
  /EMMEANS=TABLES(OVERALL)
  /EMMEANS=TABLES(groups) COMPARE ADJ(BONFERRONI)
  /EMMEANS=TABLES(fatigue) COMPARE ADJ(BONFERRONI)
  /EMMEANS=TABLES(groups*fatigue)
  /PRINT=DESCRIPTIVE ETASQ OPOWER PARAMETER HOMOGENEITY
  /CRITERIA=ALPHA(.05)
  /WSDESIGN=fatigue
  /DESIGN=groups.
```

General Linear Model

Notes

Output Created		17-MAY-2026 21:31:01
Comments		
Input	Data	C:\Users\RaSa\Desktop\پروپوزال\New folder\data\emgmi1.sav
	Active Dataset	DataSet2
	Filter	<none>
	Weight	<none>
	Split File	<none>
	N of Rows in Working Data File	48
Missing Value Handling	Definition of Missing	User-defined missing values are treated as missing.
	Cases Used	Statistics are based on all cases with valid data for all variables in the model.

Syntax		GLM NrfPOpre NrfPOpos BY groups<br>/WSFACTOR=fatigue 2 Polynomial<br>/METHOD=SSTYPE(3)<br>/POSTHOC=groups(TUKEY)<br>/EMMEANS=TABLES(OVERALL)<br>/EMMEANS=TABLES(groups) COMPARE ADJ(BONFERRONI)<br>/EMMEANS=TABLES(fatigue) COMPARE ADJ(BONFERRONI)<br>/EMMEANS=TABLES(groups*fatigue)<br>/PRINT=DESCRIPTIVE ETASQ OPOWER PARAMETER HOMOGENEITY<br>/CRITERIA=ALPHA(.05)<br>/WSDESIGN=fatigue<br>/DESIGN=groups.
Resources	Processor Time	00:00:00.03
	Elapsed Time	00:00:00.03

Within-Subjects Factors

Measure: MEASURE_1

fatigue	Dependent Variable
1	NrfPOpre
2	NrfPOpos

Between-Subjects Factors

		N
groups	1.00	10
	2.00	16
	3.00	12
	4.00	15

Descriptive Statistics

	groups	Mean	Std. Deviation	N
NrfPOpre	1.00	29.9294	17.40330	10
	2.00	24.1044	17.78046	16
	3.00	28.2457	14.23123	12
	4.00	12.5532	7.99366	15
	Total	23.5848	15.97732	53
NrfPOpos	1.00	38.8008	27.88830	10
	2.00	22.3793	13.48074	16
	3.00	21.3021	12.08426	12
	4.00	16.0221	10.82749	15
	Total	23.7322	17.57919	53

Box's Test of Equality of Covariance Matrices[a]

Box's M	21.316
F	2.161
df1	9
df2	12234.839
Sig.	.022

Tests the null hypothesis that the observed covariance matrices of the dependent variables are equal across groups.
a. Design: Intercept + groups
Within Subjects Design: fatigue

Multivariate Tests[a]

Effect		Value	F	Hypothesis df	Error df	Sig.	Partial Eta Squared	Noncent. Parameter	Observed Power[c]
fatigue	Pillai's Trace	.004	.161[b]	1.000	44.000	.690	.004	.161	.068
	Wilks' Lambda	.996	.161[b]	1.000	44.000	.690	.004	.161	.068
	Hotelling's Trace	.004	.161[b]	1.000	44.000	.690	.004	.161	.068
	Roy's Largest Root	.004	.161[b]	1.000	44.000	.690	.004	.161	.068
fatigue * groups	Pillai's Trace	.121	2.027[b]	3.000	44.000	.124	.121	6.081	.485
	Wilks' Lambda	.879	2.027[b]	3.000	44.000	.124	.121	6.081	.485

	Hotelling's Trace	.138	2.027[b]	3.000	44.000	.124	.121	6.081	.485
	Roy's Largest Root	.138	2.027[b]	3.000	44.000	.124	.121	6.081	.485

a. Design: Intercept + groups
Within Subjects Design: fatigue
b. Exact statistic
c. Computed using alpha = .05

Mauchly's Test of Sphericity[a]

Measure: MEASURE_1

Within Subjects Effect	Mauchly's W	Approx. Chi-Square	df	Sig.	Epsilon[b] Greenhouse-Geisser	Huynh-Feldt	Lower-bound
fatigue	1.000	.000	0	.	1.000	1.000	1.000

Tests the null hypothesis that the error covariance matrix of the orthonormalized transformed dependent variables is proportional to an identity matrix.
a. Design: Intercept + groups
Within Subjects Design: fatigue
b. May be used to adjust the degrees of freedom for the averaged tests of significance. Corrected tests are displayed in the Tests of Within-Subjects Effects table.

Tests of Within-Subjects Effects

Measure: MEASURE_1

Source		Type III Sum of Squares	df	Mean Square	F	Sig.	Partial Eta Squared	Noncent. Parameter	Observed Power[a]
fatigue	Sphericity Assumed	19.377	1	19.377	.161	.690	.004	.161	.068
	Greenhouse-Geisser	19.377	1.000	19.377	.161	.690	.004	.161	.068
	Huynh-Feldt	19.377	1.000	19.377	.161	.690	.004	.161	.068
	Lower-bound	19.377	1.000	19.377	.161	.690	.004	.161	.068
fatigue * groups	Sphericity Assumed	732.916	3	244.305	2.027	.124	.121	6.081	.485
	Greenhouse-Geisser	732.916	3.000	244.305	2.027	.124	.121	6.081	.485
	Huynh-Feldt	732.916	3.000	244.305	2.027	.124	.121	6.081	.485
	Lower-bound	732.916	3.000	244.305	2.027	.124	.121	6.081	.485
Error(fatigue)	Sphericity Assumed	5303.221	44	120.528					
	Greenhouse-Geisser	5303.221	44.000	120.528					
	Huynh-Feldt	5303.221	44.000	120.528					
	Lower-bound	5303.221	44.000	120.528					

a. Computed using alpha = .05

Tests of Within-Subjects Contrasts

Measure: MEASURE_1

Source	fatigue	Type III Sum of Squares	df	Mean Square	F	Sig.	Partial Eta Squared	Noncent. Parameter	Observed Power[a]
fatigue	Linear	19.377	1	19.377	.161	.690	.004	.161	.068
fatigue * groups	Linear	732.916	3	244.305	2.027	.124	.121	6.081	.485
Error(fatigue)	Linear	5303.221	44	120.528					

a. Computed using alpha = .05

Levene's Test of Equality of Error Variances[a]

		Levene Statistic	df1	df2	Sig.
NrfPOpre	Based on Mean	2.543	3	44	.068
	Based on Median	1.373	3	44	.263
	Based on Median and with adjusted df	1.373	3	33.216	.268
	Based on trimmed mean	2.300	3	44	.090
NrfPOpos	Based on Mean	7.325	3	44	.000
	Based on Median	2.339	3	44	.086
	Based on Median and with adjusted df	2.339	3	21.583	.102
	Based on trimmed mean	6.447	3	44	.001

Tests the null hypothesis that the error variance of the dependent variable is equal across groups.

a. Design: Intercept + groups
Within Subjects Design: fatigue

Tests of Between-Subjects Effects

Measure: MEASURE_1
Transformed Variable: Average

Source	Type III Sum of Squares	df	Mean Square	F	Sig.	Partial Eta Squared	Noncent. Parameter	Observed Power[a]
Intercept	53728.366	1	53728.366	143.664	.000	.766	143.664	1.000
groups	4030.655	3	1343.552	3.593	.021	.197	10.778	.755
Error	16455.428	44	373.987					

a. Computed using alpha = .05

Parameter Estimates

Dependent Variable	Parameter	B	Std. Error	t	Sig.	95% Confidence Interval Lower Bound	95% Confidence Interval Upper Bound	Partial Eta Squared	Noncent. Parameter	Observed Power[b]
NrfPOpre	Intercept	12.553	4.553	2.757	.008	3.378	21.729	.147	2.757	.769
	[groups=1.00]	17.376	6.787	2.560	.014	3.698	31.054	.130	2.560	.707
	[groups=2.00]	11.551	5.914	1.953	.057	-.368	23.470	.080	1.953	.480
	[groups=3.00]	15.693	6.303	2.490	.017	2.990	28.395	.123	2.490	.683
	[groups=4.00]	0[a]	.	.	.	.	.	.	.	.
NrfPOpos	Intercept	16.022	4.922	3.255	.002	6.102	25.942	.194	3.255	.889
	[groups=1.00]	22.779	7.338	3.104	.003	7.991	37.567	.180	3.104	.859
	[groups=2.00]	6.357	6.394	.994	.326	-6.530	19.244	.022	.994	.163
	[groups=3.00]	5.280	6.815	.775	.443	-8.454	19.014	.013	.775	.118
	[groups=4.00]	0[a]	.	.	.	.	.	.	.	.

a. This parameter is set to zero because it is redundant.
b. Computed using alpha = .05

Estimated Marginal Means

1. Grand Mean

Measure: MEASURE_1

Mean	Std. Error	95% Confidence Interval Lower Bound	95% Confidence Interval Upper Bound
24.167	2.016	20.104	28.231

2. groups

Estimates

Measure: MEASURE_1

groups	Mean	Std. Error	95% Confidence Interval

			Lower Bound	Upper Bound
1.00	34.365	4.558	25.179	43.552
2.00	23.242	3.419	16.352	30.132
3.00	24.774	3.948	16.818	32.730
4.00	14.288	4.123	5.978	22.597

Pairwise Comparisons

Measure: MEASURE_1

(I) groups	(J) groups	Mean Difference (I-J)	Std. Error	Sig.[b]	95% Confidence Interval for Difference[b] Lower Bound	Upper Bound
1.00	2.00	11.123	5.698	.344	-4.618	26.865
	3.00	9.591	6.030	.713	-7.068	26.251
	4.00	20.077*	6.146	.013	3.096	37.058
2.00	1.00	-11.123	5.698	.344	-26.865	4.618
	3.00	-1.532	5.222	1.000	-15.960	12.895
	4.00	8.954	5.356	.610	-5.843	23.752
3.00	1.00	-9.591	6.030	.713	-26.251	7.068
	2.00	1.532	5.222	1.000	-12.895	15.960
	4.00	10.486	5.708	.438	-5.284	26.257
4.00	1.00	-20.077*	6.146	.013	-37.058	-3.096
	2.00	-8.954	5.356	.610	-23.752	5.843
	3.00	-10.486	5.708	.438	-26.257	5.284

Based on estimated marginal means

*. The mean difference is significant at the .05 level.

b. Adjustment for multiple comparisons: Bonferroni.

Univariate Tests

Measure: MEASURE_1

	Sum of Squares	df	Mean Square	F	Sig.	Partial Eta Squared	Noncent. Parameter	Observed Power[a]
Contrast	2015.328	3	671.776	3.593	.021	.197	10.778	.755
Error	8227.714	44	186.994					

The F tests the effect of groups. This test is based on the linearly independent pairwise comparisons among the estimated marginal means.

a. Computed using alpha = .05

3. fatigue

Estimates

Measure: MEASURE_1

fatigue	Mean	Std. Error	95% Confidence Interval Lower Bound	Upper Bound
1	23.708	2.226	19.221	28.195
2	24.626	2.407	19.775	29.477

Pairwise Comparisons

Measure: MEASURE_1

(I) fatigue	(J) fatigue	Mean Difference (I-J)	Std. Error	Sig.[a]	95% Confidence Interval for Difference[a] Lower Bound	Upper Bound
1	2	-.918	2.289	.690	-5.532	3.696
2	1	.918	2.289	.690	-3.696	5.532

Based on estimated marginal means

a. Adjustment for multiple comparisons: Bonferroni.

Multivariate Tests

	Value	F	Hypothesis df	Error df	Sig.	Partial Eta Squared	Noncent. Parameter	Observed Power[b]
Pillai's trace	.004	.161[a]	1.000	44.000	.690	.004	.161	.068
Wilks' lambda	.996	.161[a]	1.000	44.000	.690	.004	.161	.068
Hotelling's trace	.004	.161[a]	1.000	44.000	.690	.004	.161	.068
Roy's largest root	.004	.161[a]	1.000	44.000	.690	.004	.161	.068

Each F tests the multivariate effect of fatigue. These tests are based on the linearly independent pairwise comparisons among the estimated marginal means.

a. Exact statistic

b. Computed using alpha = .05

4. groups * fatigue

Measure: MEASURE_1

groups	fatigue	Mean	Std. Error	95% Confidence Interval Lower Bound	Upper Bound
1.00	1	29.929	5.033	19.786	40.073

	2	38.801	5.442	27.834	49.768
2.00	1	24.104	3.775	16.497	31.712
	2	22.379	4.081	14.154	30.605
3.00	1	28.246	4.359	19.461	37.031
	2	21.302	4.713	11.804	30.800
4.00	1	12.553	4.553	3.378	21.729
	2	16.022	4.922	6.102	25.942

Post Hoc Tests

groups

Multiple Comparisons

Measure: MEASURE_1
Tukey HSD

(I) groups	(J) groups	Mean Difference (I-J)	Std. Error	Sig.	95% Confidence Interval Lower Bound	Upper Bound
1.00	2.00	11.1233	5.69773	.222	-4.0897	26.3363
	3.00	9.5912	6.02991	.394	-6.5087	25.6911
	4.00	20.0774*	6.14626	.011	3.6669	36.4880
2.00	1.00	-11.1233	5.69773	.222	-26.3363	4.0897
	3.00	-1.5321	5.22206	.991	-15.4750	12.4108
	4.00	8.9542	5.35598	.351	-5.3463	23.2546
3.00	1.00	-9.5912	6.02991	.394	-25.6911	6.5087
	2.00	1.5321	5.22206	.991	-12.4108	15.4750
	4.00	10.4862	5.70808	.270	-4.7544	25.7269
4.00	1.00	-20.0774*	6.14626	.011	-36.4880	-3.6669
	2.00	-8.9542	5.35598	.351	-23.2546	5.3463
	3.00	-10.4862	5.70808	.270	-25.7269	4.7544

Based on observed means.
The error term is Mean Square(Error) = 186.994.

*. The mean difference is significant at the .05 level.

Homogeneous Subsets

MEASURE_1

Tukey HSD[a,b,c]

groups	N	Subset	
		1	2
4.00	15	14.2877	
2.00	16	23.2418	23.2418
3.00	12	24.7739	24.7739
1.00	10		34.3651
Sig.		.269	.222

Means for groups in homogeneous subsets are displayed.
Based on observed means.
The error term is Mean Square(Error) = 186.994.
a. Uses Harmonic Mean Sample Size = 11.499.
b. The group sizes are unequal. The harmonic mean of the group sizes is used. Type I error levels are not guaranteed.
c. Alpha = .05.

2.5. Vastus Medialis (VM) - Late Stance

```
GLM NvmPOpre NvmPOpos BY groups
  /WSFACTOR=fatigue 2 Polynomial
  /METHOD=SSTYPE(3)
  /POSTHOC=groups(TUKEY)
  /EMMEANS=TABLES(OVERALL)
  /EMMEANS=TABLES(groups) COMPARE ADJ(BONFERRONI)
  /EMMEANS=TABLES(fatigue) COMPARE ADJ(BONFERRONI)
  /EMMEANS=TABLES(groups*fatigue)
  /PRINT=DESCRIPTIVE ETASQ OPOWER PARAMETER HOMOGENEITY
  /CRITERIA=ALPHA(.05)
  /WSDESIGN=fatigue
  /DESIGN=groups.
```

General Linear Model

Notes

Output Created		17-MAY-2026 21:32:07
Comments		
Input	Data	C:\Users\RaSa\Desktop\پروپوزال\New folder\data\emgmi1.sav
	Active Dataset	DataSet2
	Filter	<none>
	Weight	<none>
	Split File	<none>
	N of Rows in Working Data File	48
Missing Value Handling	Definition of Missing	User-defined missing values are treated as missing.
	Cases Used	Statistics are based on all cases with valid data for all variables in the model.

Syntax		GLM NvmPOpre NvmPOpos BY groups<br>/WSFACTOR=fatigue 2 Polynomial<br>/METHOD=SSTYPE(3)<br>/POSTHOC=groups(TUKEY)<br>/EMMEANS=TABLES(OVERALL)<br>/EMMEANS=TABLES(groups) COMPARE ADJ(BONFERRONI)<br>/EMMEANS=TABLES(fatigue) COMPARE ADJ(BONFERRONI)<br>/EMMEANS=TABLES(groups*fatigue)<br>/PRINT=DESCRIPTIVE ETASQ OPOWER PARAMETER HOMOGENEITY<br>/CRITERIA=ALPHA(.05)<br>/WSDESIGN=fatigue<br>/DESIGN=groups.
Resources	Processor Time	00:00:00.02
	Elapsed Time	00:00:00.02

Within-Subjects Factors

Measure: MEASURE_1

fatigue	Dependent Variable
1	NvmPOpre
2	NvmPOpos

Between-Subjects Factors

		N
groups	1.00	10
	2.00	16
	3.00	12
	4.00	15

Descriptive Statistics

	groups	Mean	Std. Deviation	N
NvmPOpre	1.00	19.6476	9.56528	10
	2.00	19.3760	18.63052	16
	3.00	25.6834	12.64309	12
	4.00	11.0192	3.40093	15
	Total	19.0887	13.87995	53
NvmPOpos	1.00	20.0287	9.95842	10
	2.00	19.2547	14.07828	16
	3.00	14.9285	6.08137	12
	4.00	14.8908	9.10496	15
	Total	17.3182	10.58060	53

Box's Test of Equality of Covariance Matrices[a]

Box's M	35.747
F	3.625
df1	9
df2	12234.839
Sig.	.000

Tests the null hypothesis that the observed covariance matrices of the dependent variables are equal across groups.
a. Design: Intercept + groups
Within Subjects Design: fatigue

Multivariate Tests[a]

Effect		Value	F	Hypothesis df	Error df	Sig.	Partial Eta Squared	Noncent. Parameter	Observed Power[c]
fatigue	Pillai's Trace	.034	1.529[b]	1.000	44.000	.223	.034	1.529	.227
	Wilks' Lambda	.966	1.529[b]	1.000	44.000	.223	.034	1.529	.227
	Hotelling's Trace	.035	1.529[b]	1.000	44.000	.223	.034	1.529	.227
	Roy's Largest Root	.035	1.529[b]	1.000	44.000	.223	.034	1.529	.227
fatigue * groups	Pillai's Trace	.279	5.672[b]	3.000	44.000	.002	.279	17.017	.927
	Wilks' Lambda	.721	5.672[b]	3.000	44.000	.002	.279	17.017	.927

	Hotelling's Trace	.387	5.672[b]	3.000	44.000	.002	.279	17.017	.927
	Roy's Largest Root	.387	5.672[b]	3.000	44.000	.002	.279	17.017	.927

a. Design: Intercept + groups
 Within Subjects Design: fatigue
b. Exact statistic
c. Computed using alpha = .05

Mauchly's Test of Sphericity[a]

Measure: MEASURE_1

Within Subjects Effect	Mauchly's W	Approx. Chi-Square	df	Sig.	Epsilon[b] Greenhouse-Geisser	Huynh-Feldt	Lower-bound
fatigue	1.000	.000	0	.	1.000	1.000	1.000

Tests the null hypothesis that the error covariance matrix of the orthonormalized transformed dependent variables is proportional to an identity matrix.
a. Design: Intercept + groups
 Within Subjects Design: fatigue
b. May be used to adjust the degrees of freedom for the averaged tests of significance. Corrected tests are displayed in the Tests of Within-Subjects Effects table.

Tests of Within-Subjects Effects

Measure: MEASURE_1

Source		Type III Sum of Squares	df	Mean Square	F	Sig.	Partial Eta Squared	Noncent. Parameter	Observed Power[a]
fatigue	Sphericity Assumed	63.061	1	63.061	1.529	.223	.034	1.529	.227
	Greenhouse-Geisser	63.061	1.000	63.061	1.529	.223	.034	1.529	.227
	Huynh-Feldt	63.061	1.000	63.061	1.529	.223	.034	1.529	.227
	Lower-bound	63.061	1.000	63.061	1.529	.223	.034	1.529	.227
fatigue * groups	Sphericity Assumed	701.990	3	233.997	5.672	.002	.279	17.017	.927
	Greenhouse-Geisser	701.990	3.000	233.997	5.672	.002	.279	17.017	.927
	Huynh-Feldt	701.990	3.000	233.997	5.672	.002	.279	17.017	.927
	Lower-bound	701.990	3.000	233.997	5.672	.002	.279	17.017	.927
Error(fatigue)	Sphericity Assumed	1815.086	44	41.252					
	Greenhouse-Geisser	1815.086	44.000	41.252					
	Huynh-Feldt	1815.086	44.000	41.252					
	Lower-bound	1815.086	44.000	41.252					

a. Computed using alpha = .05

Tests of Within-Subjects Contrasts

Measure: MEASURE_1

Source	fatigue	Type III Sum of Squares	df	Mean Square	F	Sig.	Partial Eta Squared	Noncent. Parameter	Observed Power[a]
fatigue	Linear	63.061	1	63.061	1.529	.223	.034	1.529	.227
fatigue * groups	Linear	701.990	3	233.997	5.672	.002	.279	17.017	.927
Error(fatigue)	Linear	1815.086	44	41.252					

a. Computed using alpha = .05

Levene's Test of Equality of Error Variances[a]

		Levene Statistic	df1	df2	Sig.
NvmPOpre	Based on Mean	2.703	3	44	.057
	Based on Median	1.559	3	44	.213
	Based on Median and with adjusted df	1.559	3	19.192	.232
	Based on trimmed mean	2.092	3	44	.115
NvmPOpos	Based on Mean	.983	3	44	.410
	Based on Median	.757	3	44	.524
	Based on Median and with adjusted df	.757	3	32.337	.526
	Based on trimmed mean	.876	3	44	.461

Tests the null hypothesis that the error variance of the dependent variable is equal across groups.

a. Design: Intercept + groups

Within Subjects Design: fatigue

Tests of Between-Subjects Effects

Measure: MEASURE_1

Transformed Variable: Average

Source	Type III Sum of Squares	df	Mean Square	F	Sig.	Partial Eta Squared	Noncent. Parameter	Observed Power[a]
Intercept	30149.699	1	30149.699	120.605	.000	.733	120.605	1.000
groups	799.773	3	266.591	1.066	.373	.068	3.199	.269
Error	10999.452	44	249.988					

a. Computed using alpha = .05

Parameter Estimates

Dependent Variable	Parameter	B	Std. Error	t	Sig.	95% Confidence Interval		Partial Eta Squared	Noncent. Parameter	Observed Power[b]
						Lower Bound	Upper Bound			
NvmPOpre	Intercept	11.019	4.018	2.743	.009	2.922	19.116	.146	2.743	.765
	[groups=1.00]	8.628	5.989	1.441	.157	-3.442	20.699	.045	1.441	.291
	[groups=2.00]	8.357	5.219	1.601	.116	-2.161	18.875	.055	1.601	.347
	[groups=3.00]	14.664	5.562	2.636	.012	3.454	25.874	.136	2.636	.732
	[groups=4.00]	0[a]	.	.	.	.	.	.	.	.
NvmPOpos	Intercept	14.891	3.215	4.632	.000	8.412	21.370	.328	4.632	.995
	[groups=1.00]	5.138	4.792	1.072	.290	-4.520	14.796	.025	1.072	.182
	[groups=2.00]	4.364	4.176	1.045	.302	-4.053	12.780	.024	1.045	.176
	[groups=3.00]	.038	4.451	.008	.993	-8.932	9.007	.000	.008	.050
	[groups=4.00]	0[a]	.	.	.	.	.	.	.	.

a. This parameter is set to zero because it is redundant.
b. Computed using alpha = .05

Estimated Marginal Means

1. Grand Mean

Measure: MEASURE_1

Mean	Std. Error	95% Confidence Interval	
		Lower Bound	Upper Bound
18.104	1.648	14.781	21.426

2. groups

Estimates

Measure: MEASURE_1

groups	Mean	Std. Error	95% Confidence Interval

			Lower Bound	Upper Bound
1.00	19.838	3.727	12.328	27.349
2.00	19.315	2.795	13.682	24.948
3.00	20.306	3.227	13.801	26.810
4.00	12.955	3.371	6.161	19.749

Pairwise Comparisons

Measure: MEASURE_1

(I) groups	(J) groups	Mean Difference (I-J)	Std. Error	Sig.[a]	95% Confidence Interval for Difference[a] Lower Bound	Upper Bound
1.00	2.00	.523	4.658	1.000	-12.347	13.393
	3.00	-.468	4.930	1.000	-14.088	13.153
	4.00	6.883	5.025	1.000	-7.000	20.767
2.00	1.00	-.523	4.658	1.000	-13.393	12.347
	3.00	-.991	4.269	1.000	-12.786	10.805
	4.00	6.360	4.379	.921	-5.738	18.459
3.00	1.00	.468	4.930	1.000	-13.153	14.088
	2.00	.991	4.269	1.000	-10.805	12.786
	4.00	7.351	4.667	.734	-5.543	20.244
4.00	1.00	-6.883	5.025	1.000	-20.767	7.000
	2.00	-6.360	4.379	.921	-18.459	5.738
	3.00	-7.351	4.667	.734	-20.244	5.543

Based on estimated marginal means

a. Adjustment for multiple comparisons: Bonferroni.

Univariate Tests

Measure: MEASURE_1

	Sum of Squares	df	Mean Square	F	Sig.	Partial Eta Squared	Noncent. Parameter	Observed Power[a]
Contrast	399.887	3	133.296	1.066	.373	.068	3.199	.269
Error	5499.726	44	124.994					

The F tests the effect of groups. This test is based on the linearly independent pairwise comparisons among the estimated marginal means.

a. Computed using alpha = .05

3. fatigue

Estimates

Measure: MEASURE_1

fatigue	Mean	Std. Error	95% Confidence Interval Lower Bound	95% Confidence Interval Upper Bound
1	18.932	1.965	14.972	22.891
2	17.276	1.572	14.107	20.444

Pairwise Comparisons

Measure: MEASURE_1

(I) fatigue	(J) fatigue	Mean Difference (I-J)	Std. Error	Sig.[a]	95% Confidence Interval for Difference[a] Lower Bound	95% Confidence Interval for Difference[a] Upper Bound
1	2	1.656	1.339	.223	-1.043	4.355
2	1	-1.656	1.339	.223	-4.355	1.043

Based on estimated marginal means

a. Adjustment for multiple comparisons: Bonferroni.

Multivariate Tests

	Value	F	Hypothesis df	Error df	Sig.	Partial Eta Squared	Noncent. Parameter	Observed Power[b]
Pillai's trace	.034	1.529[a]	1.000	44.000	.223	.034	1.529	.227
Wilks' lambda	.966	1.529[a]	1.000	44.000	.223	.034	1.529	.227
Hotelling's trace	.035	1.529[a]	1.000	44.000	.223	.034	1.529	.227
Roy's largest root	.035	1.529[a]	1.000	44.000	.223	.034	1.529	.227

Each F tests the multivariate effect of fatigue. These tests are based on the linearly independent pairwise comparisons among the estimated marginal means.

a. Exact statistic

b. Computed using alpha = .05

4. groups * fatigue

Measure: MEASURE_1

groups	fatigue	Mean	Std. Error	95% Confidence Interval Lower Bound	95% Confidence Interval Upper Bound
1.00	1	19.648	4.442	10.696	28.599
	2	20.029	3.554	12.866	27.192

2.00	1	19.376	3.331	12.662	26.090
	2	19.255	2.666	13.883	24.627
3.00	1	25.683	3.847	17.931	33.436
	2	14.928	3.078	8.725	21.132
4.00	1	11.019	4.018	2.922	19.116
	2	14.891	3.215	8.412	21.370

Post Hoc Tests

groups

Multiple Comparisons

Measure: MEASURE_1
Tukey HSD

(I) groups	(J) groups	Mean Difference (I-J)	Std. Error	Sig.	95% Confidence Interval Lower Bound	95% Confidence Interval Upper Bound
1.00	2.00	.5228	4.65836	.999	-11.9150	12.9607
	3.00	-.4677	4.92994	1.000	-13.6307	12.6952
	4.00	6.8832	5.02506	.525	-6.5338	20.3001
2.00	1.00	-.5228	4.65836	.999	-12.9607	11.9150
	3.00	-.9906	4.26946	.996	-12.3900	10.4089
	4.00	6.3604	4.37895	.474	-5.3315	18.0522
3.00	1.00	.4677	4.92994	1.000	-12.6952	13.6307
	2.00	.9906	4.26946	.996	-10.4089	12.3900
	4.00	7.3509	4.66682	.403	-5.1095	19.8114
4.00	1.00	-6.8832	5.02506	.525	-20.3001	6.5338
	2.00	-6.3604	4.37895	.474	-18.0522	5.3315
	3.00	-7.3509	4.66682	.403	-19.8114	5.1095

Based on observed means.
The error term is Mean Square(Error) = 124.994.

Homogeneous Subsets

MEASURE_1

Tukey HSD[a,b,c]

groups	N	Subset<br>1
4.00	15	12.9550
2.00	16	19.3153
1.00	10	19.8382
3.00	12	20.3059
Sig.		.402

Means for groups in homogeneous subsets are displayed.
Based on observed means.
The error term is Mean Square(Error) = 124.994.
a. Uses Harmonic Mean Sample Size = 11.499.
b. The group sizes are unequal. The harmonic mean of the group sizes is used. Type I error levels are not guaranteed.
c. Alpha = .05.

2.6. Biceps Femoris (BF) - Late Stance

```
GLM NbfPOpre NbfPOpos BY groups
  /WSFACTOR=fatigue 2 Polynomial
  /METHOD=SSTYPE(3)
  /POSTHOC=groups(TUKEY)
  /EMMEANS=TABLES(OVERALL)
  /EMMEANS=TABLES(groups) COMPARE ADJ(BONFERRONI)
  /EMMEANS=TABLES(fatigue) COMPARE ADJ(BONFERRONI)
  /EMMEANS=TABLES(groups*fatigue)
  /PRINT=DESCRIPTIVE ETASQ OPOWER PARAMETER HOMOGENEITY
  /CRITERIA=ALPHA(.05)
  /WSDESIGN=fatigue
  /DESIGN=groups.
```

General Linear Model

Notes

Output Created		17-MAY-2026 21:32:30
Comments		
Input	Data	C:\Users\RaSa\Desktop\پروپوزال\New folder\data\emgmi1.sav
	Active Dataset	DataSet2
	Filter	<none>
	Weight	<none>
	Split File	<none>
	N of Rows in Working Data File	48
Missing Value Handling	Definition of Missing	User-defined missing values are treated as missing.
	Cases Used	Statistics are based on all cases with valid data for all variables in the model.

Syntax		GLM NbfPOpre NbfPOpos BY groups<br>/WSFACTOR=fatigue 2 Polynomial<br>/METHOD=SSTYPE(3)<br>/POSTHOC=groups(TUKEY)<br>/EMMEANS=TABLES(OVERALL)<br>/EMMEANS=TABLES(groups) COMPARE ADJ(BONFERRONI)<br>/EMMEANS=TABLES(fatigue) COMPARE ADJ(BONFERRONI)<br>/EMMEANS=TABLES(groups*fatigue)<br>/PRINT=DESCRIPTIVE ETASQ OPOWER PARAMETER HOMOGENEITY<br>/CRITERIA=ALPHA(.05)<br>/WSDESIGN=fatigue<br>/DESIGN=groups.
Resources	Processor Time	00:00:00.02
	Elapsed Time	00:00:00.01

Within-Subjects Factors

Measure: MEASURE_1

fatigue	Dependent Variable
1	NbfPOpre
2	NbfPOpos

Between-Subjects Factors

		N
groups	1.00	10
	2.00	16
	3.00	12
	4.00	15

Descriptive Statistics

	groups	Mean	Std. Deviation	N
NbfPOpre	1.00	16.0676	11.43198	10
	2.00	18.3793	8.42805	16
	3.00	20.1964	9.20602	12
	4.00	5.2542	3.98956	15
	Total	15.3923	10.06029	53
NbfPOpos	1.00	17.9999	16.48478	10
	2.00	26.0497	29.44733	16
	3.00	20.0665	10.89391	12
	4.00	5.0706	3.33929	15
	Total	18.2368	20.38058	53

Box's Test of Equality of Covariance Matrices[a]

Box's M	59.705
F	6.054
df1	9
df2	12234.839
Sig.	.000

Tests the null hypothesis that the observed covariance matrices of the dependent variables are equal across groups.

a. Design: Intercept + groups
 Within Subjects Design: fatigue

Multivariate Tests[a]

Effect		Value	F	Hypothesis df	Error df	Sig.	Partial Eta Squared	Noncent. Parameter	Observed Power[c]
fatigue	Pillai's Trace	.015	.671[b]	1.000	44.000	.417	.015	.671	.126
	Wilks' Lambda	.985	.671[b]	1.000	44.000	.417	.015	.671	.126
	Hotelling's Trace	.015	.671[b]	1.000	44.000	.417	.015	.671	.126
	Roy's Largest Root	.015	.671[b]	1.000	44.000	.417	.015	.671	.126
fatigue * groups	Pillai's Trace	.035	.529[b]	3.000	44.000	.665	.035	1.588	.149
	Wilks' Lambda	.965	.529[b]	3.000	44.000	.665	.035	1.588	.149

	Hotelling's Trace	.036	.529[b]	3.000	44.000	.665	.035	1.588	.149
	Roy's Largest Root	.036	.529[b]	3.000	44.000	.665	.035	1.588	.149

a. Design: Intercept + groups
Within Subjects Design: fatigue
b. Exact statistic
c. Computed using alpha = .05

Mauchly's Test of Sphericity[a]

Measure: MEASURE_1

Within Subjects Effect	Mauchly's W	Approx. Chi-Square	df	Sig.	Epsilon[b]		
					Greenhouse-Geisser	Huynh-Feldt	Lower-bound
fatigue	1.000	.000	0	.	1.000	1.000	1.000

Tests the null hypothesis that the error covariance matrix of the orthonormalized transformed dependent variables is proportional to an identity matrix.
a. Design: Intercept + groups
Within Subjects Design: fatigue
b. May be used to adjust the degrees of freedom for the averaged tests of significance. Corrected tests are displayed in the Tests of Within-Subjects Effects table.

Tests of Within-Subjects Effects

Measure: MEASURE_1

Source		Type III Sum of Squares	df	Mean Square	F	Sig.	Partial Eta Squared	Noncent. Parameter	Observed Power[a]
fatigue	Sphericity Assumed	124.029	1	124.029	.671	.417	.015	.671	.126
	Greenhouse-Geisser	124.029	1.000	124.029	.671	.417	.015	.671	.126
	Huynh-Feldt	124.029	1.000	124.029	.671	.417	.015	.671	.126
	Lower-bound	124.029	1.000	124.029	.671	.417	.015	.671	.126
fatigue * groups	Sphericity Assumed	293.573	3	97.858	.529	.665	.035	1.588	.149
	Greenhouse-Geisser	293.573	3.000	97.858	.529	.665	.035	1.588	.149
	Huynh-Feldt	293.573	3.000	97.858	.529	.665	.035	1.588	.149
	Lower-bound	293.573	3.000	97.858	.529	.665	.035	1.588	.149
Error(fatigue)	Sphericity Assumed	8135.089	44	184.888					
	Greenhouse-Geisser	8135.089	44.000	184.888					
	Huynh-Feldt	8135.089	44.000	184.888					
	Lower-bound	8135.089	44.000	184.888					

a. Computed using alpha = .05

Tests of Within-Subjects Contrasts

Measure: MEASURE_1

Source	fatigue	Type III Sum of Squares	df	Mean Square	F	Sig.	Partial Eta Squared	Noncent. Parameter	Observed Power[a]
fatigue	Linear	124.029	1	124.029	.671	.417	.015	.671	.126
fatigue * groups	Linear	293.573	3	97.858	.529	.665	.035	1.588	.149
Error(fatigue)	Linear	8135.089	44	184.888					

a. Computed using alpha = .05

Levene's Test of Equality of Error Variances[a]

		Levene Statistic	df1	df2	Sig.
NbfPOpre	Based on Mean	3.422	3	44	.025
	Based on Median	1.943	3	44	.137
	Based on Median and with adjusted df	1.943	3	33.418	.142
	Based on trimmed mean	3.135	3	44	.035
NbfPOpos	Based on Mean	1.499	3	44	.228
	Based on Median	1.068	3	44	.373
	Based on Median and with adjusted df	1.068	3	20.785	.384
	Based on trimmed mean	1.072	3	44	.371

Tests the null hypothesis that the error variance of the dependent variable is equal across groups.

a. Design: Intercept + groups
Within Subjects Design: fatigue

Tests of Between-Subjects Effects

Measure: MEASURE_1
Transformed Variable: Average

Source	Type III Sum of Squares	df	Mean Square	F	Sig.	Partial Eta Squared	Noncent. Parameter	Observed Power[a]
Intercept	23950.758	1	23950.758	90.338	.000	.672	90.338	1.000
groups	4185.012	3	1395.004	5.262	.003	.264	15.785	.906
Error	11665.462	44	265.124					

a. Computed using alpha = .05

Parameter Estimates

Dependent Variable	Parameter	B	Std. Error	t	Sig.	95% Confidence Interval Lower Bound	95% Confidence Interval Upper Bound	Partial Eta Squared	Noncent. Parameter	Observed Power[b]
NbfPOpre	Intercept	5.254	2.572	2.043	.047	.070	10.438	.087	2.043	.515
	[groups=1.00]	10.813	3.835	2.820	.007	3.086	18.541	.153	2.820	.788
	[groups=2.00]	13.125	3.341	3.928	.000	6.391	19.859	.260	3.928	.970
	[groups=3.00]	14.942	3.561	4.196	.000	7.765	22.119	.286	4.196	.984
	[groups=4.00]	0[a]	.	.	.	.	.	.	.	.
NbfPOpos	Intercept	5.071	5.856	.866	.391	-6.732	16.873	.017	.866	.135
	[groups=1.00]	12.929	8.730	1.481	.146	-4.664	30.523	.047	1.481	.305
	[groups=2.00]	20.979	7.607	2.758	.008	5.648	36.311	.147	2.758	.769
	[groups=3.00]	14.996	8.107	1.850	.071	-1.343	31.335	.072	1.850	.440
	[groups=4.00]	0[a]	.	.	.	.	.	.	.	.

a. This parameter is set to zero because it is redundant.

b. Computed using alpha = .05

Estimated Marginal Means

1. Grand Mean

Measure: MEASURE_1

Mean	Std. Error	95% Confidence Interval Lower Bound	95% Confidence Interval Upper Bound
16.136	1.698	12.714	19.557

2. groups

Estimates

Measure: MEASURE_1

groups	Mean	Std. Error	95% Confidence Interval

			Lower Bound	Upper Bound
1.00	17.034	3.838	9.299	24.768
2.00	22.214	2.878	16.413	28.015
3.00	20.131	3.324	13.433	26.830
4.00	5.162	3.471	-1.834	12.159

Pairwise Comparisons

Measure: MEASURE_1

(I) groups	(J) groups	Mean Difference (I-J)	Std. Error	Sig.[b]	95% Confidence Interval for Difference[b] Lower Bound	Upper Bound
1.00	2.00	-5.181	4.797	1.000	-18.435	8.073
	3.00	-3.098	5.077	1.000	-17.125	10.929
	4.00	11.871	5.175	.160	-2.426	26.169
2.00	1.00	5.181	4.797	1.000	-8.073	18.435
	3.00	2.083	4.397	1.000	-10.065	14.231
	4.00	17.052*	4.510	.003	4.593	29.511
3.00	1.00	3.098	5.077	1.000	-10.929	17.125
	2.00	-2.083	4.397	1.000	-14.231	10.065
	4.00	14.969*	4.806	.019	1.691	28.247
4.00	1.00	-11.871	5.175	.160	-26.169	2.426
	2.00	-17.052*	4.510	.003	-29.511	-4.593
	3.00	-14.969*	4.806	.019	-28.247	-1.691

Based on estimated marginal means

*. The mean difference is significant at the .05 level.

b. Adjustment for multiple comparisons: Bonferroni.

Univariate Tests

Measure: MEASURE_1

	Sum of Squares	df	Mean Square	F	Sig.	Partial Eta Squared	Noncent. Parameter	Observed Power[a]
Contrast	2092.506	3	697.502	5.262	.003	.264	15.785	.906
Error	5832.731	44	132.562					

The F tests the effect of groups. This test is based on the linearly independent pairwise comparisons among the estimated marginal means.

a. Computed using alpha = .05

3. fatigue

Estimates

Measure: MEASURE_1

fatigue	Mean	Std. Error	95% Confidence Interval Lower Bound	95% Confidence Interval Upper Bound
1	14.974	1.258	12.439	17.510
2	17.297	2.864	11.525	23.068

Pairwise Comparisons

Measure: MEASURE_1

(I) fatigue	(J) fatigue	Mean Difference (I-J)	Std. Error	Sig.[a]	95% Confidence Interval for Difference[a] Lower Bound	95% Confidence Interval for Difference[a] Upper Bound
1	2	-2.322	2.835	.417	-8.037	3.392
2	1	2.322	2.835	.417	-3.392	8.037

Based on estimated marginal means

a. Adjustment for multiple comparisons: Bonferroni.

Multivariate Tests

	Value	F	Hypothesis df	Error df	Sig.	Partial Eta Squared	Noncent. Parameter	Observed Power[b]
Pillai's trace	.015	.671[a]	1.000	44.000	.417	.015	.671	.126
Wilks' lambda	.985	.671[a]	1.000	44.000	.417	.015	.671	.126
Hotelling's trace	.015	.671[a]	1.000	44.000	.417	.015	.671	.126
Roy's largest root	.015	.671[a]	1.000	44.000	.417	.015	.671	.126

Each F tests the multivariate effect of fatigue. These tests are based on the linearly independent pairwise comparisons among the estimated marginal means.

a. Exact statistic

b. Computed using alpha = .05

4. groups * fatigue

Measure: MEASURE_1

groups	fatigue	Mean	Std. Error	95% Confidence Interval Lower Bound	95% Confidence Interval Upper Bound
1.00	1	16.068	2.844	10.336	21.799

	2	18.000	6.474	4.952	31.048
2.00	1	18.379	2.133	14.081	22.678
	2	26.050	4.856	16.264	35.835
3.00	1	20.196	2.463	15.233	25.160
	2	20.066	5.607	8.767	31.366
4.00	1	5.254	2.572	.070	10.438
	2	5.071	5.856	-6.732	16.873

Post Hoc Tests

groups

Multiple Comparisons

Measure: MEASURE_1
Tukey HSD

(I) groups	(J) groups	Mean Difference (I-J)	Std. Error	Sig.	95% Confidence Interval	
					Lower Bound	Upper Bound
1.00	2.00	-5.1807	4.79732	.703	-17.9896	7.6282
	3.00	-3.0977	5.07700	.928	-16.6533	10.4579
	4.00	11.8714	5.17496	.115	-1.9458	25.6886
2.00	1.00	5.1807	4.79732	.703	-7.6282	17.9896
	3.00	2.0830	4.39681	.964	-9.6565	13.8225
	4.00	17.0521*	4.50957	.003	5.0115	29.0927
3.00	1.00	3.0977	5.07700	.928	-10.4579	16.6533
	2.00	-2.0830	4.39681	.964	-13.8225	9.6565
	4.00	14.9691*	4.80603	.016	2.1369	27.8012
4.00	1.00	-11.8714	5.17496	.115	-25.6886	1.9458
	2.00	-17.0521*	4.50957	.003	-29.0927	-5.0115
	3.00	-14.9691*	4.80603	.016	-27.8012	-2.1369

Based on observed means.
The error term is Mean Square(Error) = 132.562.

*. The mean difference is significant at the .05 level.

Homogeneous Subsets

MEASURE_1

Tukey HSD[a,b,c]

groups	N	Subset	
		1	2
4.00	15	5.1624	
1.00	10	17.0338	17.0338
3.00	12		20.1315
2.00	16		22.2145
Sig.		.079	.704

Means for groups in homogeneous subsets are displayed.
Based on observed means.
The error term is Mean Square(Error) = 132.562.
a. Uses Harmonic Mean Sample Size = 11.499.
b. The group sizes are unequal. The harmonic mean of the group sizes is used. Type I error levels are not guaranteed.
c. Alpha = .05.

2.7. Semitendinosus (ST) - Late Stance

```
GLM NstPOpre NstPOpos BY groups
  /WSFACTOR=fatigue 2 Polynomial
  /METHOD=SSTYPE(3)
  /POSTHOC=groups(TUKEY)
  /EMMEANS=TABLES(OVERALL)
  /EMMEANS=TABLES(groups) COMPARE ADJ(BONFERRONI)
  /EMMEANS=TABLES(fatigue) COMPARE ADJ(BONFERRONI)
  /EMMEANS=TABLES(groups*fatigue)
  /PRINT=DESCRIPTIVE ETASQ OPOWER PARAMETER HOMOGENEITY
  /CRITERIA=ALPHA(.05)
  /WSDESIGN=fatigue
  /DESIGN=groups.
```

General Linear Model

Notes

Output Created		17-MAY-2026 21:32:52
Comments		
Input	Data	C:\Users\RaSa\Desktop\پروپوزال\New folder\data\emgmi1.sav
	Active Dataset	DataSet2
	Filter	<none>
	Weight	<none>
	Split File	<none>
	N of Rows in Working Data File	48
Missing Value Handling	Definition of Missing	User-defined missing values are treated as missing.
	Cases Used	Statistics are based on all cases with valid data for all variables in the model.

Syntax		GLM NstPOpre NstPOpos BY groups<br>/WSFACTOR=fatigue 2 Polynomial<br>/METHOD=SSTYPE(3)<br>/POSTHOC=groups(TUKEY)<br>/EMMEANS=TABLES(OVERALL)<br>/EMMEANS=TABLES(groups) COMPARE ADJ(BONFERRONI)<br>/EMMEANS=TABLES(fatigue) COMPARE ADJ(BONFERRONI)<br>/EMMEANS=TABLES(groups*fatigue)<br>/PRINT=DESCRIPTIVE ETASQ OPOWER PARAMETER HOMOGENEITY<br>/CRITERIA=ALPHA(.05)<br>/WSDESIGN=fatigue<br>/DESIGN=groups.
Resources	Processor Time	00:00:00.02
	Elapsed Time	00:00:00.03

Within-Subjects Factors

Measure: MEASURE_1

fatigue	Dependent Variable
1	NstPOpre
2	NstPOpos

Between-Subjects Factors

		N
groups	1.00	10
	2.00	16
	3.00	12
	4.00	15

Descriptive Statistics

	groups	Mean	Std. Deviation	N
NstPOpre	1.00	15.7948	7.98246	10
	2.00	13.9572	9.19707	16
	3.00	18.7256	10.62199	12
	4.00	5.3940	5.00684	15
	Total	13.5315	9.64418	53
NstPOpos	1.00	15.2493	8.07192	10
	2.00	12.0656	6.55330	16
	3.00	13.1932	5.27058	12
	4.00	4.6354	4.36295	15
	Total	11.2417	7.05982	53

Box's Test of Equality of Covariance Matrices[a]

Box's M	21.466
F	2.177
df1	9
df2	12234.839
Sig.	.021

Tests the null hypothesis that the observed covariance matrices of the dependent variables are equal across groups.
a. Design: Intercept + groups
Within Subjects Design: fatigue

Multivariate Tests[a]

Effect		Value	F	Hypothesis df	Error df	Sig.	Partial Eta Squared	Noncent. Parameter	Observed Power[c]
fatigue	Pillai's Trace	.059	2.780[b]	1.000	44.000	.103	.059	2.780	.371
	Wilks' Lambda	.941	2.780[b]	1.000	44.000	.103	.059	2.780	.371
	Hotelling's Trace	.063	2.780[b]	1.000	44.000	.103	.059	2.780	.371
	Roy's Largest Root	.063	2.780[b]	1.000	44.000	.103	.059	2.780	.371
fatigue * groups	Pillai's Trace	.050	.770[b]	3.000	44.000	.517	.050	2.309	.202
	Wilks' Lambda	.950	.770[b]	3.000	44.000	.517	.050	2.309	.202

Hotelling's Trace	.052	.770[b]	3.000	44.000	.517	.050	2.309	.202
Roy's Largest Root	.052	.770[b]	3.000	44.000	.517	.050	2.309	.202

a. Design: Intercept + groups
Within Subjects Design: fatigue
b. Exact statistic
c. Computed using alpha = .05

Mauchly's Test of Sphericity[a]

Measure: MEASURE_1

Within Subjects Effect	Mauchly's W	Approx. Chi-Square	df	Sig.	Epsilon[b] Greenhouse-Geisser	Huynh-Feldt	Lower-bound
fatigue	1.000	.000	0	.	1.000	1.000	1.000

Tests the null hypothesis that the error covariance matrix of the orthonormalized transformed dependent variables is proportional to an identity matrix.
a. Design: Intercept + groups
Within Subjects Design: fatigue
b. May be used to adjust the degrees of freedom for the averaged tests of significance. Corrected tests are displayed in the Tests of Within-Subjects Effects table.

Tests of Within-Subjects Effects

Measure: MEASURE_1

Source		Type III Sum of Squares	df	Mean Square	F	Sig.	Partial Eta Squared	Noncent. Parameter	Observed Power[a]
fatigue	Sphericity Assumed	109.499	1	109.499	2.780	.103	.059	2.780	.371
	Greenhouse-Geisser	109.499	1.000	109.499	2.780	.103	.059	2.780	.371
	Huynh-Feldt	109.499	1.000	109.499	2.780	.103	.059	2.780	.371
	Lower-bound	109.499	1.000	109.499	2.780	.103	.059	2.780	.371
fatigue * groups	Sphericity Assumed	90.943	3	30.314	.770	.517	.050	2.309	.202
	Greenhouse-Geisser	90.943	3.000	30.314	.770	.517	.050	2.309	.202
	Huynh-Feldt	90.943	3.000	30.314	.770	.517	.050	2.309	.202
	Lower-bound	90.943	3.000	30.314	.770	.517	.050	2.309	.202
Error(fatigue)	Sphericity Assumed	1733.085	44	39.388					
	Greenhouse-Geisser	1733.085	44.000	39.388					
	Huynh-Feldt	1733.085	44.000	39.388					
	Lower-bound	1733.085	44.000	39.388					

a. Computed using alpha = .05

Tests of Within-Subjects Contrasts

Measure: MEASURE_1

Source	fatigue	Type III Sum of Squares	df	Mean Square	F	Sig.	Partial Eta Squared	Noncent. Parameter	Observed Power[a]
fatigue	Linear	109.499	1	109.499	2.780	.103	.059	2.780	.371
fatigue * groups	Linear	90.943	3	30.314	.770	.517	.050	2.309	.202
Error(fatigue)	Linear	1733.085	44	39.388					

a. Computed using alpha = .05

Levene's Test of Equality of Error Variances[a]

		Levene Statistic	df1	df2	Sig.
NstPOpre	Based on Mean	2.165	3	44	.106
	Based on Median	1.648	3	44	.192
	Based on Median and with adjusted df	1.648	3	37.828	.195
	Based on trimmed mean	2.131	3	44	.110
NstPOpos	Based on Mean	1.869	3	44	.149
	Based on Median	1.597	3	44	.204
	Based on Median and with adjusted df	1.597	3	35.802	.207
	Based on trimmed mean	1.908	3	44	.142

Tests the null hypothesis that the error variance of the dependent variable is equal across groups.

a. Design: Intercept + groups
Within Subjects Design: fatigue

Tests of Between-Subjects Effects

Measure: MEASURE_1
Transformed Variable: Average

Source	Type III Sum of Squares	df	Mean Square	F	Sig.	Partial Eta Squared	Noncent. Parameter	Observed Power[a]
Intercept	14092.141	1	14092.141	193.852	.000	.815	193.852	1.000
groups	1691.392	3	563.797	7.756	.000	.346	23.267	.982
Error	3198.595	44	72.695					

a. Computed using alpha = .05

Parameter Estimates

Dependent Variable	Parameter	B	Std. Error	t	Sig.	95% Confidence Interval Lower Bound	95% Confidence Interval Upper Bound	Partial Eta Squared	Noncent. Parameter	Observed Power[b]
NstPOpre	Intercept	5.394	2.599	2.075	.044	.155	10.633	.089	2.075	.528
	[groups=1.00]	10.401	3.875	2.684	.010	2.591	18.210	.141	2.684	.747
	[groups=2.00]	8.563	3.377	2.536	.015	1.758	15.369	.128	2.536	.699
	[groups=3.00]	13.332	3.599	3.705	.001	6.079	20.584	.238	3.705	.952
	[groups=4.00]	0[a]	.	.	.	.	.	.	.	.
NstPOpos	Intercept	4.635	1.853	2.502	.016	.902	8.369	.125	2.502	.687
	[groups=1.00]	10.614	2.762	3.843	.000	5.048	16.180	.251	3.843	.964
	[groups=2.00]	7.430	2.407	3.087	.003	2.580	12.281	.178	3.087	.855
	[groups=3.00]	8.558	2.565	3.336	.002	3.388	13.727	.202	3.336	.904
	[groups=4.00]	0[a]	.	.	.	.	.	.	.	.

a. This parameter is set to zero because it is redundant.

b. Computed using alpha = .05

Estimated Marginal Means

1. Grand Mean

Measure: MEASURE_1

Mean	Std. Error	95% Confidence Interval Lower Bound	95% Confidence Interval Upper Bound
12.377	.889	10.585	14.168

2. groups

Estimates

Measure: MEASURE_1

groups	Mean	Std. Error	95% Confidence Interval

			Lower Bound	Upper Bound
1.00	15.522	2.010	11.472	19.572
2.00	13.011	1.507	9.974	16.049
3.00	15.959	1.740	12.452	19.467
4.00	5.015	1.818	1.351	8.678

Pairwise Comparisons

Measure: MEASURE_1

					95% Confidence Interval for Difference[b]	
(I) groups	(J) groups	Mean Difference (I-J)	Std. Error	Sig.[b]	Lower Bound	Upper Bound
1.00	2.00	2.511	2.512	1.000	-4.430	9.451
	3.00	-.437	2.658	1.000	-7.782	6.908
	4.00	10.507*	2.710	.002	3.021	17.994
2.00	1.00	-2.511	2.512	1.000	-9.451	4.430
	3.00	-2.948	2.302	1.000	-9.309	3.413
	4.00	7.997*	2.361	.009	1.473	14.521
3.00	1.00	.437	2.658	1.000	-6.908	7.782
	2.00	2.948	2.302	1.000	-3.413	9.309
	4.00	10.945*	2.517	.000	3.992	17.898
4.00	1.00	-10.507*	2.710	.002	-17.994	-3.021
	2.00	-7.997*	2.361	.009	-14.521	-1.473
	3.00	-10.945*	2.517	.000	-17.898	-3.992

Based on estimated marginal means

*. The mean difference is significant at the .05 level.

b. Adjustment for multiple comparisons: Bonferroni.

Univariate Tests

Measure: MEASURE_1

	Sum of Squares	df	Mean Square	F	Sig.	Partial Eta Squared	Noncent. Parameter	Observed Power[a]
Contrast	845.696	3	281.899	7.756	.000	.346	23.267	.982
Error	1599.297	44	36.348					

The F tests the effect of groups. This test is based on the linearly independent pairwise comparisons among the estimated marginal means.

a. Computed using alpha = .05

3. fatigue

Estimates

Measure: MEASURE_1

fatigue	Mean	Std. Error	95% Confidence Interval	
			Lower Bound	Upper Bound
1	13.468	1.271	10.906	16.030
2	11.286	.906	9.460	13.112

Pairwise Comparisons

Measure: MEASURE_1

(I) fatigue	(J) fatigue	Mean Difference (I-J)	Std. Error	Sig.[a]	95% Confidence Interval for Difference[a]	
					Lower Bound	Upper Bound
1	2	2.182	1.309	.103	-.455	4.820
2	1	-2.182	1.309	.103	-4.820	.455

Based on estimated marginal means

a. Adjustment for multiple comparisons: Bonferroni.

Multivariate Tests

	Value	F	Hypothesis df	Error df	Sig.	Partial Eta Squared	Noncent. Parameter	Observed Power[b]
Pillai's trace	.059	2.780[a]	1.000	44.000	.103	.059	2.780	.371
Wilks' lambda	.941	2.780[a]	1.000	44.000	.103	.059	2.780	.371
Hotelling's trace	.063	2.780[a]	1.000	44.000	.103	.059	2.780	.371
Roy's largest root	.063	2.780[a]	1.000	44.000	.103	.059	2.780	.371

Each F tests the multivariate effect of fatigue. These tests are based on the linearly independent pairwise comparisons among the estimated marginal means.

a. Exact statistic

b. Computed using alpha = .05

4. groups * fatigue

Measure: MEASURE_1

groups	fatigue	Mean	Std. Error	95% Confidence Interval	
				Lower Bound	Upper Bound
1.00	1	15.795	2.874	10.003	21.586

	2	15.249	2.048	11.121	19.377
2.00	1	13.957	2.155	9.613	18.301
	2	12.066	1.536	8.970	15.162
3.00	1	18.726	2.489	13.710	23.741
	2	13.193	1.774	9.618	16.768
4.00	1	5.394	2.599	.155	10.633
	2	4.635	1.853	.902	8.369

Post Hoc Tests

groups

Multiple Comparisons

Measure: MEASURE_1
Tukey HSD

(I) groups	(J) groups	Mean Difference (I-J)	Std. Error	Sig.	95% Confidence Interval Lower Bound	Upper Bound
1.00	2.00	2.5106	2.51204	.750	-4.1966	9.2178
	3.00	-.4374	2.65850	.998	-7.5356	6.6608
	4.00	10.5073*	2.70979	.002	3.2721	17.7425
2.00	1.00	-2.5106	2.51204	.750	-9.2178	4.1966
	3.00	-2.9480	2.30233	.580	-9.0952	3.1992
	4.00	7.9967*	2.36137	.008	1.6918	14.3016
3.00	1.00	.4374	2.65850	.998	-6.6608	7.5356
	2.00	2.9480	2.30233	.580	-3.1992	9.0952
	4.00	10.9447*	2.51661	.000	4.2254	17.6641
4.00	1.00	-10.5073*	2.70979	.002	-17.7425	-3.2721
	2.00	-7.9967*	2.36137	.008	-14.3016	-1.6918
	3.00	-10.9447*	2.51661	.000	-17.6641	-4.2254

Based on observed means.
The error term is Mean Square(Error) = 36.348.

*. The mean difference is significant at the .05 level.

Homogeneous Subsets

MEASURE_1

Tukey HSD[a,b,c]

groups	N	Subset 1	Subset 2
4.00	15	5.0147	
2.00	16		13.0114
1.00	10		15.5220
3.00	12		15.9594
Sig.		1.000	.647

Means for groups in homogeneous subsets are displayed.
Based on observed means.
The error term is Mean Square(Error) = 36.348.
a. Uses Harmonic Mean Sample Size = 11.499.
b. The group sizes are unequal. The harmonic mean of the group sizes is used. Type I error levels are not guaranteed.
c. Alpha = .05.

2.8. Gluteus Medius (GM) - Late Stance

```
GLM NgmPOpre NgmPOpos BY groups
  /WSFACTOR=fatigue 2 Polynomial
  /METHOD=SSTYPE(3)
  /POSTHOC=groups(TUKEY)
  /EMMEANS=TABLES(OVERALL)
  /EMMEANS=TABLES(groups) COMPARE ADJ(BONFERRONI)
  /EMMEANS=TABLES(fatigue) COMPARE ADJ(BONFERRONI)
  /EMMEANS=TABLES(groups*fatigue)
  /PRINT=DESCRIPTIVE ETASQ OPOWER PARAMETER HOMOGENEITY
  /CRITERIA=ALPHA(.05)
  /WSDESIGN=fatigue
  /DESIGN=groups.
```

General Linear Model

Notes

Output Created		17-MAY-2026 21:33:22
Comments		
Input	Data	C:\Users\RaSa\Desktop\پروپوزال\New folder\data\emgmi1.sav
	Active Dataset	DataSet2
	Filter	<none>
	Weight	<none>
	Split File	<none>
	N of Rows in Working Data File	48
Missing Value Handling	Definition of Missing	User-defined missing values are treated as missing.
	Cases Used	Statistics are based on all cases with valid data for all variables in the model.

Syntax		GLM NgmPOpre NgmPOpos BY groups<br>/WSFACTOR=fatigue 2 Polynomial<br>/METHOD=SSTYPE(3)<br>/POSTHOC=groups(TUKEY)<br>/EMMEANS=TABLES(OVERALL)<br>/EMMEANS=TABLES(groups) COMPARE ADJ(BONFERRONI)<br>/EMMEANS=TABLES(fatigue) COMPARE ADJ(BONFERRONI)<br>/EMMEANS=TABLES(groups*fatigue)<br>/PRINT=DESCRIPTIVE ETASQ OPOWER PARAMETER HOMOGENEITY<br>/CRITERIA=ALPHA(.05)<br>/WSDESIGN=fatigue<br>/DESIGN=groups.
Resources	Processor Time	00:00:00.05
	Elapsed Time	00:00:00.03

Within-Subjects Factors

Measure: MEASURE_1

fatigue	Dependent Variable
1	NgmPOpre
2	NgmPOpos

Between-Subjects Factors

		N
groups	1.00	10
	2.00	16
	3.00	12
	4.00	15

Descriptive Statistics

	groups	Mean	Std. Deviation	N
NgmPOpre	1.00	22.7015	15.06559	10
	2.00	26.3438	18.06969	16
	3.00	20.7620	10.52049	12
	4.00	12.2702	5.11139	15
	Total	21.0402	14.22769	53
NgmPOpos	1.00	25.5619	10.22534	10
	2.00	27.0590	16.22235	16
	3.00	14.7740	7.71023	12
	4.00	16.6392	10.18491	15
	Total	21.3192	12.98061	53

Box's Test of Equality of Covariance Matrices[a]

Box's M	22.799
F	2.312
df1	9
df2	12234.839
Sig.	.014

Tests the null hypothesis that the observed covariance matrices of the dependent variables are equal across groups.
a. Design: Intercept + groups
Within Subjects Design: fatigue

Multivariate Tests[a]

Effect		Value	F	Hypothesis df	Error df	Sig.	Partial Eta Squared	Noncent. Parameter	Observed Power[c]
fatigue	Pillai's Trace	.001	.061[b]	1.000	44.000	.806	.001	.061	.057
	Wilks' Lambda	.999	.061[b]	1.000	44.000	.806	.001	.061	.057
	Hotelling's Trace	.001	.061[b]	1.000	44.000	.806	.001	.061	.057
	Roy's Largest Root	.001	.061[b]	1.000	44.000	.806	.001	.061	.057
fatigue * groups	Pillai's Trace	.083	1.331[b]	3.000	44.000	.277	.083	3.992	.330
	Wilks' Lambda	.917	1.331[b]	3.000	44.000	.277	.083	3.992	.330

	Hotelling's Trace	.091	1.331[b]	3.000	44.000	.277	.083	3.992	.330
	Roy's Largest Root	.091	1.331[b]	3.000	44.000	.277	.083	3.992	.330

a. Design: Intercept + groups
Within Subjects Design: fatigue
b. Exact statistic
c. Computed using alpha = .05

Mauchly's Test of Sphericity[a]

Measure: MEASURE_1

Within Subjects Effect	Mauchly's W	Approx. Chi-Square	df	Sig.	Epsilon[b]		
					Greenhouse-Geisser	Huynh-Feldt	Lower-bound
fatigue	1.000	.000	0	.	1.000	1.000	1.000

Tests the null hypothesis that the error covariance matrix of the orthonormalized transformed dependent variables is proportional to an identity matrix.
a. Design: Intercept + groups
Within Subjects Design: fatigue
b. May be used to adjust the degrees of freedom for the averaged tests of significance. Corrected tests are displayed in the Tests of Within-Subjects Effects table.

Tests of Within-Subjects Effects

Measure: MEASURE_1

Source		Type III Sum of Squares	df	Mean Square	F	Sig.	Partial Eta Squared	Noncent. Parameter	Observed Power[a]
fatigue	Sphericity Assumed	5.503	1	5.503	.061	.806	.001	.061	.057
	Greenhouse-Geisser	5.503	1.000	5.503	.061	.806	.001	.061	.057
	Huynh-Feldt	5.503	1.000	5.503	.061	.806	.001	.061	.057
	Lower-bound	5.503	1.000	5.503	.061	.806	.001	.061	.057
fatigue * groups	Sphericity Assumed	359.162	3	119.721	1.331	.277	.083	3.992	.330
	Greenhouse-Geisser	359.162	3.000	119.721	1.331	.277	.083	3.992	.330
	Huynh-Feldt	359.162	3.000	119.721	1.331	.277	.083	3.992	.330
	Lower-bound	359.162	3.000	119.721	1.331	.277	.083	3.992	.330
Error(fatigue)	Sphericity Assumed	3958.933	44	89.976					
	Greenhouse-Geisser	3958.933	44.000	89.976					
	Huynh-Feldt	3958.933	44.000	89.976					
	Lower-bound	3958.933	44.000	89.976					

a. Computed using alpha = .05

Tests of Within-Subjects Contrasts

Measure: MEASURE_1

Source	fatigue	Type III Sum of Squares	df	Mean Square	F	Sig.	Partial Eta Squared	Noncent. Parameter	Observed Power[a]
fatigue	Linear	5.503	1	5.503	.061	.806	.001	.061	.057
fatigue * groups	Linear	359.162	3	119.721	1.331	.277	.083	3.992	.330
Error(fatigue)	Linear	3958.933	44	89.976					

a. Computed using alpha = .05

Levene's Test of Equality of Error Variances[a]

		Levene Statistic	df1	df2	Sig.
NgmPOpre	Based on Mean	6.324	3	44	.001
	Based on Median	1.920	3	44	.140
	Based on Median and with adjusted df	1.920	3	32.268	.146
	Based on trimmed mean	5.287	3	44	.003
NgmPOpos	Based on Mean	1.251	3	44	.303
	Based on Median	1.091	3	44	.363
	Based on Median and with adjusted df	1.091	3	30.872	.368
	Based on trimmed mean	1.101	3	44	.359

Tests the null hypothesis that the error variance of the dependent variable is equal across groups.

a. Design: Intercept + groups
Within Subjects Design: fatigue

Tests of Between-Subjects Effects

Measure: MEASURE_1
Transformed Variable: Average

Source	Type III Sum of Squares	df	Mean Square	F	Sig.	Partial Eta Squared	Noncent. Parameter	Observed Power[a]
Intercept	39661.859	1	39661.859	162.966	.000	.787	162.966	1.000
groups	2406.836	3	802.279	3.296	.029	.184	9.889	.714
Error	10708.475	44	243.374					

a. Computed using alpha = .05

Parameter Estimates

Dependent Variable	Parameter	B	Std. Error	t	Sig.	95% Confidence Interval Lower Bound	95% Confidence Interval Upper Bound	Partial Eta Squared	Noncent. Parameter	Observed Power[b]
NgmPOpre	Intercept	12.270	4.114	2.982	.005	3.979	20.562	.168	2.982	.831
	[groups=1.00]	10.431	6.133	1.701	.096	-1.929	22.791	.062	1.701	.384
	[groups=2.00]	14.074	5.344	2.633	.012	3.303	24.845	.136	2.633	.731
	[groups=3.00]	8.492	5.696	1.491	.143	-2.987	19.971	.048	1.491	.308
	[groups=4.00]	0[a]	.	.	.	.	.	.	.	.
NgmPOpos	Intercept	16.639	3.658	4.549	.000	9.268	24.011	.320	4.549	.994
	[groups=1.00]	8.923	5.453	1.636	.109	-2.066	19.912	.057	1.636	.360
	[groups=2.00]	10.420	4.751	2.193	.034	.844	19.996	.099	2.193	.573
	[groups=3.00]	-1.865	5.064	-.368	.714	-12.071	8.340	.003	.368	.065
	[groups=4.00]	0[a]	.	.	.	.	.	.	.	.

a. This parameter is set to zero because it is redundant.

b. Computed using alpha = .05

Estimated Marginal Means

1. Grand Mean

Measure: MEASURE_1

Mean	Std. Error	95% Confidence Interval Lower Bound	95% Confidence Interval Upper Bound
20.764	1.627	17.486	24.042

2. groups

Estimates

Measure: MEASURE_1

groups	Mean	Std. Error	95% Confidence Interval

			Lower Bound	Upper Bound
1.00	24.132	3.677	16.721	31.542
2.00	26.701	2.758	21.143	32.259
3.00	17.768	3.184	11.350	24.186
4.00	14.455	3.326	7.752	21.158

Pairwise Comparisons

Measure: MEASURE_1

(I) groups	(J) groups	Mean Difference (I-J)	Std. Error	Sig.[b]	95% Confidence Interval for Difference[b] Lower Bound	Upper Bound
1.00	2.00	-2.570	4.596	1.000	-15.268	10.129
	3.00	6.364	4.864	1.000	-7.075	19.803
	4.00	9.677	4.958	.344	-4.021	23.375
2.00	1.00	2.570	4.596	1.000	-10.129	15.268
	3.00	8.933	4.213	.238	-2.705	20.572
	4.00	12.247*	4.321	.041	.310	24.184
3.00	1.00	-6.364	4.864	1.000	-19.803	7.075
	2.00	-8.933	4.213	.238	-20.572	2.705
	4.00	3.313	4.605	1.000	-9.409	16.035
4.00	1.00	-9.677	4.958	.344	-23.375	4.021
	2.00	-12.247*	4.321	.041	-24.184	-.310
	3.00	-3.313	4.605	1.000	-16.035	9.409

Based on estimated marginal means
*. The mean difference is significant at the .05 level.
b. Adjustment for multiple comparisons: Bonferroni.

Univariate Tests

Measure: MEASURE_1

	Sum of Squares	df	Mean Square	F	Sig.	Partial Eta Squared	Noncent. Parameter	Observed Power[a]
Contrast	1203.418	3	401.139	3.296	.029	.184	9.889	.714
Error	5354.238	44	121.687					

The F tests the effect of groups. This test is based on the linearly independent pairwise comparisons among the estimated marginal means.
a. Computed using alpha = .05

3. fatigue

Estimates

Measure: MEASURE_1

fatigue	Mean	Std. Error	95% Confidence Interval Lower Bound	95% Confidence Interval Upper Bound
1	20.519	2.012	16.465	24.574
2	21.009	1.789	17.404	24.613

Pairwise Comparisons

Measure: MEASURE_1

(I) fatigue	(J) fatigue	Mean Difference (I-J)	Std. Error	Sig.[a]	95% Confidence Interval for Difference[a] Lower Bound	95% Confidence Interval for Difference[a] Upper Bound
1	2	-.489	1.978	.806	-4.475	3.497
2	1	.489	1.978	.806	-3.497	4.475

Based on estimated marginal means

a. Adjustment for multiple comparisons: Bonferroni.

Multivariate Tests

	Value	F	Hypothesis df	Error df	Sig.	Partial Eta Squared	Noncent. Parameter	Observed Power[b]
Pillai's trace	.001	.061[a]	1.000	44.000	.806	.001	.061	.057
Wilks' lambda	.999	.061[a]	1.000	44.000	.806	.001	.061	.057
Hotelling's trace	.001	.061[a]	1.000	44.000	.806	.001	.061	.057
Roy's largest root	.001	.061[a]	1.000	44.000	.806	.001	.061	.057

Each F tests the multivariate effect of fatigue. These tests are based on the linearly independent pairwise comparisons among the estimated marginal means.

a. Exact statistic

b. Computed using alpha = .05

4. groups * fatigue

Measure: MEASURE_1

groups	fatigue	Mean	Std. Error	95% Confidence Interval Lower Bound	95% Confidence Interval Upper Bound
1.00	1	22.701	4.548	13.535	31.868

	2	25.562	4.044	17.412	33.711
2.00	1	26.344	3.411	19.469	33.219
	2	27.059	3.033	20.947	33.171
3.00	1	20.762	3.939	12.823	28.700
	2	14.774	3.502	7.716	21.832
4.00	1	12.270	4.114	3.979	20.562
	2	16.639	3.658	9.268	24.011

Post Hoc Tests

groups

Multiple Comparisons

Measure: MEASURE_1
Tukey HSD

(I) groups	(J) groups	Mean Difference (I-J)	Std. Error	Sig.	95% Confidence Interval Lower Bound	Upper Bound
1.00	2.00	-2.5697	4.59633	.944	-14.8419	9.7025
	3.00	6.3637	4.86430	.563	-6.6240	19.3514
	4.00	9.6770	4.95815	.222	-3.5613	22.9153
2.00	1.00	2.5697	4.59633	.944	-9.7025	14.8419
	3.00	8.9334	4.21261	.163	-2.3143	20.1811
	4.00	12.2467*	4.32064	.034	.7106	23.7828
3.00	1.00	-6.3637	4.86430	.563	-19.3514	6.6240
	2.00	-8.9334	4.21261	.163	-20.1811	2.3143
	4.00	3.3133	4.60468	.889	-8.9812	15.6078
4.00	1.00	-9.6770	4.95815	.222	-22.9153	3.5613
	2.00	-12.2467*	4.32064	.034	-23.7828	-.7106
	3.00	-3.3133	4.60468	.889	-15.6078	8.9812

Based on observed means.
The error term is Mean Square(Error) = 121.687.

*. The mean difference is significant at the .05 level.

Homogeneous Subsets

MEASURE_1

Tukey HSD[a,b,c]

groups	N	Subset 1
4.00	15	14.4547
3.00	12	17.7680
1.00	10	24.1317
2.00	16	26.7014
Sig.		.051

Means for groups in homogeneous subsets are displayed.
Based on observed means.
The error term is Mean Square(Error) = 121.687.
a. Uses Harmonic Mean Sample Size = 11.499.
b. The group sizes are unequal. The harmonic mean of the group sizes is used. Type I error levels are not guaranteed.
c. Alpha = .05.

Section 3: Spatiotemporal Parameter Outputs

3.1. Overall Stance Time

```
GLM stancetimepre stancetimepos BY groups
  /WSFACTOR=fatique 2 Polynomial
  /METHOD=SSTYPE(3)
  /POSTHOC=groups(TUKEY)
  /EMMEANS=TABLES(OVERALL)
  /EMMEANS=TABLES(groups) COMPARE ADJ(BONFERRONI)
  /EMMEANS=TABLES(fatique) COMPARE ADJ(BONFERRONI)
  /EMMEANS=TABLES(groups*fatique)
  /PRINT=DESCRIPTIVE ETASQ OPOWER PARAMETER HOMOGENEITY
  /CRITERIA=ALPHA(.05)
  /WSDESIGN=fatique
  /DESIGN=groups.
```

General Linear Model

Notes

Output Created		23-MAR-2026 22:16:22
Comments		
Input	Data	C:\Users\RaSa\Desktop\پروپوزال\New folder\grf4.sav
	Active Dataset	DataSet2
	Filter	<none>
	Weight	<none>
	Split File	<none>
	N of Rows in Working Data File	57
Missing Value Handling	Definition of Missing	User-defined missing values are treated as missing.
	Cases Used	Statistics are based on all cases with valid data for all variables in the model.

Syntax		GLM stancetimepre stancetimepos BY groups<br>/WSFACTOR=fatique 2 Polynomial<br>/METHOD=SSTYPE(3)<br>/POSTHOC=groups(TUKEY)<br>/EMMEANS=TABLES(OVERALL)<br>/EMMEANS=TABLES(groups) COMPARE ADJ(BONFERRONI)<br>/EMMEANS=TABLES(fatique) COMPARE ADJ(BONFERRONI)<br>/EMMEANS=TABLES(groups*fatique)<br>/PRINT=DESCRIPTIVE ETASQ OPOWER PARAMETER HOMOGENEITY<br>/CRITERIA=ALPHA(.05)<br>/WSDESIGN=fatique<br>/DESIGN=groups.
Resources	Processor Time	00:00:00.03
	Elapsed Time	00:00:00.03

Within-Subjects Factors

Measure: MEASURE_1

fatique	Dependent Variable
1	stancetimepre
2	stancetimepos

Between-Subjects Factors

		N
groups	1.00	10
	2.00	16
	3.00	12
	4.00	15

Descriptive Statistics

	groups	Mean	Std. Deviation	N
stancetimepre	1.00	352.5185	61.81067	10
	2.00	338.6458	40.07834	16
	3.00	328.4167	42.93926	12
	4.00	321.1818	32.92495	15
	Total	334.6875	44.09159	53
stancetimepos	1.00	366.5556	58.24207	10
	2.00	319.3542	40.36564	16
	3.00	338.6111	57.60767	12
	4.00	324.7273	36.54945	15
	Total	334.2500	49.59631	53

Box's Test of Equality of Covariance Matrices[a]

Box's M	7.291
F	.739
df1	9
df2	12234.839
Sig.	.673

Tests the null hypothesis that the observed covariance matrices of the dependent variables are equal across groups.

a. Design: Intercept + groups
Within Subjects Design: fatique

Multivariate Tests[a]

Effect		Value	F	Hypothesis df	Error df	Sig.	Partial Eta Squared	Noncent. Parameter	Observed Power[c]
fatique	Pillai's Trace	.005	.200[b]	1.000	44.000	.657	.005	.200	.072
	Wilks' Lambda	.995	.200[b]	1.000	44.000	.657	.005	.200	.072
	Hotelling's Trace	.005	.200[b]	1.000	44.000	.657	.005	.200	.072
	Roy's Largest Root	.005	.200[b]	1.000	44.000	.657	.005	.200	.072
fatique * groups	Pillai's Trace	.167	2.934[b]	3.000	44.000	.044	.167	8.801	.657
	Wilks' Lambda	.833	2.934[b]	3.000	44.000	.044	.167	8.801	.657

	Hotelling's Trace	.200	2.934[b]	3.000	44.000	.044	.167	8.801	.657
	Roy's Largest Root	.200	2.934[b]	3.000	44.000	.044	.167	8.801	.657

a. Design: Intercept + groups
Within Subjects Design: fatique
b. Exact statistic
c. Computed using alpha = .05

Mauchly's Test of Sphericity[a]

Measure: MEASURE_1

Within Subjects Effect	Mauchly's W	Approx. Chi-Square	df	Sig.	Epsilon[b] Greenhouse-Geisser	Huynh-Feldt	Lower-bound
fatique	1.000	.000	0	.	1.000	1.000	1.000

Tests the null hypothesis that the error covariance matrix of the orthonormalized transformed dependent variables is proportional to an identity matrix.
a. Design: Intercept + groups
Within Subjects Design: fatique
b. May be used to adjust the degrees of freedom for the averaged tests of significance. Corrected tests are displayed in the Tests of Within-Subjects Effects table.

Tests of Within-Subjects Effects

Measure: MEASURE_1

Source		Type III Sum of Squares	df	Mean Square	F	Sig.	Partial Eta Squared	Noncent. Parameter	Observed Power[a]
fatique	Sphericity Assumed	103.492	1	103.492	.200	.657	.005	.200	.072
	Greenhouse-Geisser	103.492	1.000	103.492	.200	.657	.005	.200	.072
	Huynh-Feldt	103.492	1.000	103.492	.200	.657	.005	.200	.072
	Lower-bound	103.492	1.000	103.492	.200	.657	.005	.200	.072
fatique * groups	Sphericity Assumed	4552.123	3	1517.374	2.934	.044	.167	8.801	.657
	Greenhouse-Geisser	4552.123	3.000	1517.374	2.934	.044	.167	8.801	.657
	Huynh-Feldt	4552.123	3.000	1517.374	2.934	.044	.167	8.801	.657
	Lower-bound	4552.123	3.000	1517.374	2.934	.044	.167	8.801	.657
Error(fatique)	Sphericity Assumed	22758.672	44	517.243					
	Greenhouse-Geisser	22758.672	44.000	517.243					
	Huynh-Feldt	22758.672	44.000	517.243					
	Lower-bound	22758.672	44.000	517.243					

a. Computed using alpha = .05

Tests of Within-Subjects Contrasts

Measure: MEASURE_1

Source	fatique	Type III Sum of Squares	df	Mean Square	F	Sig.	Partial Eta Squared	Noncent. Parameter	Observed Power[a]
fatique	Linear	103.492	1	103.492	.200	.657	.005	.200	.072
fatique * groups	Linear	4552.123	3	1517.374	2.934	.044	.167	8.801	.657
Error(fatique)	Linear	22758.672	44	517.243					

a. Computed using alpha = .05

Levene's Test of Equality of Error Variances[a]

		Levene Statistic	df1	df2	Sig.
stancetimepre	Based on Mean	1.510	3	44	.225
	Based on Median	.904	3	44	.447
	Based on Median and with adjusted df	.904	3	34.022	.449
	Based on trimmed mean	1.493	3	44	.230
stancetimepos	Based on Mean	2.486	3	44	.073
	Based on Median	1.507	3	44	.226
	Based on Median and with adjusted df	1.507	3	40.192	.227
	Based on trimmed mean	2.376	3	44	.083

Tests the null hypothesis that the error variance of the dependent variable is equal across groups.

a. Design: Intercept + groups
Within Subjects Design: fatique

Tests of Between-Subjects Effects

Measure: MEASURE_1
Transformed Variable: Average

Source	Type III Sum of Squares	df	Mean Square	F	Sig.	Partial Eta Squared	Noncent. Parameter	Observed Power[a]
Intercept	10401157.580	1	10401157.580	2782.688	.000	.984	2782.688	1.000
groups	15207.148	3	5069.049	1.356	.269	.085	4.068	.336
Error	164463.592	44	3737.809					

a. Computed using alpha = .05

Parameter Estimates

Dependent Variable	Parameter	B	Std. Error	t	Sig.	95% Confidence Interval Lower Bound	95% Confidence Interval Upper Bound	Partial Eta Squared	Noncent. Parameter	Observed Power[b]
stancetimepre	Intercept	321.182	13.313	24.126	.000	294.351	348.012	.930	24.126	1.000
	[groups=1.00]	31.337	19.846	1.579	.121	-8.660	71.333	.054	1.579	.339
	[groups=2.00]	17.464	17.294	1.010	.318	-17.390	52.318	.023	1.010	.167
	[groups=3.00]	7.235	18.431	.393	.697	-29.910	44.380	.003	.393	.067
	[groups=4.00]	0[a]	.	.	.	.	.	.	.	.
stancetimepos	Intercept	324.727	14.477	22.430	.000	295.550	353.904	.920	22.430	1.000
	[groups=1.00]	41.828	21.581	1.938	.059	-1.666	85.323	.079	1.938	.474
	[groups=2.00]	-5.373	18.806	-.286	.776	-43.275	32.529	.002	.286	.059
	[groups=3.00]	13.884	20.043	.693	.492	-26.510	54.277	.011	.693	.104
	[groups=4.00]	0[a]	.	.	.	.	.	.	.	.

a. This parameter is set to zero because it is redundant.

b. Computed using alpha = .05

Estimated Marginal Means

1. Grand Mean

Measure: MEASURE_1

Mean	Std. Error	95% Confidence Interval Lower Bound	95% Confidence Interval Upper Bound
336.251	6.374	323.405	349.098

2. groups

Estimates

Measure: MEASURE_1

groups	Mean	Std. Error	95% Confidence Interval

			Lower Bound	Upper Bound
1.00	359.537	14.410	330.495	388.579
2.00	329.000	10.808	307.218	350.782
3.00	333.514	12.480	308.363	358.665
4.00	322.955	13.035	296.685	349.224

Pairwise Comparisons

Measure: MEASURE_1

(I) groups	(J) groups	Mean Difference (I-J)	Std. Error	Sig.[a]	95% Confidence Interval for Difference[a] Lower Bound	Upper Bound
1.00	2.00	30.537	18.013	.583	-19.229	80.303
	3.00	26.023	19.063	1.000	-26.644	78.691
	4.00	36.582	19.431	.398	-17.101	90.266
2.00	1.00	-30.537	18.013	.583	-80.303	19.229
	3.00	-4.514	16.509	1.000	-50.125	41.098
	4.00	6.045	16.932	1.000	-40.736	52.827
3.00	1.00	-26.023	19.063	1.000	-78.691	26.644
	2.00	4.514	16.509	1.000	-41.098	50.125
	4.00	10.559	18.046	1.000	-39.297	60.416
4.00	1.00	-36.582	19.431	.398	-90.266	17.101
	2.00	-6.045	16.932	1.000	-52.827	40.736
	3.00	-10.559	18.046	1.000	-60.416	39.297

Based on estimated marginal means

a. Adjustment for multiple comparisons: Bonferroni.

Univariate Tests

Measure: MEASURE_1

	Sum of Squares	df	Mean Square	F	Sig.	Partial Eta Squared	Noncent. Parameter	Observed Power[a]
Contrast	7603.574	3	2534.525	1.356	.269	.085	4.068	.336
Error	82231.796	44	1868.904					

The F tests the effect of groups. This test is based on the linearly independent pairwise comparisons among the estimated marginal means.

a. Computed using alpha = .05

3. fatique

Estimates

Measure: MEASURE_1

fatique	Mean	Std. Error	95% Confidence Interval Lower Bound	95% Confidence Interval Upper Bound
1	335.191	6.510	322.070	348.312
2	337.312	7.080	323.044	351.580

Pairwise Comparisons

Measure: MEASURE_1

(I) fatique	(J) fatique	Mean Difference (I-J)	Std. Error	Sig.[a]	95% Confidence Interval for Difference[a] Lower Bound	95% Confidence Interval for Difference[a] Upper Bound
1	2	-2.121	4.742	.657	-11.679	7.436
2	1	2.121	4.742	.657	-7.436	11.679

Based on estimated marginal means

a. Adjustment for multiple comparisons: Bonferroni.

Multivariate Tests

	Value	F	Hypothesis df	Error df	Sig.	Partial Eta Squared	Noncent. Parameter	Observed Power[b]
Pillai's trace	.005	.200[a]	1.000	44.000	.657	.005	.200	.072
Wilks' lambda	.995	.200[a]	1.000	44.000	.657	.005	.200	.072
Hotelling's trace	.005	.200[a]	1.000	44.000	.657	.005	.200	.072
Roy's largest root	.005	.200[a]	1.000	44.000	.657	.005	.200	.072

Each F tests the multivariate effect of fatique. These tests are based on the linearly independent pairwise comparisons among the estimated marginal means.

a. Exact statistic

b. Computed using alpha = .05

4. groups * fatique

Measure: MEASURE_1

groups	fatique	Mean	Std. Error	95% Confidence Interval Lower Bound	95% Confidence Interval Upper Bound
1.00	1	352.519	14.718	322.856	382.181
	2	366.556	16.005	334.299	398.812

2.00	1	338.646	11.038	316.399	360.892
	2	319.354	12.004	295.162	343.546
3.00	1	328.417	12.746	302.729	354.105
	2	338.611	13.861	310.676	366.546
4.00	1	321.182	13.313	294.351	348.012
	2	324.727	14.477	295.550	353.904

Post Hoc Tests

groups

Multiple Comparisons

Measure: MEASURE_1
Tukey HSD

(I) groups	(J) groups	Mean Difference (I-J)	Std. Error	Sig.	95% Confidence Interval	
					Lower Bound	Upper Bound
1.00	2.00	30.5370	18.01284	.338	-17.5574	78.6314
	3.00	26.0231	19.06300	.527	-24.8752	76.9215
	4.00	36.5825	19.43081	.250	-15.2979	88.4629
2.00	1.00	-30.5370	18.01284	.338	-78.6314	17.5574
	3.00	-4.5139	16.50904	.993	-48.5931	39.5654
	4.00	6.0455	16.93242	.984	-39.1642	51.2551
3.00	1.00	-26.0231	19.06300	.527	-76.9215	24.8752
	2.00	4.5139	16.50904	.993	-39.5654	48.5931
	4.00	10.5593	18.04557	.936	-37.6224	58.7411
4.00	1.00	-36.5825	19.43081	.250	-88.4629	15.2979
	2.00	-6.0455	16.93242	.984	-51.2551	39.1642
	3.00	-10.5593	18.04557	.936	-58.7411	37.6224

Based on observed means.
The error term is Mean Square(Error) = 1868.904.

Homogeneous Subsets

MEASURE_1

Tukey HSD[a,b,c]

groups	N	Subset 1
4.00	15	322.9545
2.00	16	329.0000
3.00	12	333.5139
1.00	10	359.5370
Sig.		.193

Means for groups in homogeneous subsets are displayed.
Based on observed means.
The error term is Mean Square(Error) = 1868.904.
a. Uses Harmonic Mean Sample Size = 11.499.
b. The group sizes are unequal. The harmonic mean of the group sizes is used. Type I error levels are not guaranteed.
c. Alpha = .05.
